# Social factors associated with the development of e-health literacy: A mixed-methods study among patient organization members and representatives

**DOI:** 10.1371/journal.pdig.0001569

**Published:** 2026-08-13

**Authors:** Lea Wiedemann, Jan Hinrichsen, Jonas Lander, Simon Wallraf, Marie-Luise Dierks, Henk Jasper van Gils-Schmidt, Sabine Wöhlke

**Affiliations:** 1 Faculty of Health, Hamburg University of Applied Sciences (HAW Hamburg), Hamburg, Germany; 2 Department of Medical Ethics and History of Medicine, University Medical Center Göttingen, Göttingen, Germany; 3 Institute for Epidemiology, Social Medicine and Health Systems Research, Hannover Medical School, Hannover, Germany; 4 Hamburg University of Applied Sciences (HAW Hamburg), Hamburg, Germany; National Tsing Hua University, TAIWAN

## Abstract

The increasing digitalization of healthcare systems presents both opportunities and challenges for patients. A key challenge lies in cultivating e-health literacy, defined as the capacity to locate, comprehend, appraise, and use digital health information and services. This study employs a mixed-methods approach to investigate social and structural factors perceived as shaping patients’ capability to use digital health services. A mixed-method approach guided the design, data collection, data analysis, and synthesis. This included semi-structured interviews and an online survey to corroborate findings and enhance credibility. The objective of the study was to examine the perspectives and dispositions of members and representatives of patient organizations regarding digital health services identifying key factors associated with e-health literacy. Findings indicate that respondents view social factors as crucial. Motivation to engage with digital health services is seen as a pivotal factor to shape the development of e-health literacy. However, motivation is not solely an individual trait; it is shaped by social contexts and trust in digital systems. Ensuring the highest standards of data security and transparency is imperative for cultivating this trust. Furthermore, patients must feel a sense of autonomy regarding their personal health data to engage confidently with digital health services. Structured learning environments, offered by social actors such as governments, patient organizations, and health insurance providers, also play a crucial role. These insights are relevant for practitioners in healthcare and public administration. To develop e-health literacy, it is imperative to establish inclusive learning opportunities, ensure data protection, and cultivate patient empowerment. Government agencies, healthcare providers, and insurance companies should collaborate with relevant stakeholders to design and implement supportive measures that reflect patients’ lived realities. This approach can help ensure that all individuals are equipped to participate meaningfully in a digitalized healthcare environment.

## Author summary

In our study, we looked at how people can better understand and use digital health services. As healthcare becomes more digital, patients need confidence when using online tools, apps or platforms to manage their health. To understand what helps or makes this difficult, we conducted an online survey and interviewed members and representatives of patient organizations. We found that motivation is important, but does not depend only on the individual. People are more willing to use digital health services when they trust them, believe their personal data is protected, and feel in control of their own health information. Learning opportunities can also help people build needed skills, especially when these are offered by trusted organizations such as public institutions, health insurers, or patient organisations. Our findings suggest that improving e-health literacy is not just a technical issue. It requires support from healthcare providers, public institutions, and organizations involved in healthcare and patient support. This includes secure, accessible digital sytems and services, guidance from healthcare professionals that helps build trust, and structured involvement of patient organizations in developing educational and participatory opportunities. Such support can help ensure that everyone can benefit from digital health tools, regardless of their starting point.

## Introduction

Like most public sectors, health care systems are undergoing digital transformation, which varies by country and depends on institutional and structural factors, such as form of democracy and population size [1]. These differences shape the implementation and adoption of digital health services, including electronic health records, applications for disease self-management, and telemedicine. Research highlights benefits of digital health services, including improved health behaviors, patient empowerment, cost reduction, and more patient-centered healthcare [2–6].

A concomitant expectation of developers is that healthcare users can effectively and competently use such technologies. This requires however a sufficient degree of e-health literacy (e-HL), defined as the ability to use digital services to find, understand, evaluate, and use health-related information [7] and commonly measured by eHEALS or its extensions. e-HL is distinct from general health literacy, which emphasizes this ability in any format. However, while digital transformation in medicine continues to advance, access to respective services does not automatically guarantee users’ confidence in its access and use. In Germany, for example, representative studies indicate that e‑HL is concerningly low: Over three-quarters of the population self-report low e-HL, especially older people and those with less education or resources, or with a low social status [8,9]. These groups use digital health services less. Additionally, two-thirds of the participants self-reported severe difficulty finding, understanding, assessing, and applying digital health information [9].

Studies show e-HL is shaped by various factors, including age, socioeconomic status, and educational background [10,11]. Further studies have investigated the relationship between the use of digital health services and e-HL, and vice versa [12–15].

Qualitative studies highlight design opportunities to address facilitators and barriers in using digital health services [16–20]. These studies concluded that low e-HL is a barrier for the use of digital health services. These studies did not assess which factors matter from the patients’ own perspective for feeling able to navigate and use those services.

Despite previous research has examined the factors associated with e-HL extensively, little attention has been paid to patients’ own perspectives. We address this gap by exploring how patients and members of patient organizations (PO) perceive the factors that enable or hinder the effective and competent use of digital health services. POs serve as central and trustworthy points of contact for many patients and boast extensive networks. They provide advice and targeted training on digital health services, help to spread knowledge, build skills, and promote the use of digital health services.

Although e-HL is commonly operationalized using standardized self-report instruments, our study does not aim to quantify individual levels. Instead, we examine how PO members and representatives describe practical prerequisites, social supports, and contextual conditions that make digital health services feel usable, trustworthy, and controllable. We use eHL as an orienting concept to interpret these accounts, while focusing on participants’ everyday language around being able to use digital services. Consequently, we do not report determinants of measured eHL, but identify perceived factors and structural conditions participants consider relevant for becoming competent digital health users. This focus is of particular significance given that incorporating the viewpoint of this pivotal stakeholder group may empower researchers, patient organizations, policymakers, and other relevant parties to develop services and support activities in a more patient-centered manner [see 21,22].

## Method

The study was conducted as part of the multicenter research network PANDORA (“Patient-centered digitalization: An ethical analysis of the role of POs as actors in the context of digitalization in health-related research and care”). PANDORA conducts an analysis of the ethical and social implications of the utilization of e-health services within the healthcare sector, as perceived from the viewpoint of POs. The present study focuses on Germany. Germany is currently experiencing delays in the digitalization of the healthcare sector, which has consequences for the development of e-HL [8,9].

An exploratory sequential design was employed to develop the instruments for this mixed-method study [see, e.g., 23]. To this end, semi-structured interviews were conducted. The gathered data was then subjected to a preliminary analysis, which included the coding of the material according to the qualitative content analysis method (as described below). A subsequent online survey was developed, integrating the themes identified in the preceding interviews (among others on the use of digital health services). The methodological approach employed during the design phase offered several advantages, chief among them being the ability to collect both qualitative and quantitative data on the same subjects [24]. To determine whether the results converged, offered complementary insights, or showed dissonance, we applied triangulation of the qualitative and quantitative findings [25].

Both parts of the study were conducted with participants who had attained a minimum age of 18 years, who were affiliated with a German POs, and who possessed the capacity to provide consent themselves. Participation was voluntary. In the following sections, we present the study procedures and data collection for the qualitative and quantitative parts of the study separately.

### Qualitative part: Study procedures, data collection & data analysis

For the qualitative interviews, participants with prior experience using digital health services were recruited via six German POs operating their own digital patient registry. These registries are used to collect individual health data from the respective members of the POs for research purposes. Three POs agreed to disseminate the study invitation, and one sent a reminder. To ensure the integrity of the study, members provided consent for contact by the research team. Ultimately, 13 participants from two POs (Mukoviszidose e. V. and PRO RETINA Deutschland e. V.) were interviewed between September and November 2022.

Following the established methods for qualitative research in healthcare [26], a semi-structured interview guide was developed to gather personal experiences with and ethical assessments of the digital transformation in healthcare and medical research (see S1 Text). The patient representative advisory board of the PANDORA research project (www.pandora-forscht.de/en/projektbeiraete) reviewed the guide to enhance its comprehensibility and completeness. A preliminary test was conducted to assess its functionality. The interviews were conducted by telephone and recorded using a dictation machine connected to the telephone handset.

The recordings were transcribed verbatim, pseudonymized (see S2 Text for the transcription rules), and coded in MAXQDA by two researchers (HJvGS & SK) using a predefined coding guide (see S3 Text). A preliminary reduction of themes was conducted (HJvGS) and used as input for the development of the online questionnaire. For the present article, three team members (HJvGS, LW, and JH) analyzed the interview material (see S1 Data) to identify overarching themes and agreed on exemplar quotations. The questionnaire provided additional qualitative data through free text responses on digital skills (see description below). These open-ended responses (included in S1 Data) were analyzed using the previously identified overarching themes as a coding guide, while remaining attentive to potential dissonances (SW, JL, LW, JH).

### Quantitative part: Study procedures, data collection & data analysis

Participants were recruited for the online survey through a structured invitation process. We identified a total of 300 POs using membership lists from two major German umbrella organizations: the German National Association for Self-Help (*Bundesarbeitsgemeinschaft Selbsthilfe von Menschen mit Behinderung, chronischer Erkrankung und ihren Angehörigen e.V.* (BAG SELBSTHILFE)) and the Alliance for Chronic Rare Diseases (*Allianz Chronischer Seltener Erkrankungen e.V.* (ACHSE)). Additionally, we included organizations listed in the *Grüne Adressen* directory, maintained by the National Contact and Information Point for Self-Help (*Nationale Kontakt- und Informationsstelle zur Anregung und Unterstützung von Selbsthilfegruppen* (NAKOS)). In parallel to this, we disseminated an inaugural email invitation to the 300 POs, comprising study information and a survey link that could be forwarded to respective members. To encourage participation, we disseminated two follow-up reminders. The invitation was also distributed by our patient representative advisory board, as well as by BAG SELBSTHILFE e.V. and ACHSE e.V.

A questionnaire was developed to examine members’ perspectives on the digital transformation of their POs. The survey encompassed multiple dimensions pertaining to the digitalization processes within these organizations. For this manuscript, we focused on four key survey items (ND04–ND07), alongside the survey’s sociodemographic variables. Item ND04 captured participants’ self-rated ability to use and navigate digital technologies and services, which we refer to as “digital skills.” This item was not intended to directly assess eHealth literacy. We included it because such basic abilities may affect whether individuals are able to engage with digital health contexts at all, regardless eventual levels of e-HL. Items ND05 and ND06 captured challenges participants encountered when using digital technologies and services, which may also limit individuals’ capacity to engage in eHealth-related activities. We therefore consider both digital skills and usage-related challenges to be important contextual information. Item ND07 gave participants the option to provide additional information through a free-text response. Although the items ND04–ND07 were phrased in general terms, they were situated in a survey section introduced by a digital health–related scenario (see S4 Text for the full questionnaire). To ensure comprehensibility and completeness, the questionnaire was reviewed by the patient representative advisory board as well as researchers from other subprojects of the PANDORA research network. The development of the questionnaire was informed by the findings from a preceding scoping review and the qualitative studies conducted within PANDORA [27,28]. A pre-test was conducted with members and representatives of POs to assess clarity and usability, leading to minor refinements before deployment. The survey was administered online using SoSci Survey from August 8, 2023, to November 20, 2023.

The online survey dataset was thoroughly examined for plausibility and completeness. Missing data were identified and reported for each variable. To examine associations between self-rated digital skills and participant characteristics (e.g., age, gender, and educational background), we used bivariate analyses including cross-tabulations and Chi-square tests. For this, an available-case approach was applied. Data cleaning and statistical analyses were conducted by one member of the research team (SW).

### Ethics approval

This study was conducted in accordance with the Declaration of Helsinki and received ethical approval from the University Medical Center Göttingen (application number 11/6/22), Hamburg University of Applied Sciences (application number 2022-20) and the Hannover Medical School (10901_BO_K_2023).

## Results

### Participant characteristics

A total of n = 13 interviews (average length: 54 minutes) and n = 1,334 survey responses, including 431 free-text answers were included in the quantitative and qualitative data analysis.

The demographics characteristics from the survey and interview participants are shown in Table 1. Overall, the survey sample tended to be older, with a higher proportion of participants aged 50 years and above, whereas comparatively more interview participants were in the 30–49-year age range. The survey sample was also predominantly female, while the interview sample included more male participants. Regarding educational attainment, the survey sample showed a broader distribution across educational levels, though the largest share from both samples held higher education degrees. In terms of membership background, most participants in both samples were individuals personally affected by a chronic disease or disability.

**Table 1:** Participant characteristics of the online survey (n = 1,334) and the interview study (n = 13).

	Online survey n (%)	Interview study n (%)
**Age**		
18-29 years	44 (3.3)	1 (7.7)
30-39 years	136 (10.2)	3 (23.1)
40-49 years	198 (14.8)	4 (30.8)
50-64 years	555 (41.6)	4 (30.8)
65 years and older	320 (24)	1 (7.7)
Missing^a^	81 (6.1)	
**Gender**		
Female	896 (67.2)	5 (38.5)
Male	409 (30.7)	8 (61.5)
Diverse	2 (0.1)	–
Missing^a^	27 (2)	–
**Number of indicated diseases and/or disabilities** ^b^		
1	202 (15.1)	–
2-3	444 (33.3)	–
4-5	208 (15.6)	–
≥6	114 (8.5)	–
Not personally affected	330 (24.7)	–
Missing^a^	36 (2.7)	13 (100)
**Educational attainment**		
Basic lower secondary education certificate *(ISCED-2, Hauptschulabschluss)*	64 (4.8)	–
Intermediate lower secondary education certificate *(ISCED-2, Realschulabschluss)*	278 (20.8)	2 (15.4)
Entrance qualification for universities of applied sciences *(Fachhochschulreife)*	123 (9.2)	–
General entrance qualification for universities *(Abitur)*	181 (13.6)	–
Higher education degree	616 (46.2)	10 (76.9)
No formal qualification^c^	3 (0.2)	1 (7.7)
Other qualification	29 (2.2)	–
Missing^a^	40 (3)	–
**Membership Background**		
Affected individual (by disease or disability)	1004 (75.3)	12 (92.3)
Relative of an affected individual	247 (18.5)	1 (7.7)
Other	79 (5.9)	–
Missing^a^	4 (0.3)	–

^a^Missing values include ‘prefer not to answer’ responses and unanswered questions.

^b^This variable is based on a filter question shown only to respondents who indicated that they are members of a patient organization because they are personally affected by a disease or disability. Participants could select all conditions that applied to them. However, multiple selections should not be interpreted as necessarily indicating distinct or unrelated conditions; in some cases, the reported diseases and/or disabilities may reflect different aspects or consequences of a single underlying condition. Accordingly, the results should be interpreted with appropriate caution. A full list of response options is available in the questionnaire included in the S4 Text. Respondents who were not personally affected (e.g., relatives) were treated as missing values for this variable. Nonetheless, they are shown separately in the descriptive results. For the interview study this variable was not collected.

^c^This category includes people who left school without a diploma or who do not yet have one.

The analysis of the online survey data revealed a spectrum of factors that may affect engagement with digital technologies and services. Interpreting the perspectives related to such digital health engagement identified in the qualitative data—the interviews and open-ended survey responses—enabled a deeper understanding of the aspects identified in the quantitative data and provided contextual insights. As apparent from the presentation of findings below, the quantitative and qualitative data mostly confirmed or complemented each other.

### Affording digital health services, motivation and uncertainty

**Fig 1:**
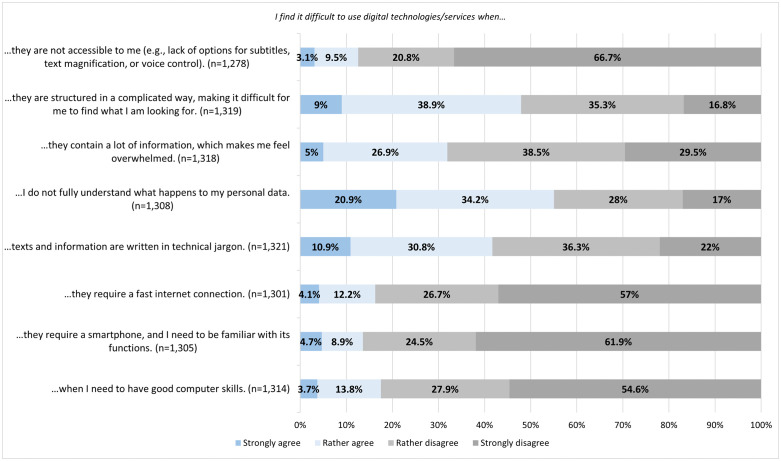
Perceived Difficulties in Using Digital Technolgoies and Services. The figure shows the distribution of responses to the statement “I find it difficult to use digital technologies/services when…”. Note: Percentages are calculated based on valid responses for each item; missing responses were excluded from the visualization to maintain comparability across items. Percentages may not sum to 100% due to rounding.

In general, the online survey revealed specific difficulties in using digital health services. The most commonly reported challenges included difficulties in using a service when the processing of personal data is unclear (55.1%), when the service’s structure is complex (47.9%), and when content is formulated in technical language (41.7%). Likewise, interview participants recalled such challenges. In contrast, the least frequently reported difficulties included the lack of accessibility features, such as subtitles, text enlargement, or voice control (12.6%), the requirement to use a smartphone (13.6%), and the need for a fast internet connection (16.3%) (see Fig 1 for full distribution of responses).

In our sample, self-rated digital skills were predominantly positive: 33.7% of participants rated their skills as “very good” and 53.9% as “rather good” (see Table 2).

**Table 2:** Distribution of self-rated digital skills among survey participants (n = 1,334).

Digital skill rating	n (%)
Very good	449 (33.7)
Rather good	719 (53.9)
Rather poor	136 (10.2)
Very poor	15 (1.1)
Missing^a^	15 (1.1)

^a^Missing values include ‘prefer not to answer’ responses and unanswered questions.

The bivariate analyses showed significant associations between self-rated digital skills and several participant characteristics, including gender, age, and educational attainment. Among male respondents, 41.5% rated their skills as very good, compared to 30.8% of female respondents. Participants aged 18–29 and 30–39 most frequently reported very good digital skills (63.6% and 55.1%, respectively), whereas this proportion declined markedly among older age groups, with only 30.1% of 50–64-year-olds and 19.4% of those aged 65 and older rating their skills as very good. A similar trend was observed regarding educational attainment: participants with higher educational qualifications more frequently reported very good digital skills than those with lower qualifications (see S2 Data and Table 1 for full results of the bivariate analyses). Such differences were also reflected in the interviews, where those identifying as digital natives or expressing a strong interest in technology less frequently reported difficulties in using digital health services.

Building on these findings, our qualitative interview data points to a broader set of conditions required for users to actively engage in digital environments, including the perceived affordances of digital technologies. This broader perspective includes both structural determinants—at the social and political level—and individual-level determinants chronically ill people consider relevant for developing e-HL.

On an individual level, a driving force of developing e-HL is the interest and willingness of users themselves to participate in digital health environment and to actively engage with digital health services. Many of them expressed that motivation is one prerequisite for e-HL. This, in turn, is connected to a self-assessment of one’s own ability to engage with digital health technologies, to practice and to learn:

“So of course you have to be open to technology; I know some people […] who are rather reserved about it. And although I would trust them to do it, their interest is not so great [...] and I always say:[…] you have to deal with it because it will come at some point. It will be standard […]and then you sit there and don’t know it.” (Patient 1)

This motivation, however, sometimes sharply contrasts with an uncertainty about new technologies.

Many interviewees stated to be highly insecure in navigating digital environments, predominantly due to its perceived novelty. Therefore, they felt insufficiently exposed to a routine use of digital communication, which, to them, both results from and leads to distrust (see also the theme “age”):

“the expertise to deal with electronic gadgets […] that’s a very, very difficult subject. I am not very good at it myself, so I have very poor IT access on my own, but I have a husband who helps me with it (laughs) and I can use it, but I’m not someone who can figure out applications on my own. So if someone shows me how to do it, I can use it logically[…] but I have real difficulties using it myself, […]. Most things are not self-explanatory for me.” (Patient 2)

In other words, our interviewees pointed to the challenge faced by a significant group of people lacking sufficient routine and sophistication to develop their e-HL for competent use of digital health services.

Due to lack of experience and insufficient support, some interviewees felt insecure when dealing with digital health services whereas others, who described themselves as digitally adapted, reported no feelings of insecurity. Many interviewees did confirm that people need to have motivation to develop e-HL.

The online survey adds another perspective on this uncertainty as a barrier to developing e-HL, insofar as it showed a reluctance to actively engage in the development of digital health services. Of those who stated to be unwilling to actively get involved in the planning and design of digital health services in their PO (n = 587), 43.3% reported that their reluctance is due to uncertainty about whether their technical knowledge is sufficient.

Our interviewees also emphasized this:

“in my private environment, again the two extremes: me, who has to do with it professionally (...) and tries out a lot more and looks closely a lot more and also knows what she has to look out for a bit and my mother-in-law who has nothing to do with it at all [...] because it’s all bad.” (Patient 3)

### Accessiblity of digital services

Further, from the point of view of the PO participants, the development of their own e-HL—and therefore also their motivation—depends on the accessibility of digital services. The online survey painted a different picture as a lack of accessibility features—such as subtitles, text enlargement, or voice control—was not perceived as an issue by most: 66.7% stated that a lack of accessibility features does not pose any difficulty for them at all. Another 20.8% indicated that such a lack is rather not an issue for them. In contrast, 9.5% said that the absence of these accessibility features is somewhat of a problem, while only 3.1% strongly agreed that this limitation affects them (see Fig 1). This smaller group mirrored what was also found in the interviews: making digital health services accessible for all is a crucial path to greater inclusion. Accordingly, one interviewee described how digital services served as an aid for them:

“[…]For me, the digital is simply a tool to keep in touch with the outside world, so especially for visually impaired people, the smartphone or tablet is one of the most important tools you have [...] so I have a huge screen so that I have a certain magnification, I have a screen reader that reads it to me, I have learned to write (...) […] (...) blind (...) and that helps of course.” (Patient 3)

In terms of accessibility, interviewees also addressed sociodemographic barriers, such as chronic diseases and illnesses. They elaborated on how digital health services tend to be unsuitable for people suffering from chronic disease limiting their motor skills, e.g., rheumatism or people with visual impairments:

*“*there is the issue of accessibility. […] So the fact that many people, for example the visually impaired, are prevented from using these electronic options appropriately and this must definitely be taken into account in medical applications, in EVERY application*.”* (Patient 2)

Therefore, from the perspective of the PO members, digital health services should be designed to account for accessibility, with particular consideration to people with disabilities, such as visual impairment:

“The design of websites should also take account of the visually impaired. E.g. with read-aloud functions, high-contrast font, clear structure.” (response to the topic “use of digital media and applications” [ND07], online survey)

This aspect was also addressed in the interviews:

“[…]It should also be produced in such a way that people can use it, for example, voice output/voice input. Or people who […] have a mental disability who perhaps get it in this easy language[…]?” (Patient 1)

### Equal access to digital health services: Costs, infrastructure and opportunities

Furthermore, the empirical data showed that accessibility is strongly related to equal access. According to the respondents, equal access included, among other things, access to a device and a good internet connection. Although our survey data indicated that only 13.6% of respondents have difficulties using digital services due to access to a device (smartphone), with 16.3% having difficulties due to a slow internet connection, the interview material highlighted the various facets related to equal access: For instance, interviewees, as well as the open ended answers in the online survey, showed that access to digital health services was limited by the high cost of necessary devices, creating barriers for those with low financial means, particularly individuals unable to work due to illness:

“In my environment, I know people who can’t afford technology that is far too expensive (cell phones, laptops, PCs). As a result, they don’t get any practice with it. I think that’s also a kind of exclusion.” (response to the topic “use of digital media and applications” [ND07], online survey)

Moreover, slow internet connection is linked particularly to more rural areas:

“Of course, somehow the access, […]. So depending on where you live, […]. [I: By access you mean, for example, internet access, but also hardware access and so on?] Right, exactly. Then the financial aspect also plays a role again. Being able to buy something like that at all.” (Patient 4)

The interviews emphasized that digital health services can enhance access equity, especially for individuals in rural areas or with limited financial means, by providing alternatives to physical travel:

“[…] people are able to look up information about such offers independently, for example on the Internet, and then take advantage of them [...] that everyone who knows that this offer exists should also be able to take advantage of it (...) especially for people who don’t have a car or can’t afford to take the train to the doctor (...).“ (Patient 5)

### Aging related concerns and the need for broader social support to enhance digital literacy

Interviewees frequently linked these challenges to questions of age and ageism, drawing a direct connection between users’ age and their ability to competently use digital health services. Higher age was often perceived as reinforcing insecurity, particularly due to cognitive or experiential barriers:

“[…] they have to [… be] perhaps fit from a mental point of view, so yes, there are also many older people who perhaps want that but can no longer understand it because age is something […], and because you have to take them into account in some way too.” (Patient 1)

In this context, interviewees emphasized the need for support and assistance, especially when navigating the process of learning to use digital technologies: “I lack confidence in handling. I would need someone I could practice it with. I find the whole thing exciting and would like to master it.” (response to the topic “use of digital media and applications” [ND07], online survey)

Overall, the development of e-HL was described as strongly dependent on available social support. Predominantly, such assistance was found in the social environment of the participants of PO, given by, e.g., close family members:

“so at least the people I know are of course all older, so there are a lot of older people, and they have much less contact with digital processes than I do now, they are simply very insecure. [...] And as I said, I wouldn’t trust myself to do it without my husband.” (Patient 2)

Interviewees also assigned responsibility to actors within the health care system, such as patient organizations, health insurance companies or general practitioners. In this sense, training thorough information and assistance were demanded:

“I would like to see internal PC training from the self-help organizations, especially for older people.” (response to the topic “use of digital media and applications” [ND07], online survey).

Our interviewees also emphasized that healthcare providers as well as policymakers are responsible for supporting individuals in developing their e-HL, while promoting personal use of digital health services:

“I can see that adult education centers and so on may offer something like that, for example. they used to offer cell phone training courses for older people, which I think is very good, it may be that they also have electronic health applications on the subject, [...] The state neglects it criminally. I don’t know that there’s anything going on and the self-help organizations do it on their own responsibility and [...] that’s supported, so they develop their own projects and that’s supported, but of course it’s completely voluntary.” (Patient 2)

Online survey participants linked their insecurity not only to individual factos, but also to insufficient digital engagement and support by healthcare providers and insurance companies. For instance, not all health insurance companies or general practitioners offer digital health services or communication options at all. It was emphasized in the material that digitalization is a two-way street that requires not only the users themselves, but also service providers and how those create and support a competent handling of digital tools:

“Many doctors etc. do not use digital media, so digital communication is hardly possible.” (response to the question about difficulties using digital media and/or services in general [ND06], online survey)

If health insurance companies or physicians do not offer digital health services or cannot provide them to users via appropriate support, this also represents a significant barrier:

“So I don’t know about the health insurance companies, they make their electronic patient files available and say now you can download them and so on, but I don’t see any training on what EPF [electronic patient file] means (offered) with the health insurance companies (…).” (Patient 2)

Finally, interviewees emphasized that physicians not only need to be digitally literate themselves, but should also take the time to explain the used digital tools to patients in a way that is understandable:

“Competence to understand what the computer delivers, of course, this is only possible via the doctor, who hopefully, if he has the time, explains it to the patient precisely enough and in a differentiated way.” (Patient 6)

### Lack of transparency and mistrust in data use and dissemination

Individual uncertainty and the resulting need for support were closely linked to two interrelated factors: limited individual competence in using digital technologies and a general mistrust toward digital technologies, stemming from the experience that such technologies often do not function reliably:

“Yes, the causes of mistrust are (...) on the one hand, of course, my technical understanding of how the technology works and that nothing works perfectly.” (Patient 7)

Many participants emphasized that transparency in digital health services is both necessary and possible, especially concerning the means of data access, collection, usage, and protection. They also wished for more control over their data. Transparency was also seen as essential to building trust in digital health services. Many users struggled with building trust when the motives of the provider are unclear:

“Trustworthiness is not always easy to assess.” (response to the question about difficulties using digital media and/or services in general [ND06], online survey).

In addition, study participants were concerned about understanding the scientific validity of digital sources, which influences the willingness to rely on digital health platforms:

“I’m not interested in these things because I can’t understand which sources are being used.” (response to the question about difficulties using digital media and/or services in general [ND06], online survey)

Hidden motives, such as undisclosed advertising, and unverifiable claims of data anonymity further undermine trust. Interviewees emphasized that clear guidance, transparency in data use, and control over personal data are essential for fostering trust in digital health services.

“First and foremost, of course, technical understanding, so on the one hand you have to know what the devices can do (...) but you have to understand (...) what data is collected and what purpose it serves and also the corresponding risks, so you don’t just have to explain the positive things, but also the negative things that could be read from such data.” (Patient 7)

While we were able to identify a need for information and transparency, we also observed how digitalization was problematized as an excess of collected and distributed information. In other words, our interviewees feared becoming too transparent, both to others (e.g., health insurance companies) as to themselves:

“Well, I first said that I’m one of those patients who don’t want to have everything treated and that I want to make my own decision (...) for myself (...) so that I want this decision to stay with me. And of course that also means that I say no, I’m not going to get any information about it and I’m not going to look up every skin condition on the internet to see what it is and I’m not going to worry about this symptom now.” (Patient 2)

Similarly, some participants of POs expressed a preference for limited engagement with their health data, alluding to their right to not knowing (see discussion):

“(...) so of course you have to deal with that, so of course you also have to deal with the group when someone says I don’t want to know exactly what the findings say, I don’t want to know exactly what’s wrong with it, I (...) I don’t want to know that exactly, I just want to know when I have to take which tablets and that’s it.” (Patient 3)

In close relation to this, some interviewees tackle the excess of digital information by prioritizing their body knowledge over external, data based, digital information as provided and processed by digital health services:

“I don’t have to chase after every physical symptom and check whether it’s still within the normal range or whether it’s already a new illness. […] I reserve the right not to pursue certain diagnoses or certain symptoms, I decide whether I want treatment, a diagnosis and treatment or not, and […] to make a well-founded decision about that if possible.” (Patient 2)

### Importance of data security and data sovereignty

Lastly, e-HL was linked to data security by emphasizing the importance of protecting health-related data against loss but also unauthorized use and processing, since health data in particular are highly sensitive:

“Data protection and transparent hosting are important, as the data may be particularly sensitive.” (response to the topic “use of digital media and applications” [ND07], online survey)

This is about protecting data, both in terms of access and storage, and about preventing it from being passed on and misused for manipulation. As stated before, more than half of the respondents in the online survey experienced difficulties using digital health services when not knowing exactly what happens to their personal data. Specifically, this fully applies to 20.9% of participants and rather applies to 34.2%.

Furthermore, from the participants’ point of view, it is not only important that data is protected, but also that they themselves have control over their data; thus they tie e-HL directly to data sovereignty.

“Control over my data so that I can decide who can see what and who gets what.” (response to the topic “use of digital media and applications” [ND07], online survey)

According to the interviewees, technical measures such as splitting data between different providers or strictly separating personal identifiers (e.g., name, address) from medical information can be implemented to minimize the risk of misuse. Measures such as anonymization, pseudonymization, and comprehensive data protection concepts can also help to build trust:

“Of course it’s important to have trust in it and that’s why we’ve put a lot of energy into this anonymization, pseudonymization and so on, and into the data protection concept so that we can give all patients this security and we also have an external service point that you can contact if you have any concerns about your data (…).” (Patient 4)

Data security was thus not primarily identified as a technical challenge. Rather, it was seen as a basis for the trust of the participants of our study, who provide their data.

## Discussion

Our study offers insights into the factors that participants of POs—individuals grappling with chronic illness or disability on a daily basis—perceive as essential for fostering their e-HL. Factors revealed by our study include (technical) accessibility, motivation and the importance of social support, autonomy, transparancy and trust, data security and sovereignty, and equal access. Consequently, we are in a position to address the challenges associated with e-HL that have been identified in various studies examining the utilization of digital health services [17–20]. By conceptualizing the challenge of developing e-HL that patients encounter, we provide a basis for understanding how institutions, such as healthcare providers and lawmakers/governments, can enhance the capacity of citizens, particularly healthcare patients, to utilize digital health services [compare 21,22].

As indicated, one pivotal element in the development of e-HL is the motivation of the recipients of digital health services to proactively engage with innovative technologies and to navigate digital environments. This aspect has not been sufficiently addressed in the literature. Our triangulated data confirm that this motivation does not represent an individual characteristic. Conversely, the interviewees and respondents in the online survey attribute their motivation to both internal and external factors, including aptitude and social support [see 14], as well as technical transparency and accessibility. It is evident that interest and willingness should not be regarded exclusively as a matter of individual motivation. Rather, these phenomena are shaped by a complex interplay of personal, social, economic, and infrastructural determinants, most prominently accessibility and equal access. This interplay illustrates the strength of our triangulated approach: while the survey data quantified the proportion of respondents (13.6%) who experienced difficulties using digital services due to a lack of access to a device (smartphone), the qualitative data provided a nuanced understanding of the underlying factors and personal experiences shaping these challenges. Examples were hight costs of required devices and the availability of reliant internet connections, particularly in rural areas. Accordingly, interviewees engage in critical reflection on the concept of equal access and its determining factors by sharing not only personal experiences of (lacking) equal access, but also their general personal opinion on the matter and perspectives from other participants of PO.

According to our respondents, it is also important that individuals with disabilities or chronic illnesses, the elderly, those facing financial constraints, and rural populations be accorded priority in facilitating accessibility and usability of digital services. Accordingly, our findings are consistent with extant research that has demonstrated the impact of social factors, including age [29], socioeconomic status, and educational background, on patients’ e-HL [10,11]

Our participants reflect on and problematize the requirements users have to fulfill to be actively engaged in digital environments as well as the associated affordances of digital technologies. Questions of accessibility are often interrelated with impairments (e.g., visual) or age. Both the interviewees and survey respondents articulated a discernible aspiration for assistance, thereby translating an external imperative—the necessity of mastering digital technologies—into a claim for and entitlement to support in achieving this objective. In this context, local governments and healthcare providers are regarded as having a mandatory responsibility to provide such assistance.

Ensuring accessibility, however, is not merely relevant for the governance of digital health services; it is also pertinent to direct exchange with questions of motivation. Insufficient accessibility limits the motivation to engage with digital services. Furthermore, it is considered to be both the origin and product of insecurity. In our analysis, insecurity takes a twofold form: habitual-practical insecurity and infrastructural-operational insecurity. The latter is particularly noteworthy in its articulation of a clear desire for data protection, transparency, and control—what is termed “data sovereignty.” The efficacy of e-HL can be enhanced if data protection is ensured through collaborative endeavors by political and societal actors, thereby fostering a sense of safety and trust among patients. Data sovereignty and e-HL are inextricably linked, with each being a prerequisite for the other. The effective utilization of digital technologies is contingent upon the possession of adequate control over personal data, and vice versa.

A critical examination is imperative to ascertain the extent to which the opportunity to acquire additional health-related information through digital technologies directly reinforces the right to self-determination and autonomy. As discussed in the context of transparency issues, some participants of POs express concerns about the potential consequences of the proliferation of e-health services, including the possibility of becoming overly transparent to both external parties, such as health insurance companies, and to themselves. Those being concerned about an excess of information do not wish to be inundated with information about themselves. They emphasize that they do not feel compelled to be omniscient. This insistence on the right to remain unaware is particularly pronounced in the context of digital diagnostic health tools. In order to ensure the autonomy of patients, it is imperative to safeguard the right of the individual to refuse the disclosure of personal data. This prerogative enables individuals to exercise autonomy in determining the utilization of their personal information. Consequently, while autonomy is conventionally linked to the right to know, the accessibility of information, and the capacity to make decisions based on that information, there is no universal obligation to be aware of all aspects of one’s own health status. Consequently, individuals possess an inherent right to remain uninformed [30]. Those who raise concerns about the implications of digital technology may be correct in asserting that it has the potential to infringe upon individual autonomy and self-determination.

From this perspective, e-HL appears to be not only the ability to use digital health services in an adequate manner. It also includes a personal and private information policy that revolves around autonomous decisions about what information an individual wants and does not want, in what way and on which platform or medium.

However, from the perspective of our participants, this information policy is not merely a conscious act but also a corporeal practice. Some interviewees contrast the health information processed and circulated in digital environments (e.g., artificial intelligence tools for diagnoses or recommendations of health tracking apps, both of which are based on the assessment of personal health data) with a bodily/visceral feeling of health and disease. For these interviewees, the interplay between digital insights and bodily knowledge is not merely about rejecting external data; it is about preserving autonomy in health-related decisions.

Such health-related autonomy is a decisive factor for patient participation, and the emergence of digital technologies in healthcare systems has created a significant opportunity to improve it. Digital health services have the potential to enhance patient involvement in their own care in various forms: such as shared decision-making [31], improved access to medical records [32], facilitated communication [33], and the provision of educational resources and online support communities [34]. These services can provide patients with greater access to their medical records, thereby improving transparency and patient literacy. However, the efficacy of such initiatives in achieving targeted support is contingent upon the patients’ e-HL, defined as their capacity to utilize digital health services in an effective and competent manner.

In this regard, POs play a significant role in the development of e-HL as multipliers and important points of contact for patients seeking advice and further training in digital health services. They are also pivotal in the development of e-HL, thereby facilitating the engagement of patients they represent in a digital health environment.

### Relevance for practitioners

The results and their subsequent discussion are directly relevant for practitioners in both local government and healthcare sectors. One promising approach to enhancing e-HL—which empirical evidence suggests is often overestimated in self-assessments [9]—is to focus on motivation. In this context, motivation is understood as a socially embedded phenomenon. Building on this, it is crucial to ensure transparency and main stringent data security standards, as these are key to fostering user trust, which in turn influences user motivation. Moreover the role of patient empowerment in the use of digital health services, and consequently, in the development of e-HL, depends on individuals feeling that they have a good degree of control over their health data. In addition, the development of learning environments to support e-HL is an area of growing interest. One example is the KundiG Program of the Patient University at Hannover Medical University, which aims to improve e-HL of patients with low levels of e-HL [35]. Finally, government agencies and healthcare organizations, including insurance companies, should collaborate with POs to enhance their e-HL.

### Strengths & limitations

Our study contributes to closing existing research gap regarding the views and attitudes of those individuals who use digital health services towards the factors associated with e-HL and the corresponding affordances of digital e-health services. We used a robust mixed-methods design to generate new insights about the social factors associated with developing e-HL. The survey was informed by prior qualitative interview studies, following an exploratory sequential design [see, e.g., 23]. Further, we applied triangulation during the data analysis to examine similarities, differences, themes, and discrepancies across data sources [25]. Regarding self-assessed e-HL, we identified a discrepancy between the qualitative and quantitative data. This phenomenon can be partly attributed to the demographic diversity of the online survey sample, which included a wider range of individuals with varying levels of personal experience with chronic diseases. Consequently, the perspectives of individuals with specific chronic conditions may have been underrepresented. Additionally, as individuals with low digital access or literacy were likely excluded due the digital recruitment and survey format, the participants’ existing digital literacy levels may have influenced their e-HL. The small number of individuals in the survey data, who ascribe themselves a low digital literacy, may also have been influenced by the fact that individuals with very limited access to digital tools or very low digital literacy may not have been able to participate at all. This may have resulted in a selection bias, leading to individuals with very limited digital skills being underrepresented. Notably, our study focused on members of POs in Germany rather than the general public. This helped collect data from individuals who regularly interact with the healthcare system and use its digital health services. Concurrently, it offered minimal insight into the perspectives of individuals who are not or only infrequently in contact with digital health services regarding the factors associated with e-HL.

## Conclusion

This study demonstrates that a complex set of social, motivational, and infrastructural factors shapes an effective and competent use of digital health services. Motivation, while central, is not an isolated trait but influenced by accessibility, trust in digital systems, and the ability to exercise control over personal health data. Data sovereignty emerges as a key condition for fostering autonomy and engagement with digital health services.

Furthermore, POs may play a vital role in supporting e-HL development, acting as multipliers and trusted intermediaries. Their involvement is essential for inclusive learning environments and for equitable access, especially for vulnerable groups such as the elderly, individuals with disabilities, and those in rural or economically disadvantaged areas. Thus, to advance e-HL, stakeholders in the health care and government sectors can find trusted partners in POs to build transparent, secure, and empowering digital infrastructures that respect patient autonomy and promote meaningful participation.

Lastly, our mixed-methods approach revealed a discrepancy between qualitative and quantitative data on self-assessed digital skill, likely influenced by sample composition and digital access. This underscores the need for inclusive strategies and targeted support. As the study is based on a PO-affiliated population with comparatively higher digital access, these findings should be interpreted within this context. Future research should expand to include individuals with limited or no access to digital health services to gain a broader understanding of e-HL challenges in the general public.

## Supporting information

S1 Text Semi-structured interview guide. (PDF)

S2 Text Transcription rules. (PDF)

S3 Text Coding guide. (PDF)

S1 Data Coded segments of the interviews and the open text answers used for the qualitative analysis. (PDF)

S4 Text Online survey questionnaire. (PDF)

S2 Data Bivariate analyses of self-rated digital skills and participant characteristics. (PDF)

S3 Data Dataset used for the quantative analysis. (PDF)
