# Supplementary material for: Social factors associated with the development of e-health literacy: A mixed-methods study among patient organization members and representatives
Source: PLOS Digit Health. 2026 Aug 13;5(8):e0001569. doi: 10.1371/journal.pdig.0001569 (PMC13472428; doi:10.1371/journal.pdig.0001569)
Supplement: Supplementary file 1 [file pdig.0001569.s001.pdf]

Interview guide_Members of Patient Organisation (German original)

Hochschule für Angewandte Wissenschaften Hamburg, 21033 Hamburg
Departement Gesundheitswissenschaften, Ulmenliet 20, 21033 Hamburg

Projektleitung: Prof.'in Dr. Sabine Wöhlke

Universitätsmedizin Göttingen, 37099 Göttingen
Abteilung Ethik und Geschichte der Medizin, Humboldtallee 36, 37073 Göttingen

Projektleitung: Prof.'in Dr. Silke Schicktanz

Projektmitarbeiter: Dr. Henk J. van Gils-Schmidt, B.Sc.

Ulmenliet 20, 37073 Hamburg **Adresse**
040 428 75-6262 **Telefon**
Henk.vanGils@HAW-Hamburg.de **E-Mail**

19. September 2022 **Datum**

Interviewleitfaden
Digitalisierungsprojekte in der medizinische Forschung und Versorgung

Patientenorientierte Digitalisierung: Eine ethische Analyse der Rolle von Patientenorganisationen als Akteure im Zusammenhang mit der Digitalisierung in der gesundheitsbezogenen Forschung und Versorgung (PANDORA)

Kursivdruck = Nachfragen, die nur dann gestellt werden, wenn die vorhergehende Antwort der interviewten Person sehr kurz bzw. wenig informativ ausfällt.

Blau markierte Fragen sind nur für Personen, die sich aktiv für die Datenspende entschieden haben.
Gelb markierte Fragen sind nur für Personen, die sich aktiv gegen die Datenspende entschieden haben.

Vor dem Interview:

- Begrüßung, Interviewer*in stellt sich vor (sehr kurz)
- nochmalige Darstellung des für das Interview zur Verfügung stehenden Zeitrahmens der/des Interviewten
- nochmalige Information über die Aufnahme des Interviews und den vertraulichen Umgang mit Informationen
- (sofern noch nicht erhalten) Bitte um Zusendung der unterschriebenen Einwilligungserklärung
- nochmalige Erläuterung zum Hintergrund der Studie
- Bitte, einen kurzen Fragebogen (bzgl. Demographischer Daten) auszufüllen. Hinweis darauf, dass Antworten anonym bleiben und die*der Interviewer*in diesen Fragebogenvor dem Interview nicht einsehen kann.
- Erläuterung: Interesse der Forschenden an persönlichen Sichtweisen, Einstellungen und Bewertungen; Bitte um persönliche Einschätzung (als Erfahrene*r). Kein richtig oder falsch

A) Einführung: Persönlicher Hintergrund

1) Möchten Sie sich kurz vorstellen?
    a. *Name, Wohnort, Tätigkeit*

B) Chancen und Risiken von digitalen Anwendungen in der Medizin (allgemein)

- Erläuterung: Zu Beginn einige allgemeine Fragen bzgl. Digitaler Anwendungen im medizinischen Bereich.

2) Was bedeutet für Sie „Digitalisierung“ in der medizinischen Forschung und Versorgung? Welche konkreten Beispiele kennen Sie bzw. mit welchen haben Sie in der Vergangenheit zu tun gehabt?
    a. *Ggf. nachfragen: Welche E-Health Produkte würden Sie unterstützen?*
    b. *Ggf. nachfragen: Welche Chancen aber auch Risiken sehen Sie in der Nutzung solcher digitalen Produkte?*
    c. *Ggf. nachfragen: Wird ein Unterschied zwischen Forschung und Versorgung wahrgenommen?*
    d. *Ggf. als Erklärung geben: E-Health, meint sinngemäß übersetzt „Gesundheit basierend auf elektronischer Datenverarbeitung“ und steht als Oberbegriff für die Digitalisierung im medizinischen Bereich. Mit E-Health wird diejenige Kategorie von medizinischen Anwendungen und Maßnahmen bezeichnet, die sich auf die digitale Unterstützung oder digitale Verlagerung von medizinischer Diagnostik, Behandlung und Versorgung richtet. E-Health-Produkte können von privatwirtschaftlichen Unternehmen, von Vereinen und Organisationen sowie von der öffentlichen Gesundheitsversorgung angeboten werden.*

3) Was verstehen Sie unter Gesundheitskompetenz? Und welchen Grad an Kompetenz erachten Sie als notwendig, damit Sie bzw. andere Personen sich an digitalen Gesundheitsprojekten und -technologien beteiligen können?
    a. Ggf. erläutern: Gesundheitskompetenz kann verstanden werden als das Wissen, die Motivation und die Fähigkeiten von Menschen, relevante Gesundheitsinformationen zu finden, zu verstehen, zu beurteilen und im Alltag anzuwenden.

4) Mittlerweile wird ein Großteil an Kommunikation mit dem Handy, Tablet etc. durchgeführt. Neben der Gesundheitskompetenz ist damit auch eine digitale Kompetenz immer wichtiger. Wie würden Sie digitale Kompetenz beschreiben? Welchen Grad an Kompetenz erachten Sie als notwendig, damit Sie bzw. andere Personen sich an digitalen Gesundheitsprojekten und -technologien beteiligen können?
    a. Ggf. erläutern: Digitale Kompetenz ist das Wissen, die Motivation und die Fähigkeiten von Menschen, am digitalen Alltag teilzunehmen und mit digitalen

Anwendungen umzugehen, mit ihnen zu lernen, zu arbeiten, und sie kritisch zu bewerten.

5) Welche Verantwortung haben aus Ihrer Sicht beteiligte Organisationen bei der Umsetzung von digitalen Gesundheitsprojekten? Beteiligte können z.B. PO/SHO, private Unternehmen, die Wissenschaft oder die Politik sein. Würden Sie den unterschiedlichen Beteiligten unterschiedliche Bereiche zuweisen? Worin besteht ggf. der Unterschied?

C) Chancen und Risiken von digitalen elektronischen Patientenregistern

- Erläuterung: Ich möchte gerne zum Thema „Patient*innenregister“ einige Frage stellen.

6) Wie bewerten Sie die Initiative ihrer PO/SHO ein Patient*innenregister zu eröffnen?
    a. *Bei Muko-Mitglieder nachfragen: Haben Sie Informationen und Materialien von Ihrer Ärztin bzw. Ihrem Arzt erhalten, um Sie über die Teilnahme am Patientenregister zu informieren? Stehen diese Informationen auf der Internetseite der PO zum Nachlesen zur Verfügung?*
    b. *Ggf. Nachfragen: wichtigste Gründen sich dafür / dagegen zu entscheiden*
    c. *Ggf. nachfragen: Einschätzung von Gesundheitsnutzen*
    d. *Ggf. nachfragen: Nutzen für die Gesellschaft bzw. Gemeinschaftsgefühl für die Gemeinschaft der Betroffenen*
    e. *Ggf. nachfragen: Möglichkeit zur Kontrolle/ Bestimmung über Ihre Daten und Ihre Nutzung bzw. die Rolle von Datensicherheit*

7) Inwiefern bringen Patient*innenregister Chancen und Herausforderungen mit sich, weshalb Personen Ihre Zustimmung zur Datennutzung abgeben oder auch nicht?

8) Welche Informationen und Materialien haben Sie von Ihrer Ärztin bzw. Ihrem Arzt erhalten, um Sie über die Teilnahme am Patient*innenregister zu informieren?
    a. *Ggf. nachfragen bei Mitglied des Mukoviszidose e.V.: der Mukoviszidose e.V. hat auf seiner Homepage die Dokumente zum Register zur Verfügung gestellt. Wurden Ihnen diese Dokumente durch den behandelnden Arzt bzw. die behandelnde Ärztin ausgehändigt?*
    b. *Ggf. nachfragen: Gibt es weitere Informationen, die Sie sich noch gewünscht hätten?*

9) Wir haben schon über Verantwortung gesprochen, die eine PO/SHO bei der Umsetzung von E-Health-Initiativen übernehmen sollte.
   Welche konkreten Maßnahmen bei der Umsetzung eines Registers halten Sie für wichtig?
    a. *Ggf. nachfragen: Geht es hier um Datenschutz, um die Verwendung der Daten oder auch darüber, was damit beforscht wird?*
    b. *Mögliche Akteure, die explizit erwähnt werden können (siehe Frage 5): SHO/PO, private Unternehmen, die Wissenschaft, die Politik*

10) Die Zustimmung, persönliche Daten an ein Patient*innenregister zu spenden, kann auf unterschiedlichen Wegen erfolgen. Entweder werden die Daten für eine spezifische Studie verwertet oder für ein breites Spektrum an zukünftigen Forschungsprojekten, deren Inhalt sich zum Zeitpunkt der Zustimmung jedoch noch nicht absehen lässt. Wie stehen Sie zu dieser weiten Zustimmung (sog. broad consent)?
    a. Welche Nachteile/ Vorteile sehen sie darin?
    b. *Ggf. Nachfragen: Gibt es Maßnahmen, womit Ihr Sicherheitsempfinden hinsichtlich der Nutzung Ihrer persönlichen (sensiblen) Daten im Kontext einer weiten Zustimmung gestärkt werden kann?*

11) Gibt es irgendwelche Erfahrungen und Einstellungen, die Sie ansprechen möchten, die bisher noch nicht angesprochen würden?

Ausstieg aus dem Interview:

- Einladung: Wir würden uns sehr freuen, wenn Sie weiter am Projekt PANDORA mitwirken würden! Interessiert sind wir insbesondere an Themenvorschlägen für (Video-)Podcasts, die wir ggf. über unsere Website veröffentlichen würden. Gerne kann auch nach Interview Kontakt zu Projektmitarbeiter*innen aufgenommen werden.

- Verabschiedung

- Verweis, dass jederzeit mit uns Kontakt aufgenommen werden kann, falls Fragen etc. auftreten

- Hinweis darauf, dass die Interviewaufnahmenentsprechend den Datenschutzrichtlinien konform behandelt werden

Fragebogen zu demografischen Daten

Bitte geben Sie Ihr Geschlecht an.

☐ Männlich ☐ Weiblich ☐ Divers

Bitte geben Sie Ihre Alter an:

… Jahre

Von welcher Patient*innenorganisation(en) sind Sie Mitglied?

………………………………..

In welcher Beziehung stehen Sie?

☐ Patient*in

☐ Familie von einer*einem Patient*in

☐ Versorgende von einer*einem Patient*in

☐ Bekannte von einer*einem Patient*in

Was ist Ihr höchster Schul- oder Hochschulabschluss?

☐ kein Abschluss

☐ Hauptschulabschluss

☐ Realschulabschluss

☐ Hochschulreife/Abitur

☐ sonstiges

Was ist Ihre höchste Berufsausbildung?

☐ keine Ausbildung

☐ anerkannte Ausbildung

☐ Meisterprüfung

☐ Hochschulstudium (Bachelor/Master/Diplom)

☐ Promotion

☐ sonstiges
