# Supplementary material for: Social factors associated with the development of e-health literacy: A mixed-methods study among patient organization members and representatives
Source: PLOS Digit Health. 2026 Aug 13;5(8):e0001569. doi: 10.1371/journal.pdig.0001569 (PMC13472428; doi:10.1371/journal.pdig.0001569)
Supplement: Supplementary file 2 [file pdig.0001569.s002.pdf]

Regeln für eine einheitliche Transkription: PANDORA Interviewstudie mit Mitgliedern

Die auf Tonband aufgezeichneten Experteninterviews sind alle komplett wortwörtlich zu transkribieren. Eine genaue, wortwörtliche Transkription hilft bei der späteren Auswertung, das Datenmaterial besser zu verstehen, sodass etwa unterschiedlich aufgefasste, widersprüchliche (z. B. Ironie) oder unvollständige Aussagen bewertet werden können. In das Transkript werden alle auf der Tonbandaufzeichnung hörbaren Zeichen aufgenommen: die verbalen, paraverbalen und die nicht verbalen sprachlichen Äußerungen der beteiligten Sprecher(innen), *insofern* sie Teil einer Antwort oder Frage sind oder eine eindeutige Bejahung oder Verneinung artikulieren. Situationsgebundene Geräusche brauchen nicht mittranskribiert zu werden.

1. Transkriptionsregeln

- Die Aufnahmen der Interviews, die mit Mitgliedern durchgeführt sind (zu erkennen an „M[Zahl]" als Dateiname), brauchen **erst ab der Frage „2)** Was bedeutet für Sie „Digitalisierung" in der medizinischen Forschung und Versorgung?", die vom Interviewer gestellt wird, **transkribiert werden**. Das heißt: Die Vorstellung der jeweiligen Person, in der personenbezogene Daten erzählt werden, kann übersprungen werden.
- Als **verbale Äußerungen** sind alle ganz oder teilweise ausgesprochenen Wörter und Wortfolgen zu verstehen.
- Besteht eine **Äußerung NUR aus „mhm"** ohne jegliche weitere Ausführung, wird es als „mhm (bejahend)", oder „mhm (verneinend)" erfasst, je nach Interpretation. In diesem Fall ist das Verständnissignal als (inhaltliche) Antwort zu verstehen. **AUSNAHME**: Das Verständnissignal 'mhm' des gerade nicht Sprechenden, es wird nicht transkribiert. Bestätigende Worte wie "genau", "ja", "okay" werden hingegen wie ein Inswortfallen in eckigen Klammern mittranskribiert, s. unten [ ])
- Ebenso werden die **nicht verbalen Äußerungen** im Transkript mit aufgenommen, wie zum Beispiel geräuschvolles lachen oder weinen (z.B. (lacht))
- Falls es in den Interviews zu **technischen Unterbrechungen** kommt, werden diese mit Kommentaren festgehalten (z.B. (Unterbrechung/Rauschen))
- Wenn mehrere Personen gleichzeitig sprechen bzw. eine Person unterbrochen und dazwischengeredet wird, sollen die Unterbrechungen mit Pseudonym versehen in eckige Klammern an der entsprechenden Stelle eingefügt werden.
- Die interviewende Person wird mit „I:" abgekürzt, die Interviewte Person mit dem Aliasnamen (M[zahl]).
- **Zahlen** werden nach Möglichkeit ausgeschrieben, Jahreszahlen hingegen als Ziffernfolge angegeben.
- **Lückenfüller** wie z.B. „ähm" werden weggelassen

- **Hinter** jeder Äußerung einer Person wird die Zeit markiert: „#00:11:35#“ (daher ist es notwendig, dass mit dem Programm f4 oder mit MAXQDA transkribiert wird).
- **Satzzeichen** werden verwendet, um der Leserin des Transkriptes zu helfen, den Originalsprachfluss zu rekonstruieren. Sie werden nicht entsprechend den üblichen Grammatikregeln gesetzt, da gesprochene Sprache meist Unregelmäßigkeiten aufweist. Als Satzzeichen werden verwendet:

2. Zeicheninventar

.	Abgeschlossener Gedanke, meist auf dem Grundton endende Stimmführung
,	kurzes Zögern, Absetzen, Gedanke wird fortgesetzt
.	Abgebrochener Gedanke wurde durch einen Punkt markiert
(…)	Sprechpausen bis zu drei Sekunden werden durch drei Punkte markiert. Wenn eine Pause entsprechend länger war, wurde die Pausenzeit in Klammern geschrieben (z. B. (5. Sek.)).
[ ]	Falls es während des Interviews vorkommt, dass sich Interviewer und Interviewte ins Wort fallen, wird dieses gekennzeichnet, indem die Person, die dem Redner ins Wort fällt, an dieser Stelle in eckigen Klammern mit dem Kommentar in den Redetext eingefügt wurde (z. B. [EW05: Das stimmt]).
#Zeitmarke# (erzeugt F4 automatisch durch Enter)	Jeder Sprecherbeitrag erhält eigene Absätze. Zwischen den Sprechern gibt es eine freie, leere Zeile. Auch kurze Einwürfe werden in einem separaten Absatz transkribiert. Beispiel:<br>B: Ich habe dort #Zeitmarke#<br>I: Wo genau? #Zeitmarke#<br>B: eingekauft. #Zeitmarke#
(unv.) #Zeitmarke#	Unverständliche Äußerung mit Zeitmarke
(Häuser?)	Vermuteter Wortlaut
Digit_	abgebrochenes oder unausgeführtes Wort (Wortabbrüche bzw. ein nicht zu Ende gesprochenes Wort ist im Transkript beizubehalten)
GROSSSCHREIBUNG	betonte Wörter oder Äußerungen werden durch GROSSSCHREIBUNG gekennzeichnet.

- Das Transkript wird als Word-Datei (docx-Datei) gespeichert. Die Benennung der Datei erfolgt entsprechend dem Mediendateinamen (ohne Endung), beispielsweise: M1.docx.

3. Regeln zum Pseudonymisieren. Gerne hierfür eine Tabelle mit den Pseudonymen anlegen:

1. Alle Namen von Patientenorganisationen sollten pseudonymisiert werden: (PO[Zahl]).
2. Alle genannten Krankheiten sollten pseudonymisiert werden: (K[Zahl]).
3. Alle genannten Medikamente sollten pseudonymisiert werden: (Mk[Zahl]).
4. Alle Eigennamen von Personen, die erwähnt werden, sollten pseudonymisiert werden: Hier gerne die Beziehung zur interviewten Person angeben, z. B. (Mutter/ Elternteil) oder (Ärztin), wenn dies aus dem Kontext klar wird.
