# Supplementary material for: Social factors associated with the development of e-health literacy: A mixed-methods study among patient organization members and representatives
Source: PLOS Digit Health. 2026 Aug 13;5(8):e0001569. doi: 10.1371/journal.pdig.0001569 (PMC13472428; doi:10.1371/journal.pdig.0001569)
Supplement: Supplementary file 3 [file pdig.0001569.s003.pdf]

Coding guide_Members of Patient Organisation (German original)

Kodierleitfaden Mitglieder zur Interviewstudie *„Digitalisierungsprojekte in der medizinischen Forschung und Versorgung“* (Stand 24.03.2023)

Dr. Henk Jasper van Gils-Schmidt, B. Sc.
Department Gesundheitwissenschaften, Fakultät Life Sciences
HAW Hamburg
Ulmenliet 20
21033 Hamburg

<u>Definitionen</u>

Digitalisierung: *Digitalisierung bezeichnet die Umwandlung von analogen Daten und Informationen in digitale Formate. Breiter gefasst beschreibt Digitalisierung auch die Umwandlung sozialer Praktiken, die analog bzw. in Person stattgefunden haben, in Formen, in denen diese Praktiken durch digitale Anwendungen vermittelt werden.*

Patientenregister: *Bei einem Patient*innenregister handelt es sich meist um ein (digitales, elektronisches) Datenbanksystem, worin die personengebundenen Daten von Patient*innen mit einer bestimmten Erkrankung erfasst werden. In einem solchen Register werden nicht nur Erkrankungsmerkmale, sondern auch darauf abgestimmte (medikamentöse) Behandlungsmethoden festgehalten*

Patientenorganisation: *Patient*innenorganisationen sind Interessevertretungen behinderter oder kranker Menschen sowie ihrer Angehörigen (z. B. Eltern) und setzen sich dafür ein, die Versorgung von Betroffenen zu verbessern und die Kompetenzen der Betroffenen im Umgang mit ihrer Erkrankung oder Behinderung zu stärken. Viele Patient*innenorganisationen engagieren sich zudem politisch, leisten national und international Vernetzungsarbeit, unterstützen medizinische Forschung und rufen eigene Forschungsprojekte ins Leben.*
*Patient*innenorganisationen sind in der Regel Zusammenschlüsse Betroffener oder/ und deren Angehöriger. Sie verfolgen nicht in erster Linie wirtschaftliche Ziele und sind zumeist gemeinnützige eingetragene Vereine.*

E-Health: *E-Health (kurz für Electronic Health), meint sinngemäß übersetzt „Gesundheit basierend auf elektronischer Datenverarbeitung“ und steht als Oberbegriff für die Digitalisierung im medizinischen Bereich. Mit E-Health wird diejenige Kategorie von medizinischen Anwendungen und Maßnahmen bezeichnet, die sich auf*

die digitale Unterstützung oder digitale Verlagerung von medizinischer Diagnostik, Behandlung und Versorgung richtet. E-Health-Produkte können von privatwirtschaftlichen Unternehmen, von Vereinen und Organisationen sowie von der öffentlichen Gesundheitsversorgung angeboten werden.
*Beispiele für solche Produkte sind: die elektronische Patient*innenakte, digitale Patient*innenregister, Telemedizin für die Kommunikation zwischen Ärztin/ Arzt und Patient*in, Online Apotheken, Apps auf Rezept, die unblutige Glucose Messung für Diabetiker oder der Notrufknopf für Senioren.*

M-Health: *M-Health (kurz für Mobile Health) lässt sich übersetzen als „mobile Gesundheit" und bezeichnet eine Untergruppe von E-Health-Aktivitäten und -Maßnahmen der Gesundheitsversorgung, die auf mobilen, drahtlosen Endgeräten, wie Smartphones oder Tablets, angeboten werden. Die Anwendungsbereiche sind, unter anderem, die Ermittlung von Vitalwerten wie den Blutzucker oder die Körpertemperatur (siehe Remote Monitoring) oder Motivationsanwendungen, die z. B. an die Einnahme von Medikamenten erinnern (siehe Selbstmanagement der Krankheit).*
Beispiele von M-Health-Produkten sind: Corona-Warn App, Anwendungen zur Überwachung von Vitalfunktionen (z. B. Blutdruckmessgeräten, Blutzuckermessgeräten), Wearables (z. B. Fitnessarmbänder).

Gesundheitskompetenz: *Gesundheitskompetenz umfasst das Wissen, die Motivation und die Fähigkeit von Menschen, relevante Gesundheitsinformationen zu finden, zu verstehen, zu beurteilen und im Alltag anzuwenden, um beispielsweise informierte gesundheitsrelevante Entscheidungen zu treffen. Gesundheitskompetenz bezeichnet zudem die Fähigkeit von Menschen, sich im System der Gesundheitsversorgung zu orientieren, geeignete Leistungen und Einrichtungen zu identifizieren und den eigenen Leistungsanspruch erfolgreich geltend machen zu können.*

Digitale Kompetenz: *umfasst das Wissen, die Motivation und die Fähigkeiten von Menschen, am digitalen Alltag teilzunehmen und mit digitalen Anwendungen umzugehen, mit ihnen zu lernen, zu arbeiten, und sie kritisch zu bewerten.*

Broad consent: *Die breite Einwilligung (englisch: broad consent) ist eine Art der Einwilligungserklärung, bei der Patient*innen zustimmen, dass ihre Daten in zukünftigen Forschungsprojekten verwendet werden dürfen, ohne dass eine erneute Einwilligung notwendig ist. Der Inhalt und Umfang dieser künftigen Forschungsprojekte sind dabei zum Zeitpunkt der Einwilligung noch nicht bekannt. Die breite Einwilligung soll es ermöglichen, bereits vorhandene Daten für neue Forschungszwecke wiederzuverwenden, ohne dafür viel organisatorischen Aufwand betreiben zu müssen. Die breite Einwilligung soll es außerdem den Personen, die ihre Einwilligung geben, erleichtern, zu Forschung beizutragen und verhindern, dass sie durch ständige neue Bitten um Einwilligung überlastet werden. Das Modell der breiten Einwilligung soll also Forscher*innen und Patient*innen entlasten und durch geringeren Verwaltungsaufwand Kosten einsparen. Kritisch angemerkt zum Modell der breiten Einwilligung wird, dass es die Autonomie der Patient*innen gefährden kann, deren Daten erhoben und verarbeitet werden.*

Fragestellungen

- Welche digitalen Angebote für die medizinische Forschung und/ oder Versorgung sind bei Mitgliedern von Patientenorganisationen bekannt? Wird ein Unterschied zwischen Angeboten im Bereich medizinischer Forschung und im Bereich medizinischer Versorgung wahrgenommen?
- Wie bewerten Mitglieder digitale Angebote? Ist die Bewertung im Rahmen der Forschung und der Versorgung unterschiedlich?
- Wie ist die Einschätzung davon, welche Aspekte der Gesundheitskompetenz und digitalen Kompetenz notwendig sind, um digitale Angebote effektiv nutzen zu können und selbstbestimmte Entscheidungen hinsichtlich solcher Angebote treffen zu können?
- Welche Verantwortungsbereiche in der Entwicklung und Nutzung von digitalen Angeboten werden wahrgenommen? Und wer sind die Akteure, die diese Verantwortung erfüllen müssen?

- Welche Einstellungen haben Mitglieder zu dem digitalen Patientenregister der eigenen Patientenorganisation?
- Welche Gründe für und gegen die Teilnahme am Register werden expliziert?
- Welche Maßnahmen liegen im Verantwortungsbereich der Patientenorganisation, die das Register (mit-)initiiert hat?

- Wie bewerten PO-Mitglieder ein Broad Consent im Rahmen von Forschungsstudien bewertet?

	Code	Beschreibung/Bedeutung, inkl. Anwendung des Codes	Nicht zu verwechseln mit	Ankerbeispiel
Digitalisierung Allgemein				
1.	Kenntnis_DigTech	Benennung oder Beschreibung von digitalen Technologien im Rahmen der medizinischen Forschung oder Versorgung. Das bedeutet, die interviewte Person benennt oder beschreibt eine digitale Technologie, wie:<br>1. PR (Patientenregister)<br>2. AI (Artifical Intelligence-Anwendungen/ KI)<br>3. BG (Big Data)<br>4. TM (Telemedizin)<br>5. eRezept<br>6. ePA (Elektronische Patientenakte)<br>7. DiGA (Digitale Gesundheitsanwendungen)<br>8. M-Health (Gesundheitsapps die keine DiGA sind oder Gesundheitstracker)<br><br>***Bemerkung**: Dieser Code kann später im Prozess, aufgrund des Lesens der Transkripte (= induktiv), ggf. nach spezifischen Technologien in Subcodes unterteilt werden. Dies würde es ermöglichen, bei späteren Codes die Antworten nach Technologien zu analysieren (z.B. Grad der Gesundheitskompetenz, Code 6).*		*Beispiele für Benennung:*<br>„Also ich denke an **Register**, da kommen wir später noch drauf, […]“ [redacted]<br><br>„[…] also meine **elektronische Patientenakte** das ist mir immer noch zu kompliziert. [I: Okay.] Ich nutze auch keine **DiGA** weil ich einfach nicht die Gelegenheit habe […]“ [redacted]<br><br>*Beispiel für Beschreibung (Telemedizin):*<br>„Ich habe davon gehört ja (…) konkret (…) ja gibt es glaub ich **Videosprechstunden** die die Hausärzte schon mal anbieten (…) ich glaub in der in der Psychotherapie ist das oder wird verbreitet dass man auch da **Telemedizinsprechstunden anbietet** […]“ [redacted]
2.	Einstellung_Dig	Es werden Einstellungen speziell zur Digitalisierung in der medizinischen Forschung und/ oder Versorgung oder zu der Bedeutung von dieser Digitalisierung beschrieben. Das heißt, dieser Code hat ein		

	Code	Beschreibung/Bedeutung, inkl. Anwendung des Codes	Nicht zu verwechseln mit	Ankerbeispiel
		Bewertungs- oder evaluatives Moment, welches sowohl pro als auch contra sein kann.<br><br>Sie wird nicht angewendet, wenn *nur* über eigene Erfahrungen berichtet oder auf digitale Produkte eingegangen wird.<br><br>**Nur die Subcodes dieses Codes werden angewendet.** *(**Bemerkung:** Dieser Hauptcode kann in der Analyse mit den Subcodes zusammengefasst werden.)*<br><br>***Bemerkung**: Dieser Code kann später im Prozess, aufgrund des Lesens der Transkripte (= induktiv), ggf. nach dem Unterschied „Forschung“ und „Versorgung in weiteren Subcodes unterteilt werden.*<br><br>***Bemerkung:** In der Kodierung bzw. Analyse sollte darauf geachtet werden, ob eine klare Trennung zwischen* Anliegen (z.B. Wert von Privatheit) *und* Motiven (z.B. Solidarität als Motivation) *gemacht werden kann. Dies sollte ggf. dann zusätzlich kodiert werden.*		
2.1	Einstellung_Dig_Pro	Dieser Subcode wird angewendet, wenn die beschriebenen Einstellungen zur Digitalisierung <u>im medizinischen Bereich allgemein</u> **positiv** sind (auch , wenn unklar ist, ob Forschung oder Versorgung spezifisch gemeint ist).	Erfahrung_Dig_selbst UND Einstellung_Dig_Con	„Na ja also ich weiß nicht wie weit Sie jetzt (...) die Behandlung (...) über Telemedizin dazurechnen. **Also für mich scheint das eine sehr gute Möglichkeit zu sein und führt eben dazu dass Patienten und Ärzte über große Entfernung auch zusammengeschlossen werden können.“** [redacted]<br><br>„[...] also gerade bei der elektronischen Patientenakte kann ich es ja eigentlich selber entscheiden was was was draufkommt oder nicht

	Code	Beschreibung/Bedeutung, inkl. Anwendung des Codes	Nicht zu verwechseln mit	Ankerbeispiel
				und was ich freigebe oder nicht (...) und (5 Sek.) **also für mich im Moment seh ich da eigentlich nur nur Chancen** […]“ [redacted]<br><br>„[…] **ich denke auch die DiGAs sind auf die Dauer eine ganz gute Sache**, also die diese digitalen Anwendungen mit denen man selber seine Krankheit steuern kann, sie bringen halt auch neue Anforderungen aber ich glaube in im Prinzip ist es eine ganz gute Sache […]“ [redacted]
2.2	Einstellung_Dig_Con	Dieser Subcode wird verwendet, wenn die beschriebenen Einstellungen zur Digitalisierung **negativ** sind (auch wenn unklar ist, ob Forschung oder Versorgung spezifisch gemeint ist).	Erfahrung_Dig_selbst UND Einstellung_Dig_Pro	„Also ich **bin im Augenblick noch nicht von dem Nutzen überzeugt für mich persönlich jetzt**, denn ich sammel meine Daten noch also meine meine Unterlagen auch ganz gerne in Papier das reicht für mich im Augenblick völlig aus. Ich kann mir vorstellen dass wenn die gut ausgebaut ist dass man DA wirklich einen Nutzen hat weil man die Ergebnisse überall mit sich rumträgt aber da muss ich auch ganz ehrlich sagen ich bin mir gar nicht sicher ob ich IMMER will dass ALLE meine Daten dann dadurch erreichbar also einsehbar sind.“ [redacted]<br><br>„[…] wir haben auch schon mal drüber nachgedacht ob die **Entwicklung der Telemedizin unter Umständen auch die Gefahr birgt** dass dann die Krankenhäuser eben das als Vorwand nehmen um die wirkliche Versorgung wo ja so ein ein Arzt auch physisch zur Verfügung (lacht) steht und und sich Zeit nimmt für den Patienten um das runterzufahren um da Kosten zu sparen weil sie sagen ja wir haben **ja Telemedizin also natürlich muss man immer aufpassen wie wirkt sich eine Änderung aus und was bedeutet das für die Versorgung des Patienten** […]“ [redacted]

	Code	Beschreibung/Bedeutung, inkl. Anwendung des Codes	Nicht zu verwechseln mit	Ankerbeispiel
				„Also eine (...) ich weiß dass die Patientenvertretung auch über die (PO[2]) dass die Patientenvertretung in der Regel ganz große Sorge haben vor Datenlecks vor, davor dass Daten in die falschen Hände geraten eben zur Manipulation genutzt werden oder um Patienten ganze Patientengruppen auszugrenzen, also wenn zum Beispiel im im Versicherungsbereich zum Beispiel, und diese Risiken seh ich natürlich auch. (...) **Also man hört ja immer wieder über Datenlecks** dass die KBV (lacht) plötzlich ein riesiges Datenleck hat und so weiter und **dass diese Daten in falsche Hände geraten und das ist natürlich ein Risiko.**“ [redacted]
3.	Erfahrung_Dig_selbst	Persönliche Erfahrungen, welche die interviewte Person bereits mit digitalen Anwendungen bzw. ihrem Gebrauch gemacht hat. Es sind konkrete Beispiele von der Nutzung digitaler Angebote gemeint.<br><br>Dieser Code wird angewendet, wenn auf eigene Erfahrungen eingegangen wird. Ist hierbei ein Bewertungs- bzw. evaluatives Moment inkludiert, dann muss auch mit einer Subcode von „Einstellung_Dig“ kodiert werden.	Einstellung_Dig<br>UND<br>Erfahrung_Dig_fremd	„[...] für mich persönlich ist eben auch sehr relevant die neuen Möglichkeiten über Apps und Smartwatches und so dass man seine eigenen Bewegungsprofile **zum Beispiel also beim Laufen zeichne ich meine Läufe auf und und dabei auch die Herzrate und kann mich dabei selbst überwachen und aber auch den Fortschritt erkennen und auch das dann auf sozialen Medien (lacht) teilen** natürlich also anderen mitteilen was man gemacht hat was zur Motivation beiträgt sich gegenseitig anzuspornen also das ist auch weitestgehend ein Teil der der Medizin ne für mich da weil ich eben laufe um meine Lunge zu trainieren (...) dadurch gibt es ja Verbindungen zu den zu den Trainingseinheiten.“ [redacted]<br><br>*Beispiel für die Doppelkodierung mit Einstellung_Dig (Einstellung ist gelb unterlegt zur Hervorhebung):*<br>„Das ist (...) ja nutzen würd ich gerne aber ich habe das Gefühl nach allem was ich bis jetzt festgestellt hab dass es nur Wort aber das funktioniert nicht ansatzweise. [I: Okay.] (...) Also ich ich ich hatte das tatsächlich vor elekt_ oder **ich hab mich auch angemeldet (...) die elektronische Patientenakte zu nutzen**, wie ich gesagt hab momentan **hab ich so Diagnosen oder Befundberichte wenn ich**

	Code	Beschreibung/Bedeutung, inkl. Anwendung des Codes	Nicht zu verwechseln mit	Ankerbeispiel
				**welche bekomme auf dem Smartphone und oder Tablet abgespeichert** und ich hab mir vorgestellt dass man das auch auf dem Chip der Krankenkassenkarte **speichern** kann. Aber nach allem was ich jetzt lese bei meiner Krankenkasse funktioniert das **überhaupt nicht.**“ [redacted]
4.	Erfahrung_Dig_fremd	Erfahrungen, welche die interviewte Person indirekt über Angehörige mit digitalen Anwendungen bzw. ihrem Gebrauch gemacht hat. Es sind konkrete Beispiele von der Nutzung digitaler Angebote gemeint.<br><br>Ist hierbei ein Bewertungs- bzw. evaluatives Moment inkludiert, dann muss auch mit einer Subcode von „Einstellung_Dig“ kodiert werden.	Erfahrung_Dig_selbst	„Ja gut **die meisten Leute (räuspert sich) kennen sich ja schon ein bisschen mit Computer und Internet aus** (...) das ist halt ja (...) hm [I: Ja.] (...) ich also hm in meinem Privatumfeld, wieder die zwei Extreme ich die halt beruflich damit zu tun hat (...) und **da viel mehr ausprobiert und viel mehr guckt und auch weiß worauf sie ein bisschen achten muss** [...]“ [redacted]<br><br>*Beispiel für die Doppelkodierung mit Einstellung_Dig (Einstellung ist gelb unterlegt zur Hervorhebung):*<br>„[...] also zumindest bei den Leuten die ich kenne sind natürlich alles ältere, also sind **sehr viele ältere dabei, und die viel weniger Kontakt mit digitalen mit mit digitalen Prozessen habe** als ich jetzt die sind da einfach sehr sehr verunsichert.“ [redacted]
Digitale Kompetenz & Gesundheitskompetenz				
5	GK_Aspekte	Es wird über das eigene Verständnis der Gesundheitskompetenz und/ oder die Aspekte, woraus die Gesundheitskompetenz besteht, berichtet. Entweder können diese Aspekte direkt benannt werden oder sie können anhand von Alltagsbeispielen illustriert werden. Beispiele sind, dass die Person bestimmtes Gesundheitswissen, Motivationen oder Fähigkeiten von Patient*innen beschreibt.	dK_Aspekte UND GK_AllgVoraussetzungen	„Na ja ich würde als **Gesundheitskompetenz seitens des Patienten** beschreiben [I: Ja.] dass ich die (...) **gesundheitlichen, Fragen die ich habe angemessen beantworten oder lösen kann.** Das heißt dass ich **in der Lage bin zu wissen wo frage ich nach wo hole ich mir Informationen also gültige Informationen wo finde ich Informationen wie baue ich die ein wo will ich Informationen haben und wo will ich keine Informationen haben.** (...) Also dass ich **bewusste Entscheidungen über meine Gesundheit** fällen kann [...]“ [redacted]

	Code	Beschreibung/Bedeutung, inkl. Anwendung des Codes	Nicht zu verwechseln mit	Ankerbeispiel
		Hierbei muss das Verständnis der Gesundheitskompetenz nicht im Zusammenhang mit der Digitalisierung im medizinischen Bereich oder E-Health Angeboten stehen, dies ist aber möglich.		„Na ich denk das ist einfach **die persönliche Einstellung.** (...) Also ich ich bin jetzt jemand ich will immer alles wissen ne **also ich frag ja dann auch Dinge nicht unbedingt jetzt mit dem Befund zu tun haben** und dann fällt mir wieder irgendwas ein wie wie geht denn das und das was ist denn da und da **ich will immer alles genau wissen** ich will auch immer wissen wenn ich operiert werde wie genau funktioniert die OP. (lacht) (...) Am besten noch mit Video und so ein Zeug [...] " [redacted]
6	GK_AllgVoraussetzungen	Die interviewte Person benennt oder beschreibt die allgemeinen Voraussetzungen, die Patient*innen benötigen, um sich an Gesundheitsprojekten beteiligen bzw. Gesundheitsanwendungen nutzen zu können.<br><br>Dieser Code beschreibt entweder die Voraussetzungen oder Bedingungen, die für eine gute Beteiligung im Gesundheitssystem notwendig sind, oder eine Stufe oder einen Grad an Gesundheitskompetenz.<br><br>***Ausnahme**: Wenn die Voraussetzungen für die Gesundheitskompetenz oder der Grad der Gesundheitskompetenz gleichzeitig mit der und digitalen Kompetenz angesprochen wird, sind beide Codes zuzuweisen.*	GK_Aspekte UND dK_AllgVoraussetzungen<br><br>[Ausnahme: siehe Beschreibung]	„[...] abhängig von der vom Schweregrad der Erkrankung also die (K[1]) ist per se tödlich und die Therapiemaßnahmen sind unglaublich zeitaufwendig ich hab also bis zu vier Stunden früher Atemtherapie machen müssen am Tag und **je mehr ich davon verstehe und je mehr ich das optimiere umso wirksamer ist** und umso mehr kann ich diesen Zeitaufwand begrenzen und nur so ist es dann möglich nebenher noch zu arbeiten und (lacht) andere Dinge zu machen also von daher [I: Ja.] würd ich sagen **immer das Höchstmögliche Bestmögliche die bestmögliche Kompetenz erreichen** [...] [redacted]
7	dK_Aspekte	Es wird über das eigene Verständnis der digitalen Kompetenz und/ oder die Aspekte, woraus diese besteht, berichtet. Entweder können diese Aspekte direkt benannt werden oder sie können anhand von Alltagsbeispielen illustriert werden. Beispiele sind,	GK_Aspekte UND dK_AllgVoraussetzungen	„Also die die **Fähigkeit zu verstehen wie ich zum Beispiel etwas auflade wie ich mich identifiziere und authentifiziere im Netz dann das Gadget oder das, die die Anwendungen zum Laufen zu bringen dann die Anwendung zu verstehen was was sie also tun muss,**

	Code	Beschreibung/Bedeutung, inkl. Anwendung des Codes	Nicht zu verwechseln mit	Ankerbeispiel
		dass die Person bestimmtes Wissen über digitale Technologien, Motivationen oder Fähigkeiten von Patient*innen beschreibt.<br><br>Hierbei muss das Verständnis der digitalen Kompetenz nicht im Zusammenhang mit der Digitalisierung im medizinischen Bereich oder E-Health Angeboten stehen, dies ist aber möglich.		**eventuell die die Grafiken die mir gezeigt werden zu verstehen** […]“ [redacted]
8	dK_AllgVoraussetzungen	Die interviewte Person benennt oder beschreibt die allgemeinen Voraussetzungen qua technologischer Mittel oder digitaler Fähigkeiten, die Patient*innen benötigen, um sich an digitalen Gesundheitsprojekten beteiligen bzw. digitale Anwendungen nutzen zu können.<br><br>Dieser Code beschreibt entweder die Voraussetzungen bzw. Bedingungen, die für eine Beteiligung an digitalen Technologien im Gesundheitssystem notwendig sind, oder eine Stufe oder einer Grad an digitaler Kompetenz, die benötigt wird, um sich an digitalen Gesundheitsprojekten beteiligen zu können.<br>Um diesen Code zuzuordnen muss ein Zusammenhang zu E-Health und digitalen Gesundheitsprojekten bestehen.<br><br>***Ausnahme****: Wenn die Voraussetzungen für die digitalen Kompetenz oder der Grad der digitalen Kompetenz gleichzeitig mit der*	dK_Aspekte UND GK_AllgVoraussetzungen<br><br>[Ausnahme: siehe Beschreibung]	„Na ja es gehört einfach **grundsätzliches Verständnis für IT-Prozesse** würd ich sagen.“ [redacted]<br><br>„[…] natürlich ist so ein **bisschen digitales Grundverständnis notwendig** […]“ [redacted]<br><br>*Beispiel für Doppelcodierung:*<br>„Also dann er muss **zumindest verstehen wie es funktioniert** also am Beispiel des Zuckersensors der muss ja den Sensor an dem iPhone oder an dem Telefon anmelden können der muss erkennen können wenn irgendwie ein Fehler entsteht und und die Werte die da angezeigt werden vielleicht nicht stimmen er muss also Bescheid wissen was was sind normal wie sind die Werte einzuschätzen was sind die Konsequenzen wenn ich einen bestimmten Wert angezeigt bekomme und (…) also digitale Kompetenz ist dann eben auch die die Auswertungen an den Arzt schicken zu können sich an- und abmelden zu können Login was weiß ich **diese ganzen technischen Dinge muss er soweit beherrschen dass er mit gutem Gefühl diese Anwendungen benutzen kann.**“ [redacted]

	Code	Beschreibung/Bedeutung, inkl. Anwendung des Codes	Nicht zu verwechseln mit	Ankerbeispiel
		Gesundheitskompetenz angesprochen wird, sind beide Codes zuzuweisen.		
Verantwortung				
9.1-6	Verantwortung _[Akteurkürzel]	Akteure, von denen die befragte Person angibt, dass sie eine Verantwortung oder Zuständigkeit haben, Maßnahmen zu ergreifen, um Patient*innen, die digitale Angebote nutzen, zu schützen (z.B. Datenschutzvorgaben) oder zu unterstützen (Förderung von Kompetenzen im Umgang mit digitalen Technologien).<br><br>Wenn die Verantwortungsbereiche bzw. -aspekte, die für die einzelnen Akteure identifiziert werden, genannt werden, werden diese direkt mitkodiert.<br><br>Hier ist zu unterscheiden zwischen:<br>1. Politik (Behörde; Gesetzgebung; etc.)<br>2. PO (Patient*innenorganisationen)<br>3. GS (Akteure aus dem Gesundheitssystem, wie Krankenhäuser, Ärtz*innen, etc.)<br>4. Hersteller von digitalen Produkten<br>5. Nutzer (Patient*innen, etc.)<br>6. Sonstiges		***Bemerkung**: Hier unter ist mit Farben kodiert, um zu zeigen, wie Subcodes aufeinanderfolgen können. In MAXQDA muss diese Textpassage aber getrennt nach den Subcodes kodiert werden. Beispiel für 9.1, 9.3, 9.5 und 9.4:*<br>„[…] Also bei der EVA (lacht) hat im Prinzip (räuspert sich) die Politik erst mal die Verantwortung das Ganze zu initiieren, (…) dann natürlich ich muss ehrlich sagen ich weiß jetzt nicht genau wie wie dieses Gremium sich nennt dass die EPA ja entwickeln und voranbringen soll, dann liegt da die Verantwortung bei den Beteiligten das Projekt auch in einer angemessen Form durchzuführen, dann läge es beim Patienten die Entscheidung zu treffen ja oder nein also will ich die, mir die EPA runterladen will ich sie verwenden oder nicht, jedenfalls für einen gewissen Zeitraum noch, und dann liegt sie bei den Ärzten eben auf eine korrekte Art oder angemessene Art mit dieser EPA umzugehen.“ [redacted]<br><br>***Bemerkung**: Hier unter ist mit Farben kodiert, um zu zeigen, wie Subcodes aufeinanderfolgen können. In MAXQDA muss diese Textpassage aber getrennt nach den Subcodes kodiert werden. Beispiel für 9.1, 9.3 und 9.5:*<br>„Also, (…) da ich überzeugt bin dass Patientenregister den Fortschritt der Therapie voranbringen können und deswegen für die Patienten wichtig sind und natürlich auch für den einzelnen Patienten Service bieten der nützlich ist um mit der Erkrankung klarzukommen fände ich schon dass das Gesundheitswesen als Ganzes die Aufgabe hätte solche Patientenregister zu finanzieren

	Code	Beschreibung/Bedeutung, inkl. Anwendung des Codes	Nicht zu verwechseln mit	Ankerbeispiel
				und damit das ist ein die die Finanzierungsverantwortung sozusagen (lacht) ne und das ist dann letztendlich Aufgabe der Politik da Vorgaben zu machen (…) aber die trotzdem, ist sind es nicht die einzigen die Verantwortung tragen also auch die Ärzte und Behandler tragen natürlich Verantwortung dass sie Daten korrekt eingeben dass sie sich engagieren und die Zeit aufwenden die Daten überhaupt zu erzeugen dass sie sie auch mit dem Patienten besprechen und die Ergebnisse des Registers nutzen da haben wir auch ganz unterschiedliche Erfahrungen also es gibt bei uns Ambulanzen die da die jedes Feature des Registers nutzen und sofort den Patienten zugänglich machen und bei anderen wird läuft es so nebenher und wird eher lieblos gehandhabt [I: Okay.] also (…) und eben die Verantwortung des Patienten seine Daten herzugeben sonst funktioniert es ja auch nicht.“ [redacted]
Das digitale Patientenregister				
10	Einstellung_dPR_selbst **[Nur die Subcodes beim Kodieren verwenden]**	Es werden Einstellungen und Erfahrungen beschrieben, die die Person selbst zu dem digitalen Patientenregister der eigenen Patient*innenorganisation hat. Diese Einstellungen inkludieren auch die Vor- und Nachteile, die die interviewte Person wahrnimmt.<br><br>Sie wird nicht angewendet, wenn *nur* über eigene Erfahrungen bzgl. Des Registers berichtet wird.<br><br>**Nur die Subcodes dieses Codes werden angewendet.** *(**Bemerkung:** Dieser Hauptcode kann in der Analyse mit den Subcodes zusammengefasst werden.)*		

	Code	Beschreibung/Bedeutung, inkl. Anwendung des Codes	Nicht zu verwechseln mit	Ankerbeispiel
		***Bemerkung:** In der Kodierung bzw. Analyse sollte darauf geachtet werden, ob eine klare Trennung zwischen* Anliegen *und* Motiven *gemacht werden kann. Dies sollte ggf. dann zusätzlich kodiert werden.*		
10.1	Einstellung_dPR_selbst_Pro	Dieser Subcode wird verwendet, wenn die beschriebenen Einstellungen und Erfahrungen, die die Person selbst hat, dem digitalen Patient*innenregister **positiv** gegenüberstehen oder wenn **Vorteile** des Registers benannt werden, die die Person selbst wahrnimmt.	Einstellung_dPR_fremd	„[...] aber **mir von war von Anfang an klar dass das Register eine ganz wichtige Einrichtung ist oder ein ganz wichtiges Mittel ist um (...) verifizierte Daten von Patienten zu dokumentieren und auszuwerten und damit den Verlauf der Erkrankung zu beschreiben.** [I: Ja.] (...) Und die Wirksamkeit von Maßnahmen zu beschreiben.“ [redacted]
10.2	Einstellung_dPR_selbst_Con	Dieser Subcode wird verwendet, wenn die beschriebenen Einstellungen und Erfahrungen, die die Person selbst hat, dem digitalen Patient*innenregister **negativ** gegenüberstehen oder wenn **Nachteile** des Registers benannt werden, die die Person selbst wahrnimmt.	Einstellung_dPR_fremd	„Also zunächst mal (...) da kommt man komm ich jetzt natürlich irgendwie aufs Register (lacht) weil also Verarbeiten heißt eben Speichern und Auswerten und die Auswertungen die betreffen eben erst mal mich also wenn ich meinen Arzt dann be_ wenn ich mit meinem Arzt gemeinsam die die Zuckerwerte anschaue oder die Lungenfunktionswerte oder auch die langfristige Entwicklung dieser ganzen Messungen aber richtige medizinische Forschung ist natürlich dann wenn man von vielen Patienten die Daten untersucht und verknüpft und (...) ja, **da gibt es natürlich auch verschiedene Risiken die man sich denken kann dass dann zum Beispiel irgendwie falsche Schlussfolgerungen gezogen werden oder dass pauschale (räuspert sich) Ergebnisse umgesetzt werden für alle Patienten obwohl die nicht für alle zutreffen** also jeder Patient ist ja individuell und hat seine eigenen Probleme [...] [redacted]
11	Einstellung_dPR_fremd **[Nur die Subcodes beim**	Es werden Einstellungen und Erfahrungen beschrieben, von denen die Person annimmt, dass sie für Fremde (andere Personen) einen Wert haben. Außerdem werden die Vor- und Nachteile		

	Code	Beschreibung/Bedeutung, inkl. Anwendung des Codes	Nicht zu verwechseln mit	Ankerbeispiel
	**Kodieren verwenden]**	beschrieben, von denen die interviewte Person annimmt, dass andere sie als relevant empfinden könnten.<br><br>Sie wird nicht verwendet, wenn *nur* über eigene Erfahrungen bzgl. des Registers berichtet wird.<br><br>**Nur die Subcodes dieses Codes werden angewendet.** *(**Bemerkung:** Dieser Hauptcode kann in der Analyse mit den Subcode zusammengefasst werden.)*<br><br>***Bemerkung:*** *In der Kodierung bzw. Analyse sollte darauf geachtet werden, ob eine klare Trennung zwischen* Anliegen *und* Motiven *gemacht werden kann. Dies sollte ggf. dann zusätzlich kodiert werden.*		
11.1	Einstellung_dPR_fremd_Pro	Dieser Subcode wird verwendet, wenn die beschriebenen Einstellungen und Erfahrungen, die die Person <u>anderen zuschreibt</u>, dem digitalen Patient*innenregister **positiv** gegenüberstehen oder wenn **Vorteile** des Registers genannt werden, die andere wahrnehmen könnten.	Einstellung_dPR_selbst	„Mhm (bejahend) also nach unserer Erfahrung ist die Zustimmungsrate da sehr hoch es gibt ganz wenige die das ablehnen von daher vermute ich dass **die allermeisten eben diese Chancen sehen** wie wir ja auch eine (räuspert sich) in unseren Kreisen eine extrem hohe Bereitschaft sehen an überhaupt an Studien teilzunehmen (...) (M5, #00:42:36#) [...] und (lacht) so also da ist immer **große Bereitschaft weil man eben dazu beitragen will den Thera_ Therapiefortschritte zu ermöglichen ja (...)**“ [redacted]
11.2	Einstellung_dPR_fremd_Con	Dieser Subcode wird verwendet, wenn die beschriebenen Einstellungen und Erfahrungen, die die Person <u>anderen zuschreibt</u>, dem digitalen Patient*innenregister **negativ** gegenüberstehen oder wenn **Nachteile** des Registers genannt werden, die anderen wahrnehmen könnten.	Einstellung_dPR_selbst	**„Also ich könnt mir vorstellen dass vielleicht jemand die Zustimmung nicht gibt (...) ich mein vielleicht nicht sicher sind was genau jetzt wirklich mit den Daten passiert.** (4 Sek.) Oder ob die vielleicht nicht, dann vielleicht noch **außerhalb Forschung oder so was noch, weiß ich nicht für irgendwas anderes noch benutzt werden** [...]“ [redacted]

	Code	Beschreibung/Bedeutung, inkl. Anwendung des Codes	Nicht zu verwechseln mit	Ankerbeispiel
				**„(…) und Risiken wenn jemand misstrauisch ist entweder der Institution gegenüber es gibt natürlich auch ein paar Leute die die wir vielleicht als Verein enttäuscht haben und die uns negativ gegenüberstehen** oder dem Arzt auch dem muss man natürlich vertrauen dass der da die richtigen Daten eingibt dass er die richtigen Schlussfolgerungen zieht dass er das mit mir bespricht **wenn da das Verhältnis gestört ist kann das dazu beitragen oder es gibt auch ganz individuelle Gründe die man, nicht unbedingt nachvollziehen muss (lacht) können muss** […]“ [redacted]
12	InfoAustausch	Die interviewte Person beschreibt, wie sie über das Patientenregister informiert worden ist.		„Da gibt es einen Informationsbogen der irgendwie vier Seiten lang oder sechs Seiten (…) wo eben alle wichtigen Dinge drauf stehen und dann aber auch Links vorhanden sind wo man dann das Datenschutzkonzept einsehen kann und das Konzept insgesamt für das Register und auch die die Berichte wir haben ja die Registerberichte die werden bei uns einmal für Wissenschaftler und Ärzte geschrieben und es gibt parallel dazu einen Bericht für Patienten der also lesbarer ist (lacht) sag ich mal [I: Okay. (lacht)] und den wir aber auch **unter den Mitgliedern eben immer dann bewerben und über Social Media darauf hinweisen** dass der wieder erschienen ist sodass viele Patienten eben bevor sie sich entscheiden schon davon wissen und auch auch von dem Nutzen wissen also ich ja aber auf Ihre Frage **also ungefähr sechs Seiten Informationen gibt es mit Links ins Internet für für weitere Informationen und mit Verweis auf diese externe Stelle wo man dann auch noch Fragen stellen kann oder [I: Okay.] sich beschweren kann (unv.)“** [redacted]<br><br>**„Da muss ich sagen dass ich mich auch nicht mehr dran erinnere** [I: Okay.] also ich weiß auch nicht mehr wann das war (…) und ich

	Code	Beschreibung/Bedeutung, inkl. Anwendung des Codes	Nicht zu verwechseln mit	Ankerbeispiel
				erinner mich, ich erinner mich einfach gar nicht mehr so richtig dran. [I: Und.] (...) Also ich gla_ also ich wei_ noch ich musste irgendwas unterschreiben und (...) ja also auf dem Zettel stand auch da für was es benutzt aber, an konkrete Sachen kann ich mich nicht mehr erinnern.“ [redacted]
12.1	InfoAustausch_ Wunsch	Die interviewte Person beschreibt, welche Informationen sie über zusätzlich zu den bereits erhaltenen Informationen gerne bekommen hätte.		„[...] aber **es macht vielleicht auch Sinn eine kurze schriftliche Broschüre** dazu zusammenzustellen wobei das immer so eine Sache ist mit ja evidenzbasierten Broschüren [...]“ [redacted]
13	Maßnahme_ PO	Die interviewte Person beschreibt Maßnahmen für die Entwicklung, Implementierung und Führung bzw. Unterhaltung eines Patientenregisters. Diese Maßnahmen können Maßnahmen sein, die tatsächlich von einer Patientenorganisation genommen werden, oder auch Maßnahmen, die die Person sich wünscht, dass eine Patientenorganisation diese ergreift oder entwirft.		„Also das **das Register muss wissenschaftlich gut begründet** sein und dann auch **gut durchgeführt** werden die wie wie bei Studien die Ansätze müssen von der Annahme her erst mal richtig sein korrekt sein. (...) **Gut der Datenschutz muss muss da sein.** (...) Ja, [I: Ja.] (...) also (...) das sind Selbstverständlichkeiten [I: Ja.] eigentlich.“ [redacted]
Broad Consent/ Breite Einwilligung/ Weite Zustimmung				
14	BC_Pro	Die interviewte Person beschreibt, welche Chancen und Vorteile sie in der Einführung eines Broad Consent für Forschungsstudien und den Umgang mit Daten sieht.<br><br>***Bemerkung**: Bei diesem Code ist keine Eigen- oder Fremdzuschreibung angedacht, da diese Frage oft eher kurz besprochen wurde. Falls dies in der Kodierung doch wichtig erscheint, kann diese Unterscheidung hinzugefügt werden.*		„[...] Also, ich persönlich hab damit kein Problem weil ich ja weiß dass die Daten anonymisiert pseudonymisiert verarbeitet werden und vor allen Dingen auch private Nutzer also Pharmaunternehmen nur aggregierte Daten erhalten also da werden keine Daten weitergegeben wo irgendwelche Rückschlüsse auf mich möglich sind und deswegen seh **ich das als unproblematisch an wenn ein Forschungsprojekt von den Gremien des Vereins als als förderungswürdig angesehen wird dort dort auch meine Daten zu verwenden** [...]“ [redacted]
15	BC_Con	Die interviewte Person beschreibt, welche Risiken und Nachteile sie in der Einführung eines Broad		„Aber dann **kann ich einfach zu wenig durchschauen was da passiert** also wenn man mir sagt ich möchte deine Daten für das und das Projekt verwenden und ich binde dann auch den

	Code	Beschreibung/Bedeutung, inkl. Anwendung des Codes	Nicht zu verwechseln mit	Ankerbeispiel
		Consent für Forschungsstudien und den Umgang mit Daten sieht.<br><br>***Bemerkung**: Bei diesem Code ist keine Eigen- oder Fremdzuschreibung angedacht, da diese Frage oft eher kurz besprochen wurde. Falls dies in der Kodierung doch wichtig erscheint, kann diese Unterscheidung hinzugefügt werden.*		**außereuropäischen Datenraum mit ein mit dem und dem Ziel okay aber jetzt zuzusagen in alle Ewigkeit freizugeben mit unbekanntem Ziel** da hab ich tatsächlich auch noch Hemmungen einfach [I: Okay.] weil ich zu wenig weiß was passiert da also **da fehlen mir tatsächlich Informationen um da eine gute eine informierte Entscheidung treffen zu können.**" [redacted]
16	BC_ Maßnahmen	Die interviewte Person beschreibt, welche Maßnahmen das Vertrauen bei der Abgabe eines Broad Consent erhöhen würden bzw. unter welchen Bedingungen sie ein Broad Consent abgeben würde.		„Generell ist es schon so dass man sich natürlich WÜNSCHEN würde so eine (...) also **statt einer breiten Einwilligung eine zu machen bei der man dann später noch einzelne Entscheidungen treffen kann** also das haben wir auch im im deutschen Ethikrat diskutiert bei der Big Data Stellungnahme dass man so eine also auch eine **treuhänderische Datenfreigabe** ne man könnte sich vorstellen dass es ein ein Patientengremium gibt mit mit Patientenvertretern dieser Erkrankung die dann so ein Forschungsprojekt sich anschauen und denen man den Auftrag gibt schaut euch das an und wenn ihr einverstanden seid dann bin ich auch dann geb ich meine Daten dafür auch frei **oder eben das das Optimum wäre bei jedem Forschungsprojekt werde ich kontaktiert und werd gefragt ob ich einverstanden bin** [...]" [redacted]
17	Sonstiges	Dieser Code gerne anwenden für die Textstellen, die von der kodierenden Person als relevant für die Forschungsfragen (siehe oben) erfahren werden, die aber zu keiner der oberen Codes passen. Hieraus können im Laufe des Kodierungsverfahren ggf. neue Codes gebildet werden.		*Finanzierung und langfristiger Unterhaltung eines Registers:*<br>„Also es gibt ja für die für den Betrieb von Registern einen Standard sozusagen also da müssen die Daten in einem anderen Server sein als die personenbezogen Daten und das muss eben die Einwilligung und die Aufklärung stattfinden und die muss auch regelmäßig erneuert werden und derjenige der der Sponsor ist also der es finanziert der ist darf nicht der sein der in die Daten guckt und so weiter und (...) diese, Regeln, müssen eingehalten werden und **das Problem was wir überwiegend sehen ist eben dass das**

	Code	Beschreibung/Bedeutung, inkl. Anwendung des Codes	Nicht zu verwechseln mit	Ankerbeispiel
		Wenn mehrere Textstellen für eine gleiche Kategorie gefunden werden (siehe hier das Beispiel), kann einen neuen Code angelegt werden.		**Finanzierung.** [I: Okay.] Es gibt ja in Deutschland nur die ein Gesetz für das (unv.) [redacted] -Register da wird auch von den Krankenkasse ein gewisser Betrag abgezweigt für das Register und es wird öffentlich finanziert allerdings auf einem Niveau sag ich mal sind welche Daten erhoben werden ist natürlich viel sind auf einem viel geringerem Niveau als jetzt unser Register aber wir müssen in die in dieses Qualitätssicherungsprojekt also alles was mit dem Register zu tun hat und auch mit den Projekten die da dranhängen geben wir eine halbe Million Euro pro Jahr aus [I: Okay.] und das finanzieren mit Spenden. (...) Und das ist natürlich ein Problem andere seltene Erkrankungen die noch viel seltener sind als unsere (lacht) nicht stemmen können ne [I: Ja.] und auch andere Organisationen das ist glaub ich mit der **Hauptgrund warum eben es für nicht nicht viele Erkrankung solche Register gibt.**“ [redacted]
Ethische Bewertungsdimensionen				
18		***Bemerkung**: Die Ethischen Bewertungsdimensionen werden separat kodiert. In der Analyse können diese Kodierungen mit den oberen Code-Kodierungen kombiniert werden, um spezifischere Aussagen zu treffen (z.B.: Nur zu digitalen Registern oder spezifischen digitalen Technologien).*<br>*Ggf. sollte entschieden werden, diese Codes in einem separaten Kodierungsschritt zu kodieren.*		
18.1	ETH_Soli	**Solidarität**: Die interviewte Person äußert sich dazu, dass eine reziproke Haltung mit einer Bezugsgruppe wichtig, oder im Gegenteil nicht wichtig, für die Nutzung digitaler Angebote, speziell das digitale Patient*innenregister, ist. Ein wichtiger Aspekt von Solidarität ist eine direkte oder indirekte	ETH_Altr [Einen Ausdruck der Solidarität ist nicht mit Altruismus zu	„[...] aber **vielleicht kann ich damit auch anderen helfen die meine Erkrankung haben** und sagen dass man dann mit diesen Daten forschen kann um eben der Allgemeinheit also dieser dieser **Allgemeinheit an (K[1])-Patienten** irgendwann in näherer oder fernerer Zukunft je nachdem helfen zu können ja.“ [redacted]

	Code	Beschreibung/Bedeutung, inkl. Anwendung des Codes	Nicht zu verwechseln mit	Ankerbeispiel
		Unterstützung von der Bezugsgruppe bzw. Personen in dieser Bezugsgruppe und ihrer Erfahrungen, Ziele und Aktivitäten.<br><br>Reziprozität nochmal nachschauen	verwechseln: Bei Solidarität muss eine Bezugsgruppe (im Hintergrund) stehen sowie eine Erwartung der Reziprozität vorliegen.]	
18.2	ETH_Altr	**Altruismus**: Die interviewte Person äußert sich dazu, dass die Nutzung digitaler Angebote, speziell das digitale Patient*innenregister, ein Ausdruck von Unterstützung oder Hilfeleistung gegenüber anderen ist, ohne dass hier Vorteile für sich Selbst oder eine Gegenleistung der anderen (z.B. anderen in einer Bezugsgruppe) erwartet werden.<br><br>***Bemerkung**: Mit den Definitionen der Solidarität und des Altruismus wird schon eine theoretische Entscheidung gemacht. Jedoch scheint mir dies erstmal sinnvoll, weil wenn wir letztendlich Solidarität als Subcode von Altruismus verstehen, können wir in der Analyse beide Codes zusammennehmen.*	ETH_Soli [Ein Ausdruck des Altruismus ist nicht mit Solidarität zu verwechseln: Bei Altruismus fehlt die Erwartung der Reziprozität, damit muss also nicht unbedingt eine Bezugsgruppe (im Hintergrund) stehen.]	„[...] **Und ja dann wenn es anonym ist dann kann es eigentlich nur helfen.** (...) **Wenn wenn nicht einem selbst dann vielleicht anderen oder für die Zukunft** oder (...) weiß ich nicht wenn man es, ja also eigentlich kann es nur helfen denk ich.“ [redacted]

	Code	Beschreibung/Bedeutung, inkl. Anwendung des Codes	Nicht zu verwechseln mit	Ankerbeispiel
18.3	ETH_EigNutz	**Eigennutzen**: Die interviewte Person äußert sich dazu, dass die Nutzung digitaler Angebote, speziell das digitale Patient*innenregister, aus Hoffnung für das eigenen Gewinn oder voranbringen eigenes Interesse – ohne Bezug zu den Interessen oder der Unterstützung anderer Personen – ist.		„ […] Hm ich hab mir gedacht dass wenn meine Daten dort angegeben werden (...) also dass ich diese angebe in der Hoffnung dass man damit **dass ich damit entweder mir selbst irgendwie helfe** indem diese Daten **die auf mich zutreffen irgendwann auf ein Medikament zutreffen** wo dann gesagt wird okay Patient X Y mit diesen Daten und andere Patienten mit diesen Daten auf die trifft das zu die können das nehmen“ [redacted]
18.4	ETH_Ger	**Gerechtigkeit**: Die interviewte Person äußert sich dazu, ob eine (un)gleiche Behandlung im Rahmen der Digitalisierung bzw. Zugang zu digitalen Produkten besteht (Gleichbehandlung), ob der Zugang und die Nutzung digitaler Produkte (un)gerecht verteilt sind oder ob die Behandlung von Personen fair ist.<br><br>Ein wichtiger Aspekt der Gerechtigkeit ist die Barrierefreiheit bzw. Zugänglichkeit und Nutzbarkeit von digitalen Angeboten für unterschiedliche Personengruppen.		*Zur Teilhabe oder Zugang:*<br>„[...] ein Aspekt der in Diskussionen in unserer Selbsthilfegruppe immer wieder auftaucht ist ja es gibt viele Ältere (...) die die neuen Hilfsmittel nicht nutzen. Und wir müssen auch für die was machen. Ja? (...) Die Konsequenz darf dann aber nicht sein dass man sagt na dann machen wir nichts im Bereich Digitales weil die Alten nicht mitkommen [I: Okay ja.] (...) weil DAS kann es aus meiner Sicht GAR NICHT sein. Also wenn dann muss man dann doppelgleisig fahren weil irgendwann sind die ist die ältere Generation halt eben ausgestorben oder wird immer weniger und wenn ich DANN erst mit digitalen Hilfsmitteln anfange dann ist es eigentlich zu spät. [I: Ja.] **Also man sollte direkt mit die digitalen Hilfsmittel einrichten soweit möglich (...) und als Backup für die die sich da eben nicht mehr reinarbeiten wollen auch noch eine Alternative anbieten.**“ [redacted]<br><br>*Zur Barrierefreiheit:*<br>„[...] ja ich hab mich eher über die über die **wirklich schlecht gemachte (...) Webseite** geärgert [I: Okay.] **weil es war halt eine Seite die von Sehbehinderten genutzt wird und dann ist die Struktur also extremst kleinteilig und (...) ja ohne Vergrößerung kaum** (lacht) [I: Erkennen ja.] **kaum handhabbar** also das das sind

	Code	Beschreibung/Bedeutung, inkl. Anwendung des Codes	Nicht zu verwechseln mit	Ankerbeispiel
				dann so Kleinigkeiten wo ich denk ey das hätte man doch vielleicht bisschen freundlicher für die Betroffenen auch gestalten können.“ [redacted]
18.5	ETH_Kontr	**Datensouveränität** (Kontrolle über die eigenen Daten): Die interviewte Person äußert sich dazu, ob Personen das Recht haben, über ihre eigenen gesundheitsbezogenen Daten zu entscheiden. Zum Beispiel sollte sie immer selbst entscheiden dürfen, welche Forschungsprojekte die eigenen Daten benutzen.		„**Das ist wichtig also wenn ich das Gefühl hätte da das ist irgendwie intransparent oder es ist nicht klar wer darüber bestimmt** oder oder die Mitarbeiter in der Geschäftsstelle könnten selber in Daten reingucken und überlegen (lacht) ob sie das jetzt auswerten oder so dann hätt ich natürlich meine Zweifel oder Sorgen und würde mich vielleicht auch anders entscheiden das ist jetzt ein bisschen fiktiv weil es eben nicht der Fall ist […]“ [redacted]
18.6	ETH_DS	**Datensicherheit**: Die interviewte Person äußert sich dazu, wie Informationen vor unberechtigter Verwendung und Verarbeitung sowie vor Verlust geschützt werden sollten (z.B. Zugriff, Speicherung, Manipulation und Weitergabe).		„[…] also dass man über eine App oder ein iPad oder so dann in diese persönlichen Auswertungen auch reingucken kann das ist nur natürlich dann noch mal eine Stufe schwieriger mit dem Datenschutz und mit der Datensicherheit ne da muss man ja also zum Beispiel eine ein **Identverfahren einführen** also da kann nicht jeder mit mit einem Account kommen und sagen ich will mich anmelden und registrieren und mit der E-Mail verknüpfen sondern da muss man wirklich sichergehen dass das DER ist [I: Ja.] der da seine Daten auch geliefert hat und entsprechenden kompliziert ist das natürlich.“ [redacted]<br><br>„**Da das wär natürlich bitter wenn dann irgendwo Arbeitgeber oder Institutionen die Möglichkeit hätten wenn sie jetzt in Kontakt mit mir treten wollen** für für was auch immer welche Tätigkeiten wo sie vielleicht Wert auf hundert Prozent Gesundheit legen und dann **womöglich doch einen Blick in die in die Patientenakte nehmen können** und sehen ups da schlummern ja riesige Risiken nee das machen wird nicht.“ [redacted]
18.7	ETH_Ver	**Vertrauen**: Die interviewte Person äußert sich dazu, ob sie sich auf die digitalen Angebote oder die		„Also ich brauch die persönliche Beratung meines Arztes die sagt das ist mir jetzt okay [I: Okay.] das läuft also grundsätzlich also eine

	Code	Beschreibung/Bedeutung, inkl. Anwendung des Codes	Nicht zu verwechseln mit	Ankerbeispiel
		Akteure, die die Digitalisierung im medizinischen Bereich vorantreiben, verlassen kann. Dies wird begleitet von der Annahme, dass die digitalen Angebote bzw. anderen Akteure nicht den eigenen Erwartungen, Interessen oder Wünschen entgegenstehen.<br>Dies kann auch inkludieren, dass die interviewte Person Personen und/ oder Institutionen benennt, denen sie (nicht) Vertrauen kann/ in die sie kein Vertrauen hat oder die sie als (un)zuverlässig ansieht.		grundsätzliche Erklärung so und so läuft das der und der hat dran mitgearbeitet das und das steckt dahinter und dann ist es okay oder **ich hab ein ein Vertrauen in die Institution also dass das Krankenhaus schon gute Instrumente geprüfte Instrumente evidenzbasierte Instrumente einsetzt** und nicht irgendeinen Unsinn [I: Ja.] also ja das ist grundsätzliches Vertrauen." [redacted]<br><br>„Hm (...) also ich glaube das liegt eben daran dass man dann sein Vertrauen mehr oder weniger in eine Maschine setzt (...) wo man halt auch weiß dass es beispielsweise Systemfehler geben kann oder so und ich glaube da kann man **wenn man das Vertrauen in einen Menschen setzt ist dann immer noch für einen selbst mehr Sicherheit da als wenn man es nur in eine in eine Maschine oder eine künstliche Intelli_ Intelligenz [I: Ja.] also eine Software setzt** weil das ist was was man dann wahrscheinlich nicht so greifen kann und sich auch nicht so vorstellen kann [...]" [redacted]
18.8	ETH_Trans	**Transparenz**: Die interviewte Person äußert sich dazu, dass es (un)wichtig ist, die Funktionsweise digitaler Produkte oder den Grund von Handlungen anderer Akteure verstehen zu können bzw. dass über die Ziele, die andere Akteure haben, offen und eindeutig kommuniziert wird.		„[...] ich kann ja wenn ich mehr wissen will auf die Homepage des (PO[3]) gehen das ist ja ganz **das ist ja alles sehr transparent dann kann ich [I: Okay ja.] sehen was was sind das für das Daten was macht man mit den Daten mit den Daten es gibt einen jährlichen Bericht des Registers einen wissenschaftlichen an den ich ranko_ ohne Mühe rangebe rankomme es gibt einen Patientenbericht also in patientenfreundlicher Sprache mit patientenfreundlichen Informationen den ich lesen kann** (...) das Register, wenn es wichtige Frage in Bezug auf den Register auf das Register gibt wird das in der (Abkürzung für K[1]) Punkt info also in der in der in in dem Newsletter diskutiert **ich kann mich an die Zuständigen in der Geschäftsstelle wenden wenn ich da Zweifel habe oder Fragen haben das ist ja total transparent** [...]" [redacted]

	Code	Beschreibung/Bedeutung, inkl. Anwendung des Codes	Nicht zu verwechseln mit	Ankerbeispiel
18.9	ETH_Emp	<u>**Empowerment**</u>: Die interviewte Person äußert sich dazu, dass es (un)wichtig ist, dass die Autonomie bzw. Selbstbestimmung von Personen oder Gruppen von Personen (wie Gemeinschaften) erhöht wird, sodass sie befähigt werden ihre Interessen und Bedürfnisse selbstbestimmt und eigenverantwortlich zu vertreten sowie sich an Entscheidungsprozessen beteiligen zu können.		„[...] um sich dann mit Menschen seinesgleichen sag ich jetzt mal oder mit Menschen die auch andere Sachen erlebt haben vielleicht austauschen zu können so was wär vielleicht auch noch eine Möglichkeit zur (...) Sozialisierung oder vielleicht auch wenn man dann in einer Art Loch steckt nach einer sch_ nach einer schlimmen Krise und dann da eben nicht mehr rauskommt dass man dann dazu auch **über das Patientenregister mit Menschen die auch einen ähnlichen Verlauf der Krankheit hatten dass man dann mit diesen sprechen kann sich Mut machen lassen kann mit denen über den Pat_ über das weitere Vorgehen zum Beispiel reden kann so was wär vielleicht auch noch eine tolle Möglichkeit zu der Erweiterung also die die spezifisierte Selbsthilfe** [...]“ [redacted]
18. 10	ETH_Sons	<u>**Sonstiges**</u>: Wenn die interviewte Person ethische Bewertungsdimensionen anspricht, die nicht in den oberstehenden Ausführungen inkludiert sind, dann sollten diese mit dem Code „sonstiges“ kodiert werden. Beispiele könnten sein: gerechte Kommunikation, Autonomie, epistemische Gerechtigkeit, Diversität, etc.<br><br>Wenn mehrere Textstellen für eine gleiche Kategorie gefunden werden (siehe hier das Beispiel), kann einen neuen Code angelegt werden.		*Zum Beispiel „Verantwortung/Haftung“:*<br>„Also **wer haftet beim Einsatz von digitalen (...) Anwendungen wenn es schiefgeht**? Wenn eine Diagnose zum Beispiel aufgrund der Tatsache dass nur Telemedizin eingesetzt würde NICHT rechtzeitig entdeckt wird. So als nur eine Frage. [I: Ja.] (...) Wer haftet dafür wenn das eingesandte Foto nicht gut genug ist um den kleinen winzig kleinen schwarzen Fleck zu entdecken?[,,,]“ [redacted]
