# Supplementary material for: Social factors associated with the development of e-health literacy: A mixed-methods study among patient organization members and representatives
Source: PLOS Digit Health. 2026 Aug 13;5(8):e0001569. doi: 10.1371/journal.pdig.0001569 (PMC13472428; doi:10.1371/journal.pdig.0001569)
Supplement: Supplementary file 4 [file pdig.0001569.s004.pdf]

I. Interview material

1. Coded passages on e-health literacy

Ich kann mir vorstellen dass wenn die gut ausgebaut ist dass man DA wirklich einen Nutzen hat weil man die Ergebnisse überall mit sich rumträgt aber da muss ich auch ganz ehrlich sagen ich bin mir gar nicht sicher ob ich IMMER will dass ALLE meine Daten dann dadurch erreichbar also einsehbar sind. Ich weiß dass ich bestimmte nur bestimmten Leuten Zugang erteilen muss dass ich auch nicht alles aufnehmen muss aber, das ist mir noch zu unklar ich hab mich einfach noch nicht genügend damit auseinandergesetzt um da jetzt wirklich Energie reinzustecken mir diese EPA zu besorgen.

Patient 2

ich finde die Expertise mit den elektronischen Gadgets dann auch umzugehen (lacht) das ist allerdings ein ganz ganz hohes Thema also ganz ganz schwieriges Thema. Ich selber bin da überhaupt nicht gut ich hab also, also von selbst auf mich geworfen einen ganz schlechten IT-Zugang ich hab aber einen Mann der mir dabei hilft (lacht) und damit kann ich dann auch anwenden aber ich bin überhaupt niemand der sich Anwendungen selbst erschließen kann. Also wenn man mir zeigt wie es geht kann ich anwenden logisch [I: Ja.] aber selber anwenden also, was weiß ich Apps aufladen oder mit Apps Apps dann auch umsetzen so also da hab ich echt Schwierigkeiten. Für mich ist das nicht sind die meisten Sachen nicht selbsterklärend

Patient 2

da brauch ich Hilfe und das geht sehr sehr vielen so also da besteht auch würd ich sagen seitens der Patienten die WIR zumindest vertreten also (PO[2]) vertritt ganz viel Angst und Unsicherheit und das ist auch richtig. Und dann ist kommt natürlich das Thema Barrierefreiheit. [I: Ja.] Also dass viele Menschen gehindert sind zum Beispiel Sehbehinderte mit diesen elektronischen Möglichkeiten angemessen umzugehen und das muss auf jeden Fall bei medizinischen Anwendungen berücksichtigt werden und zwar bei JEDER Anwendung.

Patient 2

es gehört einfach grundsätzliches Verständnis für IT-Prozesse würd ich sagen. Also die die Fähigkeit zu verstehen wie ich zum Beispiel etwas auflade wie ich mich identifiziere und authentifiziere im Netz dann das Gadget oder das, die die Anwendungen zum Laufen zu bringen dann die Anwendung zu verstehen was was sie also tun muss, eventuell die die Grafiken die mir gezeigt werden zu verstehen (...) aber, dann werden mir ja ständig Botschaften geschickt mach dies mach das, das muss ich einordnen können, also da ich denk so so Aktualisierungsprozesse und so weiter das muss ich einordnen können.

Patient 2

Ganz schwierig find ich zu entscheiden ist das Spam ist das Malware oder ist das jetzt ernst gemeint also (lacht) [I: Ja ja.] da gibt es manchmal wirklich Nachrichten wo wo ich wirklich in ins ins Schlingern komme das nicht entscheiden kann die sind so gut gemacht dass ich sagen kann ich weiß nicht ob das jetzt eine Phishing-Mail ist oder ob das eine eine echte Mail ist ich kann es nicht entscheiden, [I: Ja.] also all das spielt das spielt eine Rolle dabei und (...) also zumindest bei den Leuten die ich kenne sind natürlich alles ältere, also sind sehr viele ältere dabei, und die viel weniger Kontakt mit digitalen mit mit digitalen Prozessen habe als ich jetzt die sind da einfach sehr sehr verunsichert. [I: Ja.] Und wie gesagt ich würde mir das auch nicht zutrauen ohne meinen Mann.

Patient 2

ich nicht weiß ob man, wie viel Ex_ also, wenn man nicht mit diesen Möglichkeiten aufgewachsen ist wie viel Expertise man dann wirklich entwickeln kann also wenn ich das mit meinem Mann jetzt vergleiche der versteht das vom Grunde auf der weiß was dahinter passiert und ich weiß es halt nicht. Und damit kann ich immer nur kopieren nachmachen anwenden aber ich versteh nicht was dahinter ist und das erzeugt bei MIR tatsächlich auch ein großes Gefühl von Unsicherheit.

Patient 2

ich weiß es einfach nicht ob ich wenn ich eine bestimmte Anwendungen jetzt betätigt ob dann ein Risiko hin_ dahinter ist und sehr oft hoffe ich dass es also geh ich davon dass das keins ist aber ich kann nicht mit letzter Sicherheit entscheiden. Also diese diese, ich würde mir sagen dass ich in nichtige Teilen Gesundheitsentscheidungen also, wenn es also um was weiß ich meine Erkrankungen geht oder so was oder wenn es um um Informationen geht schon informierte Entscheidungen treffen kann also das erkenn ich jetzt an das erkenn ich jetzt nicht an da liegt vielleicht ein Risiko in der Quelle da nicht, das trau ich mir schon zu. Aber digital fällt es mir schwer informierte Entscheidungen zu treffen.

Patient 2

wo es um Anwendung von digita_ also um um Anwendung von digitalen Mitteln geht oder oder ja [I: Ja ja genau ja.] Mitteln sag ich jetzt mal, da sch_ braucht man eine Gesundheitskompetenz grundsätzlich also erst mal zu entscheiden ist das was für mich und das Zweite dann eine digitale Kompetenz.

Patient 2

Also von den Krankenkassen wüsst ich jetzt nicht die geben stellen ihre ihre elektronischen Patientenakten zur Verfügung und sagen jetzt kann_ du kannst dir das runterladen und so weiter aber eine Schulung was EPA meint das seh ich nicht bei den Krankenkassen [I: Ja.] ich kenn aber auch nicht alle Kra_ alle hundertsechzig Krankenkassen

Patient 2

Ich sehe es kann sein dass Volkshochschulen und so weiter so was anbieten die haben ja zum Beispiel früher auch Handyschulungen angeboten grad auch für Ältere was ich sehr gut finde, kann sein dass die auch elektronische Gesundheitsanwendungen zum Thema haben kann ich jetzt nicht sagen weiß ich nicht. [I: Okay ja.] Der Staat vernachlässigt es sträflich. Da wüsste ich jetzt gar nicht dass da irgendwas läuft und die Selbsthilfe macht es aus Eigenverantwortung und [I: Ja.] das wird dann eben gefördert also die entwickeln eigene Pro_ Projekte und das wird dann gefördert aber das ist natürlich auf völlig freiwilliger Basis.

Patient 2

eigentlich wäre es vielleicht eine Aufgabe von Krankenkasse und der Staat?

Patient 2: Könnt ich mir vorstellen ja ja ja. Also den Bürgern leicht zugängliche Programme anzubieten wie sie ihre digitale Kompetenz erhöhen. Die Frage ist natürlich wollen die das. [I: Ja.] (…) Also wird das dann auch angenommen.

Patient 2

Das wäre dann die Verantwortung des Einzelnen aber mein Gott man ist mit so viel [I: (lacht)] (lacht) Problemen beschäftigt ich kann mir gut vorstellen ich find es auch legitim zu sagen (lacht) nee da hab ich jetzt keinen Bock dazu also (lacht) ich hab jetzt genug zu tun.

Patient 2

Also ich würde jetzt darunter verstehen, wie Sie schon sagen, dass man ja irgendwie auch verstehen muss, dass wie die, also jetzt zum Beispiel ältere Leute, jetzt die Omi von von 86 Jahren die weiß vielleicht nicht unbedingt wie man sich für so eine Tele_ telemedizinische Kommunikation da jetzt vorbereitet, wie man denn an den Computer das anklicken muss oder da kann es tatsächlich irgendwie Probleme geben.

Patient 4

Dass man also schon Kommunikation auf jeden Fall, weil das ist ja das A und O, wenn man sich nur an der Kamera gegenübersitzt, kann man, kann das das Personal natürlich auch schlecht irgendwie beobachten, weil es ist ja ganz oft in der Medizin oder in der Pflege, dass man Patientenbeobachtungen macht und da schon so ein bisschen sehen kann, was vielleicht los ist. Da muss man sich natürlich auch gut artikulieren können, sprechen können und auch trotzdem dann ehrlich sein.

Patient 4

Ja, das wird mir jetzt gerade mal so einfallen. Natürlich auch irgendwie der überhaupt Zugang zu, dass das stattfinden kann. Also je nachdem wo man auch wohnt, vielleicht, nun ja.

I: Mit Zugang meinen sie zum Beispiel so Internetzugang, aber auch der Hardwarezugang und so weiter.

P4: Richtig, genau. Dann spielt ja auch der finanzielle Aspekt auch wieder eine Rolle. So was überhaupt anschaffen zu können. Ja, das wird mir jetzt so einfallen, spontan.

Patient 4

Wie wichtig erachten Sie das so? Wenn es geht um um und dass jemand das auch lesen kann und verstehen kann im Rahmen von digitaler Produkte?

P4: Ja, natürlich als sehr wichtig, weil man Gott, man kennt es aus der Medizin wie auf die Patienten sich irgendwie ärgern, dass sie irgendwelche Fachbegriffe natürlich nicht verstehen und können Sie mir das mal erklären und dann kriegen sie es nicht richtig erklärt. Das sorgt ja immer doch irgendwie für Aufreger.

Patient 4

und das würd ich jetzt auf die Digitalisierung übertragen also auch da bin ich in alle Richtungen immer neugierig und versuche eben immer so ein bisschen zu checken ist das für mich nützlich kann das bei (K[1]) was nützen und wenn ja dann muss man sich damit beschäftigen und (...) insofern

Patient 8

das Mindestmaß ist dass der Patient der sich auf eine solche digitale Anwendung einlässt a

Patient 8

Also dann er muss zumindest verstehen wie es funktioniert

Patient 8

Also dann er muss zumindest verstehen wie es funktioniert also am Beispiel des Zuckersensors der muss ja den Sensor an dem iPhone oder an dem Telefon anmelden können der muss erkennen können wenn irgendwie ein Fehler entsteht und und die Werte die da angezeigt werden vielleicht nicht stimmen er muss also Bescheid wissen was was sind normal wie sind die Werte einzuschätzen was sind die Konsequenzen wenn ich einen bestimmten Wert angezeigt bekomme

Patient 8

also digitale Kompetenz ist dann eben auch die die Auswertungen an den Arzt schicken zu können sich an- und abmelden zu können Login was weiß ich diese ganzen technischen Dinge muss er soweit beherrschen dass er mit gutem Gefühl diese Anwendungen benutzen kann

Patient 8

Da gibt es ja das schöne Wort von Albert Einstein schämen sollten sich die Menschen die die Errungenschaften der modernen Technik benutzen und dabei von der Technik so viel verstehen wie die Kühe von dem Gras das sie, von der, nee, die Kühe von der Botanik der Gräser die sie mit Behagen frisst

Patient 8

ja ja genau also man hat sonst unter Umständen Nachteile wenn wenn man diese technischen Dinge nicht nicht beherrsch

Patient 8

also ich glaube diese Gesundheitskompetenz wäre dann im im telemedizinischen oder im digitalen Bereich eben dass man dann halt die Fähigkeit hat sich selbst und seinen seinen medizinischen Zustand so einzuschätzen dass man seinem Gegenüber der jetzt nicht die Möglichkeit hat einen wirklich physisch zu beurteilen sagen kann was einem fehlt vielleicht auch wie sich das anfühlt vielleicht auch eine eine Beschreibung zu geben wie das au_ wie wie die die wie die Beschwerden aussehen sei es jetzt wenn sie wenn sie psy_ wenn sie physisch sind dass man eben die Stelle beschreibt die wehtut sag ich jetzt mal oder dass man eben na dass man eben diese Kompetenz besitzt eine detaillierte Beschreibung seines Zustands abzuliefern (4 Sek.) und halt gleichzeitig diese (...) hm moralische Kompetenz sag ich jetzt mal besitzt um halt zu sagen okay, das ist ein Angebot für mich weil ich jetzt vielleicht nicht die Zeit hab zu einem Arzt zu fahren weil der Arzt jetzt vielleicht auch nicht allzu viel Zeit hat um und Patienten die Wartezeit im Wartezimmer ersparen möchte aber das nutz ich jetzt nicht aus um Missbrauch zu betreiben oder um jetzt zu sagen okay (...) dass ich dieses System irgendwie ausnutz also ich denk mal diese Kompetenz muss auch gegeben sein dass man eben [I: Ja.] zwei diese beiden Kompetenzen hat sag ich mal

Patient 5

moralische Kompetenz sag ich jetzt mal besitzt um halt zu sagen okay, das ist ein Angebot für mich weil ich jetzt vielleicht nicht die Zeit hab zu einem Arzt zu fahren weil der Arzt jetzt vielleicht auch nicht allzu viel Zeit hat um und Patienten die Wartezeit im Wartezimmer ersparen möchte aber das nutz ich jetzt nicht aus um Missbrauch zu betreiben oder um jetzt zu

sagen okay (...) dass ich dieses System irgendwie ausnutz also ich denk mal diese Kompetenz muss auch gegeben sein

Patient 5

ein kleines medizinisches Grundverständnis hat aber eine (...) einigermaßen oder gut vorhandene soziale Kompetenz damit man sich eben auch traut ans Telefon zu gehen und diese, dieses Telefonat durchzuführen weil das hab ich jetzt in gerade in in in meinem Jahrgang also bei Leuten in meinem Alter seh ich das auch ganz oft dass die sagen nee ich drück mich vor so Sachen ich mach es wenn es notwendig ist aber [I: Ja.] (...) so gerne telefonieren oder so das ist ja heutzutage nicht mehr.

Patient 5

dass man eben sagt dass die Personen in der Lage sind sich selbstständig Informationen zum Beispiel im Internet über solche Angebote rauszusuchen und eben dann auch wahrnehmen zu können dass sie dass sie solche Programme bedienen können gut die sollten dann eben auch ein bisschen intuitiv sein dass die auch auf die Leute zugehen diese Programme (...) und jetzt nicht so hochgradig schwer dass da dass man da sich erst mal reinfuchsen muss sondern dass halt jeder der weiß dass es dieses Angebot gibt sollte dann auch find ich in der Lage sein dieses Angebot nutzen zu können (...) gerade bei Leuten die jetzt zum Beispiel kein Auto haben oder sich nicht leisten können mit der Bahn zum Arzt zu fahren (...) was ja auch teilweise auch eher alte Leute sind wobei da auch wieder schwierig ist diesen, eher älteren Menschen ein digitales Grundverständnis beizubringen weil das haben halt viele alte Leute nicht also ich kenn es auch mein, gerade meine meine Großeltern die kommen jetzt mit dem Handy oder so jetzt nicht allzu gut zurecht ich kenn auch Leute in dem Alter meiner Großeltern die haben nicht mal Internet (...) das ist da eine Gradwanderung das muss man gucken dass man diese Leute auch nicht hinten runterfallen lässt sondern dann auch vielleicht Telefonangebote macht die dann beispielsweise auch in der Zeitung oder im Amtsblatt stehen wo dann gesagt wird okay (...) wenn Sie jetzt ein medizinisches Problem haben können Sie eine Hotline anrufen oder auch dass über den Hausarzt solche Möglichkeiten angeboten werden können die eben auch telefonisch sein können und die Webseiten oder diese Dienstprogramme die eben mit Internet und digital funktionieren die sollten dann eben auch schon so intuitiv sein dass man das mit einem auch einem sehr niedrigen digitalen Grundverständnis hinkriegen kann natürlich ist so ein bisschen digitales Grundverständnis notwendig um diese Angebote auch suchen zu können aber die sollte jetzt auch nicht so (...) krass schwierig sein damit das halt auch von jedem in der Gesellschaft wahrgenommen werden kann.

Patient 5

ich denk mal dass, da sollte schon noch ein bisschen mehr dazu gehören so dass man eben seine Sachen auch also seine sein Leiden auch eben (...) beispielsweise auf einer schriftlichen Form oder (...) mit einer Anlage von Bildern verknüpfen kann (...) hm und darstellen kann

Patient 5

aber das könnte man vielleicht auch so lösen dass dass eine App auf dem Handy vielleicht auch dass dass man die entwickelt aufs Handy laden kann oder dass die sogar schon vorinstalliert ist bei manchen Handyanbietern und die App dann einem genau sagt jetzt können Sie einsprechen oder auch schreiben was Sie was Ihnen fehlt jetzt können Sie ein Bild davon machen (...) dass man das vielleicht auch so auf einer in einer App-Form so oder oder in einer in einer Programmform so darstellt oder eben dass man halt auch sagt es gibt so Telefonanbote -angebote gibt (...) mindestens meinem einem Telefon kommt ja eigentlich

zurecht und dann könnte man das auch so darstellen dass man dann über über diese über eine Hotline die dass dann auch erklärt das kann ja dann auch erst mal eine (...) eine Stimme sein die eingesprochen ist und dass man eher dann wenn man diese diese Erklärung hat über diese Hotline dass man dann zu einem Menschen weitergeleitet wird der dann mit einem redet also das fänd ich auch ganz wichtig dass so eine bei der Telemedizin immer ein Mensch dahintersteckt und nicht immer nicht nur eine Stimme die ansagt so so dass dann halt auch mit einem Menschen interagieren und sprechen kann.

Patient 5

Ja also wenn ich mir jetzt zum Beispiel vorstelle ich hätte irgendwie eine App und (...) da ist sozusagen hab ich Zugriff auf meine ganzen Patientendaten und indem ich jetzt zu einem anderen Arzt gehe und dem mich mit dem da quasi verbinde und sage ja du darfst Zugang zu meinen Daten haben würd ich schon wollen dass derjenige auch zumindest auch zu einem bestimmten Grad irgendwie da was mit mit mit anfangen kann ne.

Patient 9

Na ja ich glaube es ist wichtig dass es immer noch klar ist dass es ja sich um wirklich (...) wichtige und auch für den denjenigen also für den Patienten sehr bedeutsame Informationen sind ne. Für den einen ist es vielleicht nur eine Statistik ne [I: Ja.] was weiß ich fünfzig Prozent der Patienten mit der Krankheit können nur halbtags arbeiten aber für einen selbst ist es natürlich sehr bedeutend weil es für einen sehr viel Nachwirkungen hat im im [I: Ja.] im Leben.

Patient 9

Hm also (...) schon, irgendwo ja also hm hm, ich kann jetzt keinen Grad der Kompetenz nennen oder ähnliches aber man, muss, sicherlich, (...) zum einen da das notwendige technische Verständnis auch haben um mit diesen Medien umzugehen denn, jetzt werden auch die (K[1])-Erkrankten ja auch immer älter sprich oder auch die Angehörigen (...) da, ist es ja jetzt mit dem technischen Fortschritt mit, nur dem normalen E-Mail-Verkehr in der Regel nicht getan, (...) man muss einen Computer haben man muss sich eventuell Zoom-Meeting oder Teams et cetera mit diesen Programmen auch ein Stück weit auskennen und da kann ich mir durchaus auch vorstellen dass da auch irgendwo Berührungsängste bestehen wenn man einfach (...) mit diesen Medien noch nicht umgehen kann (...) dass das ein Hemmnis sein könnte.

Patient 10

Hm (...) ja also einfach, (...) also mein Verständnis wäre jetzt dass man mit dem Computer umgehen kann und (...) dort auch mit gängigen technischen Programmen ich weiß nicht wie ich es anders beschreiben soll halt, Teams Zoom und ja, (...) ja das, mehr fällt mir jetzt ehrlicherweise dazu nicht ein.

Patient 10

Ach so ja und (...) das, spielt, da sicherlich auch mit rein aber, ehrlicherweise, wenn ich jetzt von meinem, normalen Alltag ausgehe wo ich auch täglich sozusagen mich digital bewege (...) ist genau bei diesen Punkten da denkt ich ehrlicherweise gar nicht groß weiter drüber nach [I: Okay.] (...) weil ich dann, (...) für mich jetzt, als normaler Verbraucher immer denke okay (...) muss ich jetzt ohnehin akzeptieren sonst läuft die Seite vielleicht auch nicht wie auch immer so ist ich ich befass mich da ehrlicherweise (...) nicht in der Tiefe mit diesen Dingen.

Patient 10

Ja in in erste Linie natürlich technisches Verständnis also man muss zum einen natürlich wissen was die Geräte können (...) man muss aber verstehen (...) welche Daten erhoben werden und welchen Zweck die erfüllen und halt auch mit den dementsprechenden Risiken also nicht ha_ dass man halt nicht nur über das Positive aufklärt sondern auch welche negativen Dinge man aus solchen Daten halt lesen könnte.

Patient 7

Aber es ist halt sch_ ist halt schon sehr umfangreich und ich denke (...) ja dass Menschen die halt nicht mit dem Internet groß geworden sind oder sich halt auch wenig mit (...) ja IT-Security auseinandersetzen oder (...) solchen Themen (...) da vielleicht Schwierigkeiten bekommen könnten. Also man sieht ja (...) dass sehr viele Leute persönliche Daten ins Internet stellen und sich dessen gar nicht bewusst sind was was letztendlich alles damit passiert. Und gerade bei Gesundheitsdaten find ich es halt sehr problematisch.

Patient 7

Ja die Ursachen für Misstrauen sind (...) zum einen natürlich mein technisches Verständnis da drüber wie wie die Technik funktioniert und dass halt nichts perfekt läuft

Patient 7

ich kann das sehr wohl verstehen wenn jemand eine Handybenutzung beispielsweise grundsätzlich ablehnt (unv.) Ausbeutung und so weiter also dieser Themenbereich (unv.) mir kommt kein Handy ins Haus und damit ist er eigentlich von der gesamten Technologie also der der des Smartphones mit den Apps ist er eigentlich von vornherein mal ausgeschlossen [I: Ja.] und kann sich auch demzufolge überhaupt keine Kompetenz im Umgang mit dem Smartphone und mit den mit den Apps erwerben

Patient 6

wie man da (...) sozusagen eine (...) eine Kompetenz (...) ausrichten könnte die ohne Benachteiligung für für alle gilt das ist natürlich sehr schwierig aber auch da denke ich ist ist es un_ also unglaublich wichtig dass man eben auch in der Ausbildung sei es Schule oder wo auch immer also auch wirklich Medienkompetenz sich aneignet und also nicht nur die normale Handynutzung SMS Telefonieren Netflix was weiß ich (...) Spotify WhatsApp und so weiter lernt sondern auch (...) diese Kompetenz erweitert zum Beispiel auch auf den medizinischen Sektor.

Patient 6

Also man muss natürlich offen sein für für für die Technik also ich kenn aus meinem Bekanntenkreis welche die sind da sehr ja eher zurückhaltend. Und obwohl ich denen das zutrauen würde aber die das Int_ das Interesse ist da nicht so groß [I: Okay.] und ich sag immer ja ihr müsst euch da mal mit beschäftigen weil das kommt irgendwann. Das ist irgendwann Standard und dann sitzt ihr da und wisst das nicht. Und ja ich hab da also.

Patient 1

Ja klar also was ich über über Apps und so die müssen auf jeden Fall barrierefrei sein also jemand der schlecht gucken kann der müsste das. Das müsste so auch sein dass so hergestellt werden dass die Leute da das auch bedienen können zum Beispiel Sprach_ Sprachausgabe Spracheingabe oder Menschen die vielleicht eine geistige Behinderung haben die das vielleicht so in dieser leichten Sprache bekommen kennen Sie das?

Patient 1

Hm ja was müssen die haben die müssen erst mal auch eine, ja ja die müssen ein ja das ist auch vom Kopf her vielleicht fit sein also ja es gibt ja auch viele ältere Menschen die das vielleicht wollen aber nicht mehr verstehen können da dass Alter halt denen (...) ja und und weil die muss man ja auch irgendwie berücksichtigen

Patient 1

das sind auch sehr komplexe Texte oft im Datenschutz oder die die Impressum das das.

Patient 1: Das liest kein Mensch durch also die wenigsten die lesen [I: Ja.] das durch also.

Patient 1

Das kann man das kann man machen man kann ja auch Gesetzestexte vielleicht ein bisschen bisschen leichter formulieren ne also ich hab vor kurzem dieses Elster ausgefüllt ja da hab ich auch ein paar Anläufe gebraucht bis ich das verstanden habe. (...) Weil das einfach dieses Be_ dieses Behördendeutsch das hab ich auch nicht gleich verstanden. [I: Ja.] Und nachher also nachher hab ich es hinge_ hab ich es auch geschafft aber das hat gedauert und das man ja man sollte diese diese ganze Texte ein bisschen ja bisschen ab_ also von der Verständnis also verst_ verständlicher machen so.

Patient 1

also wenn man so ein bisschen digital affin ist dann ist ja Google schon mal ein ein guter Doktor. (...) Ne also je nachdem was man hat man ma_ man googelt ja [I: Ja.] und guckt was was wird da so an Ergebnissen rausgeworfen und wertet dann für sich kann das auf mich jetzt zutreffen kann es nicht zutreffen je nachdem was es was man hat es gibt Patienten Selbsthilfeorganisationen wo es Infomaterial gibt Informationsveranstaltungen wo dann auch gerne mal Ärzte referieren da kann man sich Informationen mitnehmen und bewerten und vielleicht auch abgleichen mit dem was man selber erlebt hat.

Patient 11

Patienten EPA Kompetenz wenn ich selber entscheide

Patient 11

Hm (...) also man muss schon glaub ich relativ digital affin sein um das (...) selber schaffen und (...) ja kontrollieren zu können.

Patient 11

ich glaube das das ist nicht oder noch (...) nee es ist nicht GANZ so einfach. (...) Also ich, (...) hab halt von vielen Älteren gehört die da sagen nee das ganze Digitale da hab ich mich nicht beschäftigt will ich mich nicht mit beschäftigen (...) ich bin im Prinzip damit aufgewachsen hab dann schon früh gesagt Computer muss man sich mit beschäftigen jetzt das Digitale ist für mich einfach auch ein Hilfsmittel um den Kontakt zur zur Außenwelt zu halten also gerade für für Sehbehinderte Smartphone Tablet sind mit die die wichtigsten Hilfsmittel die man hat [I: Okay ja.] (...) weil die ja das Stichwort ist dann barrierefrei (...) nicht hundert Prozent aber doch so eingerichtet sind dass man auch bei einem reduzierten Sinn die trotzdem noch gut nutzen kann und an Informationen rankommt. [I: Okay ja. Also.] (...) Also ich hab hier auch (...) also ich hab einen einen Riesenbildschirm damit ich eine gewisse Vergrößerung habe ich hab einen Screenreader der mir der mir vorliest ich hab gelernt die (...) ja (...) blind zu schreiben (...) und das das hilft natürlich [I: Ja.] ne. Gut beruflich musst ich mich eh

Software Computer war sozusagen das das Hauptarbeitsgerät da ja da muss man eben schon fit [I: Ja.] sein um das (...) dann auch entsprechend nutzen zu können.

Patient 11

also ich weiß nicht wie ich jetzt wie jetzt Fitness noch weiter weiter spezifizieren soll ich weiß jetzt auch nicht ob ich wirklich fit bin (...) also ich bekomm es hin meinen meinen Computer zu bedienen meinen Computer einzurichten ich hab es auch hinbekommen den einzurichten in einer Phase wo ich wo ich wirklich noch schlechter gesehen hab als jetzt. [I: Okay.] (...) Mein Smartphone ist noch nicht eingerichtet dass ich es blind bedienen kann ich bin da durchaus noch auf Restsehvermögen angewiesen.

Patient 11

Also man (...) ja man wächst damit je nachdem wie alt man vielleicht war wie man wie man groß geworden ist was man alles gemacht hat [I: Ja.] und man muss halt auch gewillt sein sich da sich sich da reinzuarbeiten und zu üben zu lernen.

Patient 11

Also bei der Krankenkassen-App ist es halt so man muss halt nur wissen können oder man muss nur wissen halt wie man die Dokumente hochlädt (...) und da einreichen und so weiter da braucht man kein großes Fachwissen man muss halt nur so ein bisschen die Technik kennen

Patient 12

also ich, gucke da mal drauf da, ich mein klar man kennt so Blutdruck achtzig bis hund_ oder hun_ hundertzwanzig zu achtzig so heißt das ja glaub ich ne und Puls irgendwie was um die sechzig und so das hat man so grob im Hinterkopf weil man es immer mal gehört hat wenn die Ärzte sagen ja okay Blutdruck ist normal alles gut und so aber es ist jetzt nicht so dass ich mich jetzt so völlig versteife auf meine Uhr und sage okay ich muss jetzt ein bisschen langsamer gehen weil mein Puls so hoch ist (...) das ist für mich einfach eine (...) hm ja wie soll ich das sagen so ein Art Richtwert na Richtwert auch nicht aber eher so eine nette Information so nebenbei einfach mal zu gucken so also ich, ich, musste einmal schmunzeln als eine Kollegin zu mir sagte sie hätte ihren Mann gefragt wie er denn die Nacht geschlafen hätte und dann wo er dann sagte ja Moment ich muss mal gerade auf meine Uhr gucken [I: (lacht)] (lacht) (...) also da hab ich auch nur gedacht also wenn ich aufstehe ich merke ob ich gut oder schlecht geschlafen habe ob ich gerädert bin völlig groggy bin oder wie auch immer [I: Ja.] (...) wenn ich danach noch auf die Uhr gucke ist es meistens auch so dass es dann bestätigt wird wenn ich morgens total müde bin dann hab ich auch wenig geschlafen ist ganz klar (...) da muss ich jetzt nicht unbedingt meine Uhr befragen ist halt immer ganz nett so wie die Schlafphasen so sind was mich auch schon mal interessiert einfach wie ich mich im Bett rumwälze die ganze Nacht teilweise weil ich ganz anders aufwache wie ich einschlafe ungefähr (...) aber das ist einfach für mich eine nette Information aber ich halte mich da jetzt nicht so extrem dran wo ich denke okay jetzt hast du nur so einen Puls jetzt musst du ein bisschen höher und jetzt musst du ein bisschen runter und jetzt musst du ein bisschen ausruhen und so weiter da acht ich nicht drauf ich versuch auf meinen Körper zu achten und wenn der sagt es ist Feierabend dann ist Feierabend (...) wie gesagt die die die Uhr ist einfach eine nette Information und so ist es dann zum Beispiel die ist dann ja auch mit GPS und so und dann kann ich halt wenn ich zur Arbeit fahre mit Rad zum Beispiel oder so

Patient 12

dann nehm ich das auf und sehe halt wie lange ich jetzt ungefähr gebraucht habe und wie schnell ich gewesen bin das ist dann nette Info aber es ist nicht so dass ich da jetzt voll die Kompetenz haben muss so ungefähr also das nee

Patient 12

Ja also viel manche die achten die achten ganz viel auf ihre Uhr wenn die dann auch noch laufen und was weiß ich und keine Ahnung wie schnell und Puls und hast du nicht gesehen wenn man die ganzen Läufer Marathonläufer oder so was was auch immer anguckt aber für mich ist es einfach eine nette Spielerei weil ich sage ich so der Arzt sagt mir ob es mir gut geht oder mein Körper sagt mir ob es mir gut geht oder nicht

Patient 12

Hm na ja dass man aus selbstermittelten Daten oder Daten die einem unmittelbar mitgeteilt werden von irgendeinem Gerät dass man daraus selber Behandlungsentscheidungen ableiten kann. Und das ist gerade beim (K[3]) ist das ja dann auch eine ganz also das ist gehört ja zum alltäglichen Leben ich bekomme von dem CGM halt angezeigt die Verlaufskurve der letzten Stunden kann mir überlegen was möchte ich jetzt essen und darauf aufbauend bestimme ich dann wie viel (Mk[19]) ich mir injiziere beziehungsweise wäre ja auch möglich wenn ich eine Pumpe zum Beispiel tragen würde dann würde ich ja auch darauf aufbauend die Entscheidung treffen was ich dann jetzt mit der Pumpe veranstalte

Patient 13

Hm na ja als Patient oder Anwender muss man auf jeden Fall verstehen (...) wie wie die Messwerte entstehen und wie sie ermittelt werden und dann muss man in der Regel durch eine Schulung eben in die Lage versetzt werden auf die gemessenen Werte zu reagieren. (...) Und das hängt dann natürlich sehr vom ich sag mal Bildungsniveau und auch von der von der Bereitschaft desjenigen an sich ab sich mit seiner Krankheit auch zu beschäftigen. Also es gibt ja gerade im im jugendlichen Alter ich kenn das auch noch aus meiner (lacht) aus meiner Jugendzeit hat man alles Mögliche oder interessiert man sich für alles Mögliche aber eben nicht für die chronische Erkrankung mit der man rumrennt [I: Ja.] und da würden einem auch irgendwelche superintelligenten Computer oder digitalen Systemen weniger helfen wenn man dann aber selber zu der Erkenntnis gelangt ist dass es doch wichtig ist und dass man sich damit ja auch die Lebenszeit verbessern kann wenn man diese (...) Instrumente nutzt dann besteht eben auch eine eine hohe Bereitschaft sich darauf einzulassen und sich damit zu beschäftigen

Patient 13

Das stimmt das hab ich auch selber schon beobachtet also es war ja vor einigen oder die Blutzuckerselbstkontrolle ist ja noch gar nicht so alt (lacht) und es gab jetzt so vor kurzem so einen so einen Schwung dass auch hochbetagten Menschen denen wird dann halt so ein so ein Blutzuckermessgerät in die Hand gedrückt also dann sollen sie sich selber in die Finger stechen und dann die Werte aufschreiben und man hat dabei aber gar nicht beachtet dass solche Menschen ja häufig mit digitaler Technik sowieso nicht so viel zu tun haben und dann ist es eben dazu gekommen dass manche Rentner in ihren Schubladen drei oder vier Blutzuckergeräte liegen haben von denen sie gar keins benutzen. (...) Weil eben auch die ja die Schulung nicht richtig stattgefunden hat und denen auch nicht klar gemacht werden konnte was das eben für Vorteile haben kann (...) so ein Blutzuckermessgerät auch intensiv zu nutzen. (...) Ja und das ist mit den CGM-Sensoren jetzt genau das gleiche also es hilft nicht jemandem einfach nur so einen Sensor an den Oberarm zu kleben man muss den Leuten auch

sagen und zeigen wie sie damit umgehen und mit den gemessenen Werten eben richtig richtig verfahren.

Patient 13

Na digitale Kompetenz also erstens ich muss mich damit auskennen wie wie (lacht) das Gerät im Normalfall funktioniert [I: Okay ja. (lacht)] ich muss aber auch die Möglichkeit haben oder es muss mir gesagt worden sein auf Fehler Fehlermeldungen angemessen zu reagieren. Also wenn ich die Anzeige bekomme Batterie ist leer oder jetzt bitte aufladen dann gibt es eben wirklich Leute die nicht digital affin sind die das Gerät dann erschreckt in die Schublade tun und es nie wieder anfassen und (lacht) es [I: Ja.] Leu_ Leute die so nach 1970 80 geboren sind die wissen ah ja jetzt muss ich mein Ladegerät rausholen und das genau wie ein Handy wieder aufladen aber jemand der jetzt schon achtzig ist oder noch älter der hat das ja nie gemacht unter Umständen [I: Ja.] (...) und da, also bevor Ärzte oder Kliniken den Patienten solche Sachen in die Hand geben muss eben auch geguckt werden wie ist dieser Mensch ge_ generell so aufgestellt und bringt das auch etwas dem so ein Gerät zu geben kann er damit umgehen also das wäre ganz wichtig.

Patient 13

ja ich glaub das hm (...) ist jetzt die Frage wie wie kann jemandem das, beibringen (...) welche Quellen (...) sich jetzt gut anhören und welche jetzt (...) ja schlecht sind also (...) ja so was müsste man naja (...) Leuten die halt irgendeine Krankheit kriegen oder irgendwas so eine so eine digitale Schulung geben.

Patient 3

Ja gut die meisten Leute (räuspert sich) kennen sich ja schon ein bisschen mit Computer und Internet aus (...) das ist halt ja (...) hm [I: Ja.] (...) ich also hm in meinem Privatumfeld, wieder die zwei Extreme ich die halt beruflich damit zu tun hat (...) und da viel mehr ausprobiert und viel mehr guckt und auch weiß worauf sie ein bisschen achten muss und meine Schwiegermutter die hat damit überhaupt nichts zu tun [I: Ja ja.] (...) weil die das alles böse ist ja (...) ja und aber es ist ne alle einzufangen (...) das ist halt einfach denk ich nicht möglich.

Patient 3

Also die digitale [I: Ja.] Patientenakte bringt meiner Schwiegermutter wahrscheinlich gar nichts. (...) Mir aber wahrscheinlich sehr viel also. [I: Ja.] (...) Ne ich würd es mir auch anschauen wollen meine Schwiegermutter (lacht) (...) würde nicht mal die die Tastatur berühren also.

Patient 3

Ich denk auch mit Interesse. (...) Also ich sag jetzt nicht dass irgendjemand irgendwas nicht könnte. [I: Okay.] (...) Weil man sich halt, (...) das Interesse nicht so groß ist oder vielleicht auch die Zeit nicht dafür da ist sich damit auseinander zu setzen. Ist ja auch manche verstehen es schneller manche halt nicht so schnell (...) ne man hat halt dann oft auch keine Zeit und wenn man nicht Interesse hat da da noch mal die Zeit aufzuwenden sich damit auseinander zu setzen.

Patient 3

wenn man nicht Interesse hat da da noch mal die Zeit aufzuwenden sich damit auseinander zu setzen. [I: Ja.] Also es gibt auch genug Themen auf die hab ich auch hab ich auch keine In_ kein Interesse und [I: Ja.] (...) ne pff kenn ich mich nicht aus und.

Patient 3

2. Coded passages on health literacy

Manchmal will ich nicht dass eine Diagnose gefällt wird weil ich keine Lust habe das behandeln zu lassen dann lass ich es lieber laufen. Und das wird natürlich schwieriger wenn ich alle Daten gesammelt habe.

I: Ja. Ja das verstehe ich.

P2: Also wenn ich zum Arzt gehe und und ein Wert taucht auf dann der nicht der Norm entspricht dann wird der werden sofort Strategien ausgelöst also Behandlungsstrategien, was ja auch richtig ist, aber ich hab so viele Baustellen persönlich, okay das wird hoffentlich gestrichen kann, dass ich manchmal keine Lust mehr habe. [I: Ja okay.] Und dann ist es für mich leichter das laufen zu lassen und nicht gleich behandeln zu lassen.

Patient 2

Ich würde als Gesundheitskompetenz seitens des Patienten beschreiben [I: Ja.] dass ich die (...) gesundheitlichen, Fragen die ich habe angemessen beantworten oder lösen kann. Das heißt dass ich in der Lage bin zu wissen wo frage ich nach wo hole ich mir Informationen also gültige Informationen wo finde ich Informationen wie baue ich die ein wo will ich Informationen haben und wo will ich keine Informationen haben. (...) Also dass ich bewusste Entscheidungen über meine Gesundheit fällen kann (...) ja so würde ich das umreißen.

Patient 2

Das heißt dass ich in der Lage bin zu wissen wo frage ich nach wo hole ich mir Informationen also gültige Informationen wo finde ich Informationen wie baue ich die ein wo will ich Informationen haben und wo will ich keine Informationen haben.

Patient 2

Also die Informationen bek_ sich holen und bekommen die man benötigt wissen wo ich sie bekommen das ist ja davon eingeschlossen und dann informierte Entscheidungen treffen können.

Patient 2

Na ja ich hab ja erst erzählt dass ich zu den Patientinnen gehöre die nicht alles behandeln lassen wollen und dass ich da meine eigene Entscheidung (...) mir vorhalten (...) also m_ dass ich möchte dass diese Entscheidung bei mir bleibt. Und dazu gehört natürlich auch dass ich sage nee ich besorg mir jetzt keine Informationen da drüber und ich such jetzt nicht jedes Hautbild im im Internet um da zu schauen was ist es jetzt und ich kümmer mich um dieses Symptom jetzt eben nicht. Und das das mein ich damit. [I: Ja.] Also ich (...) ich muss ich nicht nicht jeder körperlichen Erscheinung hinterherjagen und prüfen ist die ist das noch im im normalen Bereich oder ist das schon eine neue Erkrankung. So meine ich das, also [I: Ja.] ich behalte mir vor bestimmte Diagnosen oder bestimmte Erscheinungen nicht weiterzuverfolgen ich entscheide drüber will ich da eine Behandlung eine Diagnose und Behandlung oder will ich da eben nicht und das ist (...) ja und, darüber dann möglichst da möglichst eine begründete Entscheidung zu zu fällen

Patient 2

inwiefern (...) muss ein Person wirklich ein Experte oder Expertin sein?

P2: Nee das würd ich nicht sagen nein. Das wäre (lacht) auch eine viel zu hohe, (lacht) das wäre auch eine viel viel zu hohe Anspruch. [I: Ja.] Also die die nein nein nein. Also ich finde jemand mit einem gesunden Menschenverstanden also der gesunde Menschenverstand der sollte ausreichen in Anführungszeichen gesunder Menschenverstand natürlich. [I: Ja okay.] Also das was was ein Durchschnittsbürger bei etwas (unv.) zum Thema dazu wissen kann das sollte nu wirklich ausreichen.

Patient 2

wo es um Anwendung von digita_ also um um Anwendung von digitalen Mitteln geht oder oder ja [I: Ja ja genau ja.] Mitteln sag ich jetzt mal, da sch_ braucht man eine Gesundheitskompetenz grundsätzlich also erst mal zu entscheiden ist das was für mich und das Zweite dann eine digitale Kompetenz.

Patient 2

Also es ist wie wie überall in der Medizin informierte Entscheidung und ich muss die Informationen haben um eine informierte Entscheidung zu treffen. [I: Ja.] Und die dürfen auch nicht (lacht) wie das so manchmal passiert zwischen Tür und Angel passieren sondern (lacht) die sollten da sollte man sich Zeit für nehmen.

Patient 2

Aber, was verstehe ich noch unter Gesundheitskompetenz? Ja. Würde ich jetzt jetzt nicht bezogen auf irgendwelche digitalen Produkte, sondern unter ja, verstehe ich natürlich jetzt auch, wenn man mit der eigenen Gesundheit so ein bisschen umgeht, also wie, wie verantwortungsbewusst man mit sich umgeht, wie man sich selber und seine Gesundheit halt fördert. Auch hinsichtlich Prävention, wie man damit umgeht und so ein bisschen Selbstpflege und und das verstehe ich natürlich auch so darunter.

Patient 4

Wie wichtig erachten Sie das so? Wenn es geht um um und dass jemand das auch lesen kann und verstehen kann im Rahmen von digitaler Produkte?

P4: Ja, natürlich als sehr wichtig, weil man Gott, man kennt es aus der Medizin wie auf die Patienten sich irgendwie ärgern, dass sie irgendwelche Fachbegriffe natürlich nicht verstehen und können Sie mir das mal erklären und dann kriegen sie es nicht richtig erklärt. Das sorgt ja immer doch irgendwie für Aufreger.

Patient 4

Ja ich also ich persönlich jetzt von mir kennt auch Gott, man kriegt da 1000 Seiten in die Hand gedrückt, das unterschreibt man einfach ohne es zu lesen und daheim fliegt es in die Tonne, ja. Also weil die Leute gar keinen Bock darauf haben, das alles zu lesen, geschweige denn aufzuheben. Warum sollte man das aufheben? Ja, das finde ich auf jeden Fall, dass das noch totaler Verbesserungs_ [Rauschen]_bedarf. Ja, das kann man noch. Also Verbesserungspotenzial vorhanden. Auf jeden Fall. Ja

Patient 4

Also ich finde, die Leute sollten dem Gegenüber schon irgendwie offener werden, weil nur so können ja die Prozesse halt auch irgendwie verbessert werden. Und ja, ich finde, da könnte vielleicht auch einfach noch ein bisschen mehr Aufklärung stattfinden, tatsächlich. Und Erläuterung und und ja, ich finde, da könnt einfach noch ein bisschen mehr kommen. Tatsächlich.

Patient 4

unter Gesundheitskompetenz so viel zu verstehen von der Erkrankung von der Therapie von den Medikamenten von den Maßnahmen die man generell gegen die Erkrankung ergreift über den Lebensstil und so weiter dass ich möglichst gut mit der Erkrankung zurechtkomme. Und das bedeutet eben lebenslanges Lernen

Patient 8

ich hab da vor zwanzig Jahren einen Patienten über den Verein kennen gelernt der Marathon gelaufen ist und dann bin eben hab ich sehr langsam ich konnt damals irgendwie dreihundert Meter laufen oder so und dann hab ich gehustet und konnt nicht mehr aber ich bin dann zum Laufen gekommen und hab inzwischen viermal Marathon gelaufen und [I: Wow.] also diese das hätte ich gar nicht früher gar nicht für möglich gehalten ne [I: Ja.] dass ich überhaupt mal soweit komme und diese diese Wege also neue Wege mit der Krankheit umzugehen die sich da auftun das funktioniert eben nur wenn man Kontakt zu anderen Patienten hat

Patient 8

wenn man neugierig ist wenn man die Kompetenz immer weiter verstärkt und aktualisiert das ist ja bei der Erkrankung auch so dass wenn man irgendwie ein paar Jahre aufhört zu lesen und sich neue Erkenntnisse anzueignen dann stimmt das alles auch gar nicht mehr weil es sich eben so schnell ändert

Patient 8

Ich mein Eltern die mit einem kleinen mobilen Lungenfunktionsgerät zweimal in der Woche die Lungenfunktion von ihrem Kind nis_ messen erkennen halt innerhalb von wenigen Tagen dass ein Problem da ist und bei anderen Familien wo das einfach ignoriert wird dann fällt es nach drei Monaten auf wenn der nächste Arztbesuch fällig ist und dann ist natürlich die Reaktion viel zu spät und also ist nur als Beispiel ne

Patient 8

Also das ist [I: um.] abhängig von der vom Schweregrad der Erkrankung also die (K[1]) ist per se tödlich und die Therapiemaßnahmen sind unglaublich zeitaufwendig ich hab also bis zu vier Stunden früher Atemtherapie machen müssen am Tag und je mehr ich davon verstehe und je mehr ich das optimiere umso wirksamer ist und umso mehr kann ich diesen Zeitaufwand begrenzen und nur so ist es dann möglich nebenher noch zu arbeiten und (lacht) andere Dinge zu machen also von daher [I: Ja.] würd ich sagen immer das Höchstmögliche Bestmögliche die bestmögliche Kompetenz erreichen (...) aber das trifft natürlich nicht zu wenn einer Asthma hat und kriegt ein Spray und kann damit ganz normal leben und hat auch keine bedrohliche weiter Verschlechterung zu erwarten dann dann kann man das nicht erwarten dass sich jemand so so komplett auf die Erkrankung konzentriert

Patient 8

der muss erkennen können wenn irgendwie ein Fehler entsteht und und die Werte die da angezeigt werden vielleicht nicht stimmen er muss also Bescheid wissen was was sind normal wie sind die Werte einzuschätzen was sind die Konsequenzen wenn ich einen bestimmten Wert angezeigt bekomme

Patient 8

ich glaube diese Gesundheitskompetenz wäre dann im im telemedizinischen oder im digitalen Bereich eben dass man dann halt die Fähigkeit hat sich selbst und seinen seinen

medizinischen Zustand so einzuschätzen dass man seinem Gegenüber der jetzt nicht die Möglichkeit hat einen wirklich physisch zu beurteilen sagen kann was einem fehlt vielleicht auch wie sich das anfühlt vielleicht auch eine eine Beschreibung zu geben wie das au_ wie wie die die wie die Beschwerden aussehen sei es jetzt wenn sie wenn sie psy_ wenn sie physisch sind dass man eben die Stelle beschreibt die wehtut sag ich jetzt mal oder dass man eben na dass man eben diese Kompetenz besitzt eine detaillierte Beschreibung seines Zustands abzuliefern (4 Sek.) und halt gleichzeitig diese (...) hm moralische Kompetenz sag ich jetzt mal besitzt um halt zu sagen okay, das ist ein Angebot für mich weil ich jetzt vielleicht nicht die Zeit hab zu einem Arzt zu fahren weil der Arzt jetzt vielleicht auch nicht allzu viel Zeit hat um und Patienten die Wartezeit im Wartezimmer ersparen möchte aber das nutz ich jetzt nicht aus um Missbrauch zu betreiben oder um jetzt zu sagen okay (...) dass ich dieses System irgendwie ausnutz also ich denk mal diese Kompetenz muss auch gegeben sein dass man eben [I: Ja.] zwei diese beiden Kompetenzen hat sag ich mal.

Patient 5

wie würden Sie die Grad dann einschätzen?

P5: (...) Hm (...) ich, glaube fast dass das so also das ich kann mir vorstellen dass so so eine pauschale Aussage in der Realität schwierig ist aber zumindest so dass man ein Grundverständnis von seinem Körper und von von diesen von seinem so von der Beschreibung seines Zustands hat (...) kann ja auf einem auf einem niedrigen Niveau sein [I: Okay.] und dass man auch die die persönliche Fähigkeit hat sein Leiden eben verständlich auch darzulegen auch fernab eines persönlichen Gesprächs sondern beispielsweise übers Telefon

Patient 5

kommt wahrscheinlich dann noch dazu dass man (...) jetzt das haben ja auch viele gerade in meinem Alter nicht die Angst hat vorm Telefonieren und sagt na so (unv.) bin ich jetzt nicht (...) oder so also diese diese soziale Kompetenz muss dann eben auch gegeben sein ich denk mal die sollte sogar ein bisschen oder deutlich höher sein als dieses medizinische Grundverständnis

Patient 5

ein kleines medizinisches Grundverständnis hat aber eine (...) einigermaßen oder gut vorhandene soziale Kompetenz damit man sich eben auch traut ans Telefon zu gehen und diese, dieses Telefonat durchzuführen weil das hab ich jetzt in gerade in in in meinem Jahrgang also bei Leuten in meinem Alter seh ich das auch ganz oft dass die sagen nee ich drück mich vor so Sachen ich mach es wenn es notwendig ist aber [I: Ja.] (...) so gerne telefonieren oder so das ist ja heutzutage nicht mehr.

Patient 5

nehmen wir nur mal zum Beispiel ich kann irgendwie nur nur halbtags arbeiten so und dann muss ja irgendeiner der sozusagen derjenige der was weiß ich (unv.) beim Job irgendwo wo ich da sage hier ich kann nur halbtags arbeiten derjenige muss das natürlich auch irgendwie dann wissen ohne jetzt den gesamten global Zusammenhang irgendwie unbedingt zu verstehen ist ja logisch ne [I: Ja.] (...) dass dass dass man kann nicht von jedem verlangen dass er ein Medizinstudium hat um diese Daten zu verarbeiten ne aber es sollte schon in der Behörde dann jemand da der auch wirklich ein medizinisches Know-how hat und der der das irgendwie im Zweifelsfall also falls es da Probleme gibt das anzuwenden dann auch irgendwie medizinisch das einordnen kann.

Patient 9

würd ich darunter verstehen dass man (...) sich mit seinem Krankheitsbild auseinandersetzt und dann auch (...) weiß wie man (...) sozusagen an (...) Informationen Rezepte (...) Kontakte und Netzwerke kommt die einem dazu dienlich sind zu seinen Gesundheitszustand möglichst gut zu erhalten beziehungsweise so vielleicht sogar zu verbessern. (...) [I: Ja.] Ja und sozusagen im Alltag auch die Dinge die sozusagen das Krankheitsbild mit sich bringen auch umzusetzen sprich was jetzt die (K[1]) betrifft inhalieren Sport Medikamente zu den richtigen Zeiten zu nehmen sich vielleicht aber auch selbst Informationen (räuspert sich) aus eigenem Anschluss heraus abzuholen

Patient 10

selbst Informationen (räuspert sich) aus eigenem Anschluss heraus abzuholen durch Möglichkeiten wie entweder vielleicht direkte Ansprache oder auch die Mitgliedschaft also direkte Ansprache von Ärzten und Fachpersonal beziehungsweise aber auch die Mitgliedschaft in Vereinen wo man ja auch regelmäßig über Veranstaltungen oder auch Newsletter (...) neue Informationen bekommt (...) zu Forschungen in dem Feld zu neuen Medikamenten neuen Behandlungsformen et cetera.

Patient 10

I: Mhm (bejahend) ja und genau diese diese Genauigkeit diese präzise hilft dann auch dabei mit laut laut Sie oder nach Ihrer Einsicht nach Ihrer Sicht um dann auch ja besser mit der Erkrankung umgehen zu können?

P6: Richtig [I: Ja.] ganz ganz genau ja

Patient 6

wenn ich also diese (...) exakten computergesteuerten Diagnosen habe ist das für mich nur die eine Seite der Medaille und die andere Seite der Medaille ist. Also das ist ja etwas was gesteuert ist von der Technik her von von von dem Arzt ja oder von der Ärztin die andere Seite ist (...) was kann der Patient selber tun um mit seiner Krankheit umzugehen und da finde ich da fehlt als oder sehr häufig ich will das jetzt nicht verallgemeinern da fehlt sozusagen als Gegengewicht so dieser (...) großartigen Technologie fehlt eben auch eine eine Beratung wie wie kann der Patient seinen Lebensstil so ordnen dass er (...) den Verlauf der Krankheit oder überhaupt präventiv gegenüber der Krankheit tätig werden kann ne also ich finde Technologie alleine (...) ist deutlich zu wenig sondern Prävention und aktiv werden lassen des Patienten also in seinem häuslichen Umfeld Bewegung Sport und so weiter (...) ist mindestens (...) eben eben so wichtig und vielleicht mangelt es daran (...) dass man hier den den Patienten zu wenig mitgibt was sie wirklich aktiv tun können.

Patient 6

Kompetenz das was der Computer liefert zu verstehen das geht natürlich nur über den über den Arzt der hoffentlich wenn er die Zeit hat es dem Patienten auch wirklich genau genug und differenziert erklärt

Patient 6

wenn der Patient zum Beispiel (...) nicht wirklich deutlich gesagt bekommt wo er sich beraten lassen kann wie er zum Beispiel seine Ernährung managt (...) oder (...) wo er sich hinwenden kann Physiotherapie die die die eben genannten genannten [I: Ja.] Bereiche (...) vielleicht wird man von Seiten des Arztes auch schulterzuckend sagen müssen ja gut ich geb die Beratung zwar aber was der Patient dann macht das ist sein seine ganz alleinige Angelegenheit

Patient 6

eine gewisse ja (...) Kontrolle doch auch anstrebt dass man von einem Patienten eine Bereitschaft (...) einen einen einen einen Nachweis verlangt dass er nun auch tatsächlich etwas was seine Erkrankung tut ich mein er muss ja auch in einem in einem Bereich wo fünfzig Kilometer angesagt ist im Auto darf er auch nicht achtzig fahren also [I: Ja.] das denke ich wäre keine Verletzung seiner Privatsphäre.

Patient 6

Ja also ganz genau richtig und ich denke eine Kompetenz die hat man ja nicht sozusagen in die Wiege gelegt bekommen die muss man irgendwo lernen und [I: Ja.] da denke ich in der Tat ist ein ganz wichtiger Bereich (...) die Schule oder die die die die die Lehrlingsausbildung oder was auch immer also ich persönlich empfinde zum Beispiel in der Schule einen fünfundvierzig Stunden Tag den wir zum Beispiel in Deutschland haben ich weiß nicht wo sonst auch noch und mit unglaublich wenigen Bewegungsangeboten (...) und zwei Turnstunden in der Woche und ansonsten schmerlich SPÄRLICH bemessene Pausen. Ich finde die Kompetenz für seine Gesundheit etwas zu tun die müsste also wirklich deutlich verstärkt in der Schule angelegt werden denn es ist doch vollkommen klar der Schlüssel zu Gesundheit dürfte ja wirklich auch Bewegung sein und wenn ich junge Menschen die ja einen sehr viel größeren Bewegungsdrang haben als ältere wenn wenn die eben unablässig nur auf Stühlen sitzen und nur auf ihren Bildschirm starren sozusagen dann wird diese Kompetenz zum Gesundheitstraining finde ich zumindest (...) doch sehr sehr sehr vernachlässigt.

Patient 6

Also das kann man nicht voraussetzen das sind es sind ja auch Gesetzestexte und und da man ich weiß auch gar nicht wo man das alles. (Wie gesagt?) das muss man ja auch verstehen können also [I: Ja.] das ist ja auch das muss man ja schon bald An_ Anwalt sein (lacht) oder ja.

Patient 1

(...) Ja also man muss auch auf das Körper hören?

P12: Genau.

Patient 12

Mit Sicherheit (lacht) [I: Ja.] das mag gut sein dass die das das haben die mit Sicherheit da stehen (...) nur ich bin immer so also ich persönlich da schock ich immer viele mit ich bin immer so auch was meine Krankheit anbelangt (...) so viel wie nötig so wenig wie möglich darüber zu wissen weil ich [I: Okay.] von der Einstellung her. Also ich mach gerne überall mit das ist auch kein Problem aber das hab ich auch in der Reha gemerkt die sind alle top informiert wo ich dachte okay (lacht) ja und ich fühlte mich eher so wie so ein Grundschulkind in der ersten Klasse die noch alles lernen ungefähr weil, (...) ich es von jeher weil ich es auch damals nicht anders kannte von jeher immer so gesehen habe. Also viele, (...) hab ich auch in der Reha gemerkt viele stellen ihre Erkrankung an erster Stelle und richten ihr Leben danach. Und bei mir ist es genau andersrum. [I: Okay.] (...) Ich führe erst mal mein Leben und bau meine Krankheit irgendwo ein. [I: Okay. (lacht)] (...) Und klar ich merke dann halt wenn ich da mal zu spät inhaliere und meine Therapie nicht mache oder wie auch immer oder zu spät mache dass ich dann schon ein bisschen mal husten muss das merkt man klar aber ich bin immer so der Meinung ich bin hier um zu leben und nicht krank zu sein [I: Ja.] und von daher mag es sein dass es von den Infos vom Patientenregister irgendwo da auf der Homepage mit Sicherheit steht irgendwelche Informationen und wenn man will kann man sich da auch informieren aber, mein Bedarf ist es jetzt ehrlich gesagt nicht so.

Patient 12

Ja also ich weiß immer noch wenn ich dann meine Berichte jetzt bekomme mit den Laborwerten (...) ich schmeiß einmal einen Blick drüber und denke so Arzt hat beim Termin nichts gesagt dass irgendwas schlecht ist oder so und dann ist es halt immer so (...) ich bin zwar vierzig aber trotzdem interessieren sich meine Eltern halt immer noch dafür vor allem meine Mama [I: Ja.] wie so die Werte sind und wie der Stand halt so ist ne (...) und dann geb ich ihr das Schreiben dann und dann guckt sie selber rauf ja hast du schon mal geguckt und der und der Wert ist erhöht und der und der Wert erhöht ist erhöht ich so Mama ich so wenn irgendwas Schlimmes wäre würde der Arzt mir das sagen. [I: Okay ja.] (...) So und dann heft ich das Ding ab und dann ist gut. (...) Also von daher (...) ich weiß selber wie ich mich fühle und das reicht mir und ja also man es ist. Damit hängt auch viel (...) zusammen wie man sich selber fühlt also in der Reha war eine Szene war fand ich echt krass dass die eine die hatte nur noch zwanzig prozentiges Volumen (...) die hat sich aber gegen eine Transplantation entschieden aber ist halt ihre Entscheidung gewesen (...) und die sagte an dem einen Tag oh sagt sie ich hab heute einen guten Tag ich versuche mal über die Treppe (...) in den ersten Stock zu kommen. (...) So. (...) Und dann haben wir gesagt ja wir gehen mit mir ja aber ich bin doch viel zu langsam wieso wir passen uns deinem Tempo an so haben wir auch gemacht und dann war eine dabei (...) die hatte ein Volumen von dreißig bis vierzig Prozent also quasi fast doppelt so viel (...) die hat gesagt ich bin krank ich fahr mit Fahrstuhl. (...) Und das ist die Einstellungssache [I: Ja.] ich finde viel wenn man eine schlechte Einstellung hat oder so weiter ist man auch kränker als wenn man eine positive Einstellung hat. [I: Ja.] (...) Und das ist halt einfach auch so ein bisschen Bauchgefühl Körpergefühl (...) und so weiter und das gehört auch viel da mit zu finde ich und von daher.

Patient 12

Genau. [I: Ja.] (...) Also ich will jetzt nicht sagen wenn ich jetzt wirklich mal wieder beim Kontrolltermin bin und meine Werte dann von der Lungenfunktion ein bisschen runtersacken zieht mich das auch im ersten Moment ein bisschen runter [I: Ja.] aber ich denke ich so mir geht es immer noch gut klar sind die Werte jetzt vielleicht ein bisschen schlecht aber mein Gott dann liegt es vielleicht am Tag oder an meiner momentanen Verfassung und so weiter also ich war ich bin gespannt ich muss jetzt Ende Oktober wieder hin (...) ich war im muss ich überlegen im Sommer (...) und war davor im A,pril beziehungsweise im Januar da genau in XXX zumindestens (...) da sind meine Werte um fünf Prozent gestiegen. (...) Von daher. (...) Also wieder besser geworden also [I: (unv.) es ist echt viel die Einstellung die es macht im Endeffekt

Patient 12

Hm na ja dass man aus selbstermittelten Daten oder Daten die einem unmittelbar mitgeteilt werden von irgendeinem Gerät dass man daraus selber Behandlungsentscheidungen ableiten kann. Und das ist gerade beim (K[3]) ist das ja dann auch eine ganz also das ist gehört ja zum alltäglichen Leben ich bekomme von dem CGM halt angezeigt die Verlaufskurve der letzten Stunden kann mir überlegen was möchte ich jetzt essen und darauf aufbauend bestimme ich dann wie viel (Mk[19]) ich mir injiziere beziehungsweise wäre ja auch möglich wenn ich eine Pumpe zum Beispiel tragen würde dann würde ich ja auch darauf aufbauend die Entscheidung treffen was ich dann jetzt mit der Pumpe veranstalte

Patient 13

Genau weil so habe ich wenigstens auf dem Rezept die Hand_ also die Handelsnamen der Präparate Schwarz auf Weiß wenn ich was Neues verschrieben bekomme kann ich auch

selber schon mal recherchieren was ist das eigentlich [I: Ja.] und das wird beim E-Rezept dann ganz anders sein weil dann hab ich das dann auf auf dem Tisch stehen und erst dann kann ich mich damit beschäftigen.

Patient 13

Für mich also dass man sich selbst mit seiner Krankheit gut auskennt. (...) Und dass man so ein (...) so ein medizinisches kleines Grundwissen hat. [I: Okay.] (...) Dass man auch Sachen vielleicht ein bisschen selber interpretieren kann (...) vielleicht auch die Kompetenz hat die richtigen Fragen zu stellen

Patient 3

Ja genau also so also also einfach so eine so eine Kompetenz also so ein wie so ein also ein mündiger Patient

Patient 3

(räuspert sich) Ja eigentlich immer seine Medikamente auch zu kennen. (...) Was das ist und für was man das einnimmt. (6 Sek.) Und man das das Wissen (...) kriegt man halt, von Ärzten und [I: Ja.] medizinischem Personal das einem das erklärt und schult also [I: Ja. (unv.) (...) wenn man jetzt natürlich einen Arzt hat der keine Zeit hat oder einen Arzt hat der kein Interesse hat (...) ist es halt schwierig. (...) [I: Ja.] Na gut jetzt übers Internet kann man auch viel lernen aber da muss man natürlich auch wissen welche Quelle ist jetzt gut und welche ist jetzt schlecht.

Patient 3

Hm also wie gesagt mir fällt jetzt nur diese (...) diese Schulung ein. (...) Dass man das lernt irgendwie. [P3: Und und.] (...) Ansonsten kommt auch drauf an ich weiß nicht ob man selber dann auf diese Patientenakte zugreifen kann. (...) Das könnte also wenn man das natürlich kann ist das natürlich auch schwierig weil ja also ich kenn ja so auch Arztbriefe und so ein Zeug (...) also da wenn man das nicht studiert hat versteht man das auch oft nicht. [I: Ja.] (...) Ich fang auch schon gar nicht mehr an zu googeln [I: Okay.] ich frag dann ich ich markier mir das und frag den Arzt aber das macht wahrscheinlich auch nicht jeder. [I: Ja.] (...) Aber das, ja weiß ich jetzt auch nicht also (...) wenn man da nicht auch selber drauf zugreifen kann das passt es ja. (lacht) [I: Ja okay.] Dann kommen keine Verunsicherungen

Patient 3

Da hab ich jetzt nicht direkt also (...) ich glaub das ist auch wirklich schwierig. (4 Sek.) [I: Ja.] Also da wenn man sich jetzt nicht so auskennt da selber zu entscheiden wer was sehen kann (...) also die relevanten Informationen rauszu_ also zu wissen, ist sehr schwer. [I: Ja.] Wenn man jetzt nicht zufällig im Medizinbereich arbeitet.

Patient 3

aber auch der Benutzer selbst also wenn [I: Okay ja.] man sich nicht damit beschäftigt (...) ist halt dann immer schwierig. (...) Ne ich mein es gibt ja auch also es gibt auch genug Leute die (...) so so was halt auch gar nicht so genau wissen möchten [I: Ja.] (...) also damit muss man natürlich auf, also auf die Gruppe muss natürlich auch eingehen wenn jemand sagt ich möcht gar nicht so genau wissen was in dem Befund steht ich will es auch gar nicht so genau wissen was da kaputt ist ich (...) ich will das gar nicht so genau wissen ich will nur wissen wann ich welche Tabletten nehmen muss und fertig.

Patient 3

Na ich denk das ist einfach die persönliche Einstellung. (...) Also ich ich bin jetzt jemand ich will immer alles wissen ne also ich frag ja dann auch Dinge nicht unbedingt jetzt mit dem Befund zu tun haben und dann fällt mir wieder irgendwas ein wie wie geht denn das und das was ist denn da und da ich will immer alles genau wissen ich will auch immer wissen wenn ich operiert werde wie genau funktioniert die OP. (lacht) (...) Am besten noch mit Video und so ein Zeug [I: Okay. (lacht)] ne. (lacht) [I: Ja.] Aber ich hab halt auch eine Freundin die sagt oh ich will das gar nicht so genau wissen je je mehr ich weiß desto mehr Angst krieg ich. [I: Ja.] (...) Und bei mir ist natürlich genau umgekehrt. [I: Okay ja.] (...) Und es ist beides in Ordnung.

Patient 3

II. Qualitative data from the online survey, including free-text responses

**[ND05]** Skala<br>**"Uns interessiert im Folgenden, ob bestimmte Umstände die Nutzung von digitalen Medien und/oder Angeboten grundsätzlich für Sie erschweren."**
**[ND06]** Texteingabe offen<br>**Sonstiges (bzgl. ND05)**<br>**ND06_01** Sonstige Schwierigkeiten, und zwar<br>Offene Texteingabe

Sonstiges (ND06): Sonstige Schwierigkeiten, und zwar
...da seit diesem Jahr der überregionaler Anbieter […] für Telemeditin fehlt. Andere Anbieter stellen keine Überweisungen aus, die man gerade für die Immunologie braucht.
...dee Registrierungs-, Verifizierungs- und Authentifizierungsprozess zu umständlich ist
...sie für die Nutzung die Preisgabe personenbezogener Daten, insb. der Telefonnummer, verlangen, ohne dass diese eigentlich für den Betrieb erforderlich sind.
Aktualisierung der Daten dauert öfters viele Monate
An der Gestaltung mancher Apps und Webseiten ist noch viel nach oben
Anbieter und seine Motivation für dieses Angebot unklar bleiben
Anschaffung der Geräte wie Smartphone, Laptop, PC, Drucker, Webcam, Headset, etc. sind schwierig, wenn man durch die Erkrankung von Grundsicherung lebt.
Apps von Krankenkassen haben einen so gesicherten Zugang, dass es kompliziert wird, wenn man mal sein Passwort vergisst.
aufwendige Authentisierungsverfahren, z.B. PIN-Versand der KK nur per aufwendigem Postident
Aversion gegen Smartphone wegen des kleinen Bildschirms, geringerer Suchmöglichkeiten, schlechten Tastatur. Notebook oder PC viel besser.
Bei Problemen keine Hilfe und Ansprache
bei Smartphone und Computer keine Angabe, da ich damit täglich problemlos lebe und keine der vorgegebenen Antworten m.E. nach passt
Bin 75 Jahre und mit dem Internet nicht so vertraut
Chatfunktionen sind oft nicht barrierefrei.
Datenschutz und zu aufwändig wenn es mich nicht unmittelbar betrifft
Datensicherheit und vermehrte Hackerangriffe (Smartphone und PC)
der Zugang (Code, mehrere Stufen etc,) schwierog ist ; wie in einer "Schleife"
Die Fülle an Angeboten zu unübersichtlich sind.
Die Sachen interessieren mich nicht da ich nicht nachvollziehen kann welche Quellen benutzt werden
die Spreu vom Weizen bzw. seriöse Seiten von unseriösen zu unterscheiden
Die Vertrauenswürdigkeit ist nicht immer gut einzuschätzen (Quelle)
diese Antworten variieren sehr stark von Angebot zu Angebot.

digitale Angebote sind ausbaufähig
Digitale Informationsflut überfordert mich im Alltag, da sie kein einheitliches Konzept sind.
Ein Smartphone funktioniert bei mir nur begrenzt, ich wohne in einem Funkloch.
Eine App ständig etwas von mir will
eingeschränktes Zeitbudget
EMF-Sensibilität (Elektromagnetfeld)
englisch
Enthalten überwiegen vielerlei Informationen die nicht zu den Themen zu treffen
Es gibt leider etliche schlecht aufgebaute Websites
Es ist schwierig einen "single point of truth" zu finden mit möglichst umfangreichen Informationen die sowohl die Nutzung als "Anfänger" als auch "Fortgeschrittenen" ermöglicht.
Fach chinesisch verständlich übersetzen
Fehler im Design des Benutzerinterfaces (UI)
Fremdsprachen ohne aut. Übersetzungsmögl.
Hörbehinderung : deshalb nutze ich kaum Videos
Ich ein Smartphone aufgrund meines Handicaps nicht bedienen kann
Ich habe ein Mobiltelefon, aber mit Absicht kein Smartphone
ich habe kein Smartphone und möchte mir auch nie wieder eines zulegen wg. Belästigung.
ich habe Zugang zum Internet nur über W-Lan - gesicherter Datenaustausch außer Haus?
Ich kann/darf keine eigenen Infos ergänzen, würde das aber gern machen
ich lebe ohne WLAN und nutze nur die mobilen Daten meines Smartphones für den Zugang zum Internet, da führt die längere Nutzung dann schnell zu Kopfschmerzen.
Ich möchte besser erkennen können, welche Informationen als wissenschaftlich gesichert bewertet sind.
Ich möchte das nicht.
Ich nicht abschätzen kann, welche Konsequenzen sich daraus ergeben
ich nicht weiss ob die Quelle verlässlich ist, es gibt heutzutage viele "Wunderheiler"
ich selbst habe überhaupt keine Ahnung von diesen Medien; mein Mann füllt für mich den Fragebogen aus.
ich stelle mich nicht nackt auf einen Marktplatz im web
ich technisch immer auf den neuesten Stand sein muss, Soft- und Hardware
in der regel keine
Inhalte oft mangelhaft
Keine
Keine Übersetzung für Gebärdensprache möglich / Videosprechstunde mit Dolmetschende nicht möglich
Komplexes Anmeldeverfahren
Komplizierte Krankenkasse Sprache , fehlende Apps, Verknüpfungen
Komplizierter Aufbau, schlechter Support
Konten- und Passwortmanagement

Kostenfreiheit nicht gewährleistet ist /Datenübertragung an Dritte/ kostenpflichtige Dienste in Anspruch genommen werden müssen
kostenpflichtige Angebote, wenn der nachhaltige Nutzen unklar ist.
langes Sitzen vorm Computerbildschirm wegen Augen und Rücken
Manchmal ist es schwierig zu unterscheiden, ob Informationen gut recheriert sind, von wem sie kommen und ob man vertrauen kann
mein Alter >65
meine Hausärztin nicht gut digitalisiert ist
muss möglichst mit Sprachsteuerung sein, wegen Sehbehinderung […]
neue Programme, neue Software
nicht funktionierende Apps wie die EPA
Oft zu kleine Schrift
Orientierung bei auftretenden Pannen, nötige Abhilfe
Schlechte Gliederung, viel Werbung, keine gute Suchoption
Schlechte Interneverbindung
sie Apps sind - Datenschutzproblem wegen des Zugriffs auf viele anderen Dinge auf dem Handy!!
sie Dinge versprechen, die sie nicht halten (können)
siehe Frage 1 und Ihre Textgröße
Smartphone: zu kleine Tasten, zu mühsam bei Texteingabe, Korrekturen selten möglich
Sortieren der vielen Informationen, welche sind fachlich basiert und was sind nur Meinungen.
Suchfunktion enthält nicht alle Wörter, daher geringe Trefferquote
Suchmaschine zeigt zu viel Werbung und nicht immer das, was ich eigentlich wollte
Teilweise schwer zu den gewünschten Inhalten vorzudringen bzw. Rückmeldung an die richtige Stelle anzubringen. Manchmal scheitert es an der Verwirrung bzw. Widersprüchlichkeit bei Passwort u.ä. -Benutzung . D.h. teilweise viel zu kompliziert, schnell eine bestimmte Info zu bekommen !
überhaupt zu wissen, wo man an die richtigen Fachinformationen kommt - teilweise zu viel um unterscheiden zu können, was man grad für sich benötigt.
Unübersichtliche Seiten
unübersichtliche Webseiten mit Werbung und Cookie Feldern
Verlinkungen in den Angeboten selbst, welche technisch nicht einwandfrei funktionieren
Viele Ärzte etc. nutzen keine digitalen Medien, daher kaum digitale Kommunikation möglich.
Viele Quellen von einandern abschreiben und somit den Informationsumfang schmällern
Wegen der vielen links und Werbung braucht man oft lange, um das Gesuchte zu finden
wenn das Angebot nicht komplett über Siri gesteuert werden kann
Wenn das Internet nicht funktioniert
wenn es sich anfühlt als ob das Produkt aus den 90ern kommt
Wenn falsche Befunde verbreitet werden, da muss man als Patient Rechte bekommen

wenn mir nicht klar ist, welche Motivation die Betreiber der Plattform für ihren Auftritt haben (versteckte Werbung)
zu kleine Schriftarten, zu viele Informationen auf einer Website, wenig Erfahrung mit Möglichkeiten von Smartphones, nur altes Smartphone mit begrenztem Speicher- und Arbeistspeicher vorhanden, die wirklich tiefergehenden Texte, Fach- und Forschungsliteratur sind oft in englischer Sprache und enthalten für einen Laien schwierige Fachwörter
Zuviel digitale Bevormundung und Zensur.

[ND07] Texteingabe offen

"Gibt es noch etwas, das Sie uns zum Thema „Nutzung digitaler Medien und Anwendungen" mitteilen möchten?"

Offene Texteingabe

Anmerkungen [ND07]:
- Die Informationen vielleicht nicht der Warheit   entsprechen. -FakeNews<br>- viel Werbung/Anzeigen die stören
1verkehrter Klick schon fülle ich ihre Fragen ein zweites mai aus ?
Als älterer Mensch, müßte man besser geschult sein um mit den Medien umzugehen
Alte kranke Menschen sind damit überfordert
Angebot an Apps zu Medikamentenplan und einnahme-Erinnerung unübersichtlich. Eine sichere und kostengünstige "verschreibungsfähige" App wäre hilfreich.
Angebote sollten gut durchdacht, geprüft, sicher und von allen Seiten gleich gut nutzbar sein
Antworten sind oft entweder zu allgemein, ähnlich wie in Broschüren. Oder sehr viele Informationen mit weiteren medizinischen Fachausdrücken die dann auch erst nachgelesen werden müssen.<br>Fehldiagnosen vorprogrammiert. Wenn eine Diagnose da ist, aber sehr gut zum nachlesen und nach Forschen.
Ärzte und ähnliche dürfen keine E-mail verschcken. Wäre so eine Erleichterung
Aufgrund der Vielzahl an Informationen, besteht die Gefahr sich zu verzetteln und am Ende zu keiner guten Entscheidungsfindung gelangt. Digitale Medien können dazu beitragen, dass Menschen verlernen auf ihren Körper und ihr Bauchgefühl zu hören, wahrzunehmen was ist und herauszufinden, was gut für sie ist.
Aus meiner Sicht gibt es zu wenig Angebote insgesamt. Wir sind bei der Digitalisierung in Deutschland sehr weit zurück hinter anderen Ländern.
Bei der fortschreitenden Digitalisierung sollten alte Menschen und Menschen mit Beeinträchtigungen immer bedacht werden!
Bei uns gibt es zu wenig due Möglichkeit der Digitalusierung wie e-Rezept, e-Akte, E-Mails statt Vor-Ort Besuch z. B. für Berichte, Rezepte
Beim Design der Webseiten sollte auch auf Sehbehinderte Rücksicht genommen werden. Z.B. mit Vorlesefunktionen, kontrastreiche Schrift, übersichtlicher Aufbau.
Beispiel: Terminvereinbarung mit einer Facharztpraxis.<br>Meistens geht das über eine zentrale Serviceeinrichtung (z.B.:[…])<br>Die Praxen sollten direkt erreichbar sein.

Benutzerkonto, Passwort, Mehrfach-Authentifizierung, Time-Out, zuletzt eingewählt - rausgeflogen
Besitze kein Smart- Phone
Bisher gab es wenig Möglichkeiten für mich digitale Angebote zu nutzen, da meine behandelnde Ärzte keine solchen Angebote zumindest direkt unterstützen.
Bzgl.Apps: Ich bevorzuge Apps von Hochschulen, da ich hier einen Forschungsbeitrag leisen kann und ich meine Daten für gesichert halte.
Da die Daten anonym sind, und für mich nicht nachprüfbar, habe ich ich zu den angebotenen Inhalten kein Vertrauen
Da fehlt das persönliche menschliche, man erreicht kaum mehr einen Menschen persönlich
Da ich bereits 82 jahre bin, tue ich mich schon mit komplizierter digitaler Nutzun schwer.
Dänemark ist 99% digital
Das Netz ist nun mal unsicher und daher mit Sicherheit, wenn möglich zu nutzen.
Das Angebot an sich ist viel zu gering. Ich würde gerne mehr nutzen.
Das der digitale Hype ein Problem darstellt, dass diese Vision mit Macht,- Datenausnutzung und die fortschreitende KI Bots zu einen unsicheren Platz wird. Das nicht nur Patienteninformationen missbraucht werden, sondern auch Umweltprobleme (CO2 Emossionen) verstärkt werden.
Das Thema Datenschutz ist für mich immer ein Problem
Das Thema Sicherheit und Datenschutz finde ich sehr wichtig
Das war schon ei wenig schwierig.
Datenmissbrauch ist ein Problem,unübersichtlich
Datensicherheit - sehr hoher Stellenwert
Der persönliche Kontakt ist wichtig.<br>Die Digitalsierung im Gesundheitssystem macht mir Angst. Pflegeroboter statt Mensche. Der durchsichtige Patient. Sie arbeiten hier in einem Bereich, der den Menschen schadet ohne es zu wissen. Die Teilnehmer an der Befragung sind ja bereits nur PC affine Menschen. Dies birgt eine Fehlbewertung die den Rest der Bevölkerung missachtet. (Nicht achtet/verachtet nicht inkludiert)<br>Falsches Ergebnis!
Der Schutz der persönlichen medizinischen Daten darf niemals in falsche Hände geraten. Nur wie soll das gehen? Es bräuchte dazu eine staatliche Unterstützungs-Einruchtung insbesondere für Personen, die nicht in der Lage sind, mit digitalen Daten umzugehen (Alter, Behinderung, mangelnde Kompetenz).
Der Zugang zu Studien ist durch das Bezahlsystem erschwert für nicht Mediziner aber ebenso interessant und wertvoll für Patienten. Hier wünsche ich mir einen kostenlosen Zugang.
Die Daten sind sensibel, da von mir eine Überprüfung der Personen mit Datenzugriff nicht möglich ist, verliere ich den Überblick, wer was von mir kennt. Hier besteht für mich die Gefahr von dem "gläsernen Menschen". Datenschutz wird zwar mit Erklärungen zugesagt, in der Realität ist es jedoch nur noch ein Haftunsschutz für den Anbieter nicht für den Nutzer.
Die Einführung geht viel zu schleppend und ehe ich z.B. ePA oder E-Rezept nutzen kann, habe ich teilweise schon einige angeeignete Fertigkeiten wieder vergessen und muss von vorn anfangen.

Die eurteilung der Barierren ist abh. vom Angegebot. Sprachliche Barrieren bestehen bei Whatsapp nicht, selbst bei internationaler Kommunikation gibt es automatische Übersetzungen aber bei der Recherche von Fachstudien in pubnet werden Grenzen auf der ersten Seite erreicht. Insofern ist die vorhergehende Bewertung ehre eine durchschnichtliche Beantwortung der Gesamtangebote.
Die Frage der Seriösität der Angebote. Wer testet die Angebote im Vorfeld?
Die Fülle digitaler Medien erschweren oft eine Übersicht und konsequente Verfolgung von Sachverhalten.<br>Bestimmte Medien, die von manchen Netzwerkgruppen benutzt werden, sind umständlich und verringern somit die Attraktivität für mich (z.B. schriftlicher Chat)
Die GKV und PKV sollten ihre Angebote ausbauen und auch direkte Interaktion mit dem Patienten durchführen (Überwachung?)
Die initiale Einrichtung ist für die meisten Menschen komplex bis abschreckend, s. E-Rezept und damit verbundene Aktivierung der PIN der KK per Postident. Für nicht mit Technik vertraute Personen dann am Ende eine unüberwindbare Hürde.
Die Kanäle, über die Informationen eingeholt werden aber auch eingehen werden immer vielfältiger, der Überblick wird immer schwieriger
Die Krankenkasse keine Hörsysteme mit beispielsweise Bluetooth finanziert.
Die Möglichkeit von Podcasts ist mir nicht geläufig, da ich diese Option noch nie genutzt habe.
Die Nutzung sollte durch einheitliche Icons oder Piktogramme vereinfacht sein / Wiedererkennung erleichtern bei verschiedenen Anbietern.
Die Nutzung sollte einfach und intuitiv sein. Darüber hinaus sollte die Bedienung zeitlich nicht zu lange dauern.
Die Nutzung sollte möglichst mit Sprachsteuerung oder mindestens in Vorlesefunktion möglich sein.
Die Nutzung von Digitalen Anwendungen kann ich nicht nachvollziehen. Irgendwelche Technik KI entscheidet über Leben und Tot. Nicht mit mir.
Die Nutzung von dogitalen Medien und Anwendungen scheitert bei mir auch daran, dass ich unzureichend darüber informiert werde und sie mir oftmals weitgehend unbekannt sind. Zudem fehlt es nicht selten an etwaigen "Bedienungsanleitungen", beispielsweise in Form von Videos oder einfach verständlichen Anweisungen. Und auch die Herkunft der Informationen bleibt oftmals ungeklärt, weshalb ich ein grundständiges Misstrauen habe, wenn ich nicht weiß, wer die Auskünfte verfasst hat.
Die PIN und PUK bei der ePA sind sehr kompliziert
Die Quellen sind oft nicht ersichtlich. Sorge, dass ich auf PR von Pharmaunternehmen hereinfalle wie z.B. im Falle von Borreliose bei Pfizer. Auch das Impressum hiulft oft nicht weiter, weil die Verantwortung in unbekannte Agenturen mit Fantasienamen ausgerlagert wird.
Die Sicherheit der Daten wäre mir wichtig.
Die Suchmaschinen sollten ähnlich Chat GPT auch auf umgangssprachliche Fragen Antworten finden<br>Die Suchergebnisse ohne fehlende Fachbegriffe sind uneindeutig und oft falsch

Die Telemedizin und die Online Psychotherapie muss weiter ausgebaut werden und darf zeitlich nicht beschränkt sein.
Die vielen Tracking- und Cooky-Anforderungen schrecken mich ab. Die "Abschaltoption" ist oft sehr aufwändig, weil die Funktion "alles Ablehnen" nicht eingerichtet ist.
Digitale Angebote/Inhalte sind wie schon erwähnt nicht einheitlich aufgebaut, zudem gibt es keine Schnittstelle und die Vernetzung ist nicht da. Damit ist es reine GLÜCKSACHE, ob wichtige und relevante Angebote/Inhalte gefunden werden.<br><br>Zudem ist Werbung, die z.B. in einer Anzeige geschaltet werden, irreführend.
E-Akte würde ich nutzen, soll ja kommen. E-Rezept klingt auch interessant. Ansonsten ist es manchmal schwer - rauszufinden, wo bekomme ich nun wirklich gute Informationen her und musss ich teilinfos selbst zusammensuchen. Vorallem wenn die eigentliche Diagnosse fehlt und die Ärzte selbst keine Ahnung haben.
Ein kostenfreier Zugang zu Cloud-basierten Ablage- und Austauschplattformen für […] wäre förderlich. Gruppen, die kassenübergreifend geförderten Gruppen, kämen dafür z.B. infrage.
Ein persönliches Gespräch ziehe ich immer vor einer digitalen Anwendung vor. Dann können Missverständisse ausgeräumt werden. Fragen werden korrekter beantwortet. Emotionen spielen auch eine Rolle.
el. Patientenankte nutzbar machen wäre eine echte Hilfe für Menschen mit "interdisziplinären" Krankheiten.<br>Leider geht es hier in D seit vielen Jahren nicht voran
Elektronische Patienten akte ist noch wenig bekannt, da fehlt Personal zur umsetzung
Elektronische Patientenakte: Komplizierter Zugang. Ich hätte gern eine, aber bei dem Antragswust dafür - nee, da warte ich noch.
Endlich kommen die elektronische Patientenakte und E-Rezepte. Für Privatpatienten habe ich allerdings noch nichts davon gehört!
Es gibt mittlerweile Ärzte, die fast ausschließlich auf digitale Betreuung setzen. Digitale Betreuung sollte die persönliche Betreeuuung jedoch nicht vollständig ersetzen.
es gibt noch sehr wenige online Angebote, Akte, Apotheke, Rezept
es gibt zu wenige - oder kaum - elektronische Angebote, individuelle wie zB ePA, eRezept für Privatpatienten
Es ist hilfreich, wenn Symposien und Mitgliederversammlungen von Selbsthilfe-Organisationen hybrid stattfinden, da oftmals die Anreise zu diesen viel Zeit und Geld kostet und man sie sonst verpassen würde.<br>Auch online-Sprechstunden von Ärzten sind äußerst hilfreich. Wartezeiten können so vermieden bzw. optimal genutzt werden.
es ist sehr kompliziert die Patientenakte einzurichten. Zu viele einzelne Schritte und es kann nicht in einem Verarbeitungsschritt erledigt werden.
es könnte die Gefahr bestehen, dass in naher Zukunft ohne Smartphone nichts mehr erreichbar ist.
Es liegt nicht immer eine zum Ausdruck geeignete Version vor. Das ist zum Teil unschön.
Es müsste Informationen bzw. Seminare zur digitalen Nutzung, auf die jeweilige Altersgruppe abgestimmt, geben.
Es sollte eine einheitliche, einfache und verständliche App geben

Es sollten immer gute Suchefunktionen für das jeweilige Medium geben. Und auch übersichtliche Inhaltsverzeichnisse. Besonders sollte in Foren sehr früh immer sinnvolle Kategorien a gelegt werden, in denen Themen bearbeitet werden. Finde das Forum der […] Selbsthilfegruppe dazu sehr gut. Gibt aber auch noch nicht soviele Einträge dort...
Es wird eine deutliche Veränderung der Apps und des Informationsangebots im Internet geben, wenn KI stärker Einzug gehalten hat. Ev. wird es dann leichter, weil die Bedienung über Sprachinterface hinzukommt (funktionale und totale Analphabeten) und der Benutzer nachfragen kann bzw. auch das Sprachniveau wählen kann.
Fehlende Informationen was es alles für Angebote und Möglichkeiten gibt
Für ältere Menschen ist dieses zu schwierig da diese kein pc oder ähnlich haben
Für die Community der Gehörlosen: Untertitel ist eine Barriere weniger. Noch besser, wenn es Gebärdensprachdolmetscher (und wenn mal die Technik der Gebärdensprachdolmetscher-Avatare so weit einsatzbar wäre) gäbe - analog für die Blinden, wo der Inhalt der Webseite vorgelesen wird.
Für mich als chronisch kranke ist es eher unrelevand, da ich einen festen Ansprechpartner (Arzt der mich kennt) brauche
Für mich ist ein persönliches Gespräch mit einem Arzt nicht durch die beste Telemedizin ersetzbar!
Für mich wäre wichtig, dass es immer auch eine analoge Möglichkeit gibt, die man statt der digitalen nutzen kann. Gerade für Menschen, die nicht so computer- und digitalaffin sind, wäre das sehr erleichternd
Für taube Senior*innen sehen wir grosse Schwierigkeiten. Zum Beispiel die zweifache Authentifizierung bei den Krankenversicherungen ist sehr kompliziert. Videosprechstunden mit Zuschaltung von Gebärdensprachdolmetschende sind nicht möglich<br>Telefonate über Dolmetscher-Relay-Dienste sind aufgrund Datenschutzbestimmungen nicht möglich.
Für vieles zu viele verschiedene Apps oder funktioniert nicht einwandfrei
Gendersprache führt generell zu Abbruch jeder Befragung!!
Generell gilt: Vieles zu kompliziert, was viel einfacher gehen könnte. Zu wenig Hinweise auf Sprachsteuerung und ChatBot Ki
Gerne über das Smartphone Täglicher Gebrauch und wissen mit Umgang Computer eher schwieriger und unpraktischer
Gute Apps für regelmäßige Übungen und Checks durch Regio-/Erfagruppen und Phyio angenommen werden
Guter Datenschutz ist Grundvoraussetzung
Hier auf dem Land noch nicht gängig. Oft überfordert mit Handhabung
Hierdurch kann es zu einem Verlust der menschlichen Kompetenz kommen, Mensch zu sein, zu kommunizieren, aus Verhaltenssituationen zu lernen usw. Ein weiteres Absinken des durchschnittlichen IQ (Intelligenzquotienten) sowie der Verlass auf die künstliche Intelligenz sind ein gefährlicher, bedenklicher Trend.
Hilfreich ist die Beschreibung der einzelnen Bedienungsschritte und ein Hindweis, ob Angaben zwingend erforderlich sind.

Hin und wieder stehen Angebote (z.B. Rehas, Apps) nur gesetzlich Versicherten zur Verfügung. Als Privatversicherter ist man sich öfter mal selber überlassen oder kann die Angebote nicht nutzen.
ich befürchte, dass viele Praxen nicht den erforderlichen Datenschutz anwenden bzw. meine Daten bei gewerblichen Rechenzentren landen die den Datenschutz ebenfalls nicht umsetzen. Meiner Meinung nach müsssen diese sensiblen Daten unter stattlicher oder behördlicher Aufsicht gespeichert werden.
Ich bevorzuge Tastatur und Notebook:<br>- Mehrere Fenster gleichzeitig öffnen und Daten vergleichen oder mit Copy+Paste arbeiten<br>- Größere Bildfläche, mehrere Bildschirme möglich = mehr Informationen und besserer Informationsfluss<br>Digitale Medien sollten mit persönlichen Daten und mit anderen Anwendungen gut verknüpfbar sein = weniger Aufwand z. B. beim Ausfüllen von Formularen, besserer Informationsfluss
Ich bin ein starker Befürworter der EPA usw. Leider kommt das Projekt einfach nicht in die Gänge. Weil es einfach zu kompliziert in der Umsetzung ist.
Ich bin generell dagegen digitale Dienste statt einem Arzt zu nutzen. und schon gar nix was mit KI o.ä. zu tun hat
Ich bin immer vorsichtig, wenn nicht gleich zu Anfang steht welche Kosten auf mich zukommen.<br>Auch möchte ich wissen, ob medizinische Fachleute beraten oder KI.
Ich finde es schade, dass Krankenkassen hilfreiche Gesundheitsapps zum Teil noch finanzieren.
Ich finde es sehr hilfreich, wenn bei einem Angebot oder einer Befragung gleich der Hinweis ist, an wenn man sich bei Problemen wenden kann (wie bei dieser Befragung) Danke.
Ich hab Schwierigkeiten Angebote zu nutzen, die ihren Nutzen durch schlechten oder fehlenden Aufbau verfehlen, Bsp. die […], da man dort jeden Tag direkt eintragen muss, nachtragen ist keine Option. Wenn man einen Tag vergisst, wird also die Statistik verzerrt...
Ich habe erst heute nach einer App gesucht, in der ich Symptome eintragen kann, wann ich welches Medikament in welcher Dosis genommen habe, wann wie mein Stuhlgang war, am besten kann ich dort Laborergebnisse hinterlegen, das wär mega
Ich habe lieber etwas in der Hand, als nur auf den Bildschirm zu schauen.
Ich halte KI gesteuerte Symptomerfassung und Diagnosehilfe für Ärzte und Ärztinnen unerlässlich, da diese keine Zeit mehr für Anamnese und Diagnostik haben und zu wenig Kenntnisse in Bezug auf die Physiologie des Menschen, Stoffwechselprozesse und endokrinologische Vorgänge haben.
Ich hätte gerne beim eRezept die Option "ja" angekreuzt, […]. Allerdings wurde das eRezept bisher ja noch nicht eingeführt und ist nicht in allen Praxen möglich.
Ich kenne mich ganz gut aus, allerdings bringt es nicht viel wenn Apotheken keine eRezepte akzeptieren, Ambulanzen den Medikamentenplan nicht von der Versicherungskarte abrufen können.
Ich nutze keine digitalen Medien, wenn ich mich verbindlich registrieren muss. Da bin ich vorsichtig.
Ich nutze mein I Phone, bin aber mit der Nutzung nicht voll nutzungsfähig, weil ich viele Dinge nicht weiß wie sie funktinieren
Ich nutze täglich mehr als 7 Stunden digitale Medien

Ich orientiere mich gerne über medizinische Themen an dem Deutschen Ärzteblatt. Hier findet man Publikationen von Fachärzt/innen etc.<br>Leider ist der Zugang nicht für die Allgemeine Nutzung freigeschaltet...Also man muss "Kollege/gin sein um den Text freigeschaltet zu bekommen....
Ich persönlich möchte mehr digitale Anwendungen nutzen, finde es aber wichtig auch eine Alternative für Menschen zu haben die das nicht möchten / können
Ich schreibe mein […]protokoll mit einem Programm, das mein Mann erstellt hat. Leider stand kein fertiges Programm dafür zur Verfügung.
Ich sehe eine große Chance in der Weiterentwicklung von digitalen Patienten-Apps`s - z.b im Bereich der […].
Ich stehe z.B. den Medikamentenplänen, die andere für mich erstellen kritisch gegenüber. Da viele ÄrztInnen meine seltenen Medikamente gar nicht kennen und ihnen auch der komplette Überblick fehlt wegen meines seltenen Erkrankungs-Potpourri. Diesen erstelle ich grundsätzlich nur selbst ergänzend mit den wichtigsten Diagnosen, Allergien und sonstigem Wichtigem. Auch bei Rezepten gibt es immer wieder Probleme wegen aut idem Produkten oder Zuzahlungsbefreiung. Daher nur mit einer durchgehenden Profi-Kette, die ich leider noch nirgends angetroffen habe. Und in Arztberichten steht auch oft sehr großer falscher Mist, den man korrigieren lassen muss.
Ich vermisse oft einen logischen, übersichtlichen Aufbau.<br>Dafür finde ich eher Symbole wie Smilys etc.- was m.E.<br>möglicherweise mit dem Alter der Progammierer zu<br>tun hat. Diese können sich evt. nicht auf das Alter von<br>Senioren einstellen und berücksichtigen es nicht genügend.<br>Es existiert heutzutage eine "Spielgesellschaft", die Symbole<br>verwendet. Damit können Senioren kaum etwas anfangen.<br>Möglicher Grund : früher auf der Schreibmaschinentastatur<br>konnten keine Symbole dargestellt werden.<br>Meine dringende Bitte: wenn Sie Senioren erreichen wollen,<br>berücksichtigen Sie dies bitte.
Ich wünsche mir ein kostenloses Programm Videokonferenzprogramm,ähnlich wie Zoom , das für die Mitglieder meiner Selbsthilfegruppe, alles Rentner, ganz einfach zu bedienen ist.
Ich wünsche mir häufig eine klare und verbindliche Sprache, die rasch zur Sache kommt.
Ich würde es gut finden, wenn der Zugang zu Studien einfacher wäre und nicht, wie so oft, Medizinern etc vorbehalten wäre.
Ich würde gerne digitale Dienste wie die elektronische Patientenakte nutzen, habe bisher aber noch keine Ärzte gefunden, die sich beteiligen. Bisher ist das insofern recht eingeschränkt nutzbar.
Ich würde gerne nutzen E Rezept mache. Aber meine Ärzte nicht, Onlineshop Rezept einscannen(Daten und Ressourcen verbrauchen) letztendlich Rezept im Papierform hinbringen. Patientenakte konnte aufgrund Auseis dann PIN extra Anforderung dann persönlich erscheinenKrankenkasse noch nicht voll angelegt werden.
Ich würde gerne viel mehr digitale Medien und Anwendungen benutzen, z.B. e-Krankenakte, e-rezept, Videosprechstunde, aber mein Hausarzt bietet dies noch nicht an.
ich würde mir hier von Seiten der Selbsthilfeorganisationen intern PC Schulungen wünchen, derade bei Älteren.

In Deutschland läuft schon seit einigen Jahren in Digitalisierung sehr wenig. Ob im öffentlichen Dienst, in der Politik oder auch im Gesundheitswesen. Der Datenschutz wird immer vorgeschoben. Dabei nutzt fast jeder ein Smartphone und gibt für Apps und andere Dienstleistungen seine Daten preis. Und das sind oft die Menschen, die sich der Digitalisierung in den Weg stellen. Das ist meine Sichtweise.
in leichter Sprache um zu setzen
In meinem Umfeld kenne ich Menschen, die sich die viel zu teure Technik (Handy, Laptop, PC) gar nicht leisten können. Infolgedessen fehlt dann auch die Übung damit. Das halte ich auch für eine Art Ausgrenzung.
Ja, ich halte diese ganze digitalen Humbug für überflüssig. Du hast früher einen Arzt in fast jeder Gemeinde gehabt, welcher unter umständen auch zu erreichen ist. Dazu kam die Gemeindeschwester. Heute musste sonnstwo anrufen um einen Arzt zu bekommen , der letztlich vom benötigten Fachgebiet keine oder wenig Ahnung hat. Sinnvoller ist es, das Geldfür den digitalen Ausbau in den Ausbau des Hausarztnetzwerkes zu stecken, was letztlich allen zu gute kommt.Also den ersten Schritt machen und nich das Pferd von hinten aufzäumen !
Ja, mein Hausarzt hat nicht mal eine Emailadresse. Er benutzt immer noch ein Fax-Gerät. Eine Webseite hat er auch nicht.
je mehr ich digitale Medien nutze je mehr wird mir "passende" Werbung gezeigt, Wunderkerzen die Migräne heilen, spezielle Kissen oder Ernährung - manchmal ist es schwer diese Versprechen zu ignorieren eben weil man krank ist und Hoffnung sucht
Jeder erfindet das Rad neu. Viel unübersichtliche Werbung. Schnelles verklicken. Oftmals Störungen bei Zoom odr Teams...die nicht logisch sind und eine neue Einwahl erfordern. Fehlermeldungen nicht eindeutig, sondern es wird nur gesagt "Fehler" ...und dann guckt man dumm ;-)
Kein kuerzel
Kompatibilität wäre ein Thema, wenn z.b. die Herrsteller von […] mit zugehöriger Software sich zusammen schließen können und eine App für ein Standad entscheiden. Jeder macht sein Ding, es gibt keine wirkliche Norm... hier sollte nachgebessert werden.
Konkrete Weiterbildungsangebote bezüglich konkreter praktischer Nutzung, z.B. Erklärung von Apps, Umgang mit dem IPhone etc. wären sehr wünschenswert.
Kosten für Nutzung der Dig. Medien zu hoch __> (vorraussetzung Dig. Medien überhaubt zu nutzen)
Kostenfaktor
Kritich wenn persönliche Daten abgefragt werden
Leider gibt es immer noch viel zu wenige digitale Angebote.<br><br>Papier ist oft unnötig
Lesen auf Monitor oder Handy ist anstrengender als Printversionen
Lonks sind teilweise nicht beschriftet, auch andere bebilderte Titel.
Man muss sich erst damit anfreunden das vieles nun digital funktioniert früher musste man Überweisungen oder Kontoauszügen bei der Bank Vorort machen heute geht auch das alles digital per App, so ist es auch mit den Gesundheitsangelegenheiten wobei ich finde Gesundheit ist nochmal etwas persönlicher und spezifischer bei jedem Menschen mir fällt es persönlich schwerer mich da auf digitale Medien einzulassen

Manche Apps dind sindfremd aufgebaut und da man es keinen Spaß, daran zu arbeiten
Manche Webseiten werden nicht aktuell gehalten, manche Infos sind überholt. Wer sich nicht mit Internetrecherche auskennt ist sicher oftmals überfordert. Insbesondere objektive und fachliche Infos von dem Gegenteil zu erkennen.
Manche websiten sind immer noch nicht smartphone-gerecht aufgebaut, was die Suche und das Lesen erschwert. Konzentration ist bei den meisten Patientinnen eines der Hauptprobleme um im Netz die richtigen Infprmationen zu filtern bzw.zu erkennen.
mangelhaftes angebot seitens der patientenorganisation insbesondere was foren anbelangt
Mehrfache Authentifizierung und Registrierung sowie kompliziertes Einloggen mit Kundennummern etc. macht alles umständlich.
Meine Ärzte und Kliniken bieten keine Videosprechstunden, eRezept und EPA-Anschluß an
Meine Gruppe besteht aus älteren TeilnehmerInnen. Die IT-Kenntnisse sind kaum vorhanden. Es gibt eine Ablehnung von Video-Konferenzen oder Emails werden nicht angesehen.
meine persönliche Meinung: Gesundheitsdaten gehören nicht aufs Handy in eine App (App für Blutzuckermessung, digitale Patientenakte,...) oder in eine Cloud aus Datenschutzgründen.
Meine Zweifel an dem Datenschutz haben mich erst vor kurzem zur Nutzung von Apple geführt. Jetzt baue ich die Nutzung digitaler Angebote aus.
Meiner Meinung nach gibt es zu wenige digitale Angebote und Möglichkeiten.
Mir fehlt der persönliche Kontakt und das direkte Ringehen auf individuelle Fragen.
Mir fehlt die Sicherheit im Umgang. Ich bräuchte jemand, mit dem ich den Umgang üben könnte. Spannend finde ich das Ganze schon und würde es auch gerne beherrschen.
Mir fehlt oft der Speicherplatz, Übersicht über all die Apps, viele sind anders aufgebaut, immer ein neues Passwort
Mir gefällt ein persönliches Gespräch immer noch am Besten. Mit 74 Jahren und einer […] strengt lesen sehr an.
Mir ist der persönliche Kontakt lieber, weil Fragen besser geklärt werden können
Mir ist Papier in der Hand und Erklärungen von Personen, die ich persönlich vor mir sehe - einfach lieber. Bei digital habe ich immer den Eindruck "keine Zeit haben zu dürfen": Was mich im Internet wirklich stört, sind die diversen Verlinkungen - denn dadurch erfolgt ein Überfrachten der Informationen und am Schluß, übersehe ich etwas, weil ich zu meinem ursprünglichen Thema bzw. die Antwort / Erklärung dazu, nicht mehr zurückfinde.
Mit dem Handy habe ich Probleme, da ich sehr schwerhörig bin!
möglichst keine sensiblen Daten online
Nein
Neulingen bei Videokonferenzen sollte ein kurzes Schmnuppertrainig angeboten werden
Nicht nur auf digitale Angebote setzen- in meiner Generation oder älteren sind viele, denen einfach die Übung mit den digitalen Medien fehlt
Nutzungen die nur auf dem Smartphone funktionieren und nicht mehr am PC lehne ich ab und nutze sie nicht. Z.B. Krankenkassenangelegenheiten
Oft bleibt die Unsicherheit, ob digitale Informationen überhaupt vertrauenswürdig sind.

Oft werden passende Schlagzeilen als Blickfang in Suchmaschinen genutzt und die Information auf der Seite weicht dann ab
Öfter lesbarer/größer darstellen!
Online Gesprächskreise bieten eine gute Möglichkeit der Teilnahme, da ich in meiner Mobilität eingeschränkt bin.
Probleme mit Fachausdrücken im Computerbereich
Red erspart Weg, und Wartezeiten
schön wären ein/wenige landesweite Standards auf die sich alle Praktizierenden einigen können
Sie sollten transparent, einfach zu bedienen sein, eine übersichtliche Auswahl der Optionen vorgeben, damit man nicht lange suchen muss; in einer leicht verständlichen Sprache aufgebaut sein.
Sie wären eine große Hilfe und würden viele Wege sparen. Es ließen sich der Alltag und das Arbeitsleben besser organisieren.
Solange mir die digitalen Medien helfen gebe ich gerne meine Daten zur Verfügung.
Speicherung in Drittländer, bestimmten Diensten wird von mir eher als schwierig bewertet, da erhebliche Zweifel an der Datensicherheit besteht. Die veränderte Weltsituation (viele radikale Kräfte und zahlungsstarke Personen), deren Interessen nicht dem Gemeinwohl der Bevölkerung dienen sondern eigenen Interessen.
Standardisierung und Strukturierung finde ich besonders wichtig. Patienten sollten nicht für jedes Videoformat oder jede Information eine andere Applikation brauchen müssen (Zoom, Webex, Teams, Jitsi etc.). Auch die Notwendigkeit der Registrierung auf vielen unterschiedlichen Plattformen ist nervig. Datenschutzerklärungen müssen so ausführlich wie nötig aber so kurz wie möglich gestaltet werden.
Ständig auf dem neuesten technischen Stand bleiben zu müssen, finde ich sehr anstrengend und zeitraubend. Dass es viele Updates nach kurzer Zeit nicht mehr gibt, ist ärgerlich.
Teilweise fehlt grundlegend die Möglichkeit für die digitale Nutzung.
Teilweise zuviele Angebote. Überall soll man sich anmelden und bekommt dann E-Mails.<br>Man kann schwer unterscheiden was seriös und hilfreich ist und was letztendlich nur Abzocke ist und den E-Mail account zumüllt
Verbindung der Selbsthilfeorganisationen mit Online- Selbsthilfegruppen z.B. in Facebok
Verlust der Daten (Doku Medikamenteneinnahme) oder Gesundheitsdate/diagnosen fallen in falsche Hände ist ein Thema was mich hemmt digitale Medien zu nutzen
Viel zu speziell versteckt die Stellen wo wann wie man sich informiert macht
Viele ältere SHG Mitglieder haben keinen der ihnen die Computer "einrichtet" , damit es für sie leichter ist sie zu benutzten.
Viele der angebotenenen Sachen funktionieren doch gar nicht richtig, wie Z.B. elektronische Gesundheitskarte, eImpfausweis,<br>Dazu kommt noch, das die Bundesländer zum Teil ihr eigenes Süppchen kochen.<br>Es gibt doch gar keine Notwendigkeit den Kunden zu helfen oder das Leben zu erleichtern, oder es dauert Jahre bis es umgesetzt wird.<br>Deutschland ist da meilenweit von anderen Ländern entfernt.

Viele Menschen haben keinen PC oder kennen sich nicht mit Onlineaustausch aus, sodass wärend der Pandemie keine Selbsthilfetreffen stattfinden konten. Hierfür fehlte Unterstützung durch die Krankenkassen oder Paritätische Organisationen.
Vielfach fühle ich mich von der Vielzahl überschüttet
Vor allem hätte ich datenschutzbezogene bedenken, im Bereich von IT-Security. Besonders wichtig ist der Schutz meiner Privatsphäre!
Vor-&Nachteile genauer Auflisten, genauso mit Medikamenten Verfahren. Herzlichen Dank
Weil es noch keine Angebote gibt
wenn alles nur auf digitaler Medien -Welt aufgebaut wird, dann bleiben die persönlichen Kontakte auf der Strecke, dass wäre sehr schade.
Wenn der Internetzugang nicht schnell genug (Übertragungsrate nicht optimal) ist, dann kann es zu Schwierigkeiten beim Verstehen kommen, da das Hören mit Cochlea-Implantaten absolut gute Übertragung von Sprache benötigt.
Wenn die Informationen zu kompliziert dargestellt werden und oft geändert werden.
Wenn die Schrift zu klein ist
Wenn die Verifizierung zu kompliziert wird
Wenn ich immer schwere Passwörter brauche.
Wenn vieles auf Englisch beschrieben wird, ist das für mich nicht nutzbar
Wichtig ist der Datenschutz und die Transpatdes Hostings, da es sich ggf um besonders schützenswerte Daten handelt
Wichtig ist zu wissen, wo meine persönlichen Daten abgelegt sind und wer offiziell darauf zugreifen kann.
Wichtige Punkte sind für mich auch Transparenz; was passiert mit meinen Daten; Kontrolle über meine Daten, so dass ich entscheiden kann, wer was sehen darf und wer was bekommt
Wie kann ich an den Erfahrungen anderer Selbsthilfegruppen teilhaben?
Wollte ePA nutzen, musste meine E-Mail-Adresse zwischendurch ändern und der ganze Account wurde wieder gelöscht. Jetze alles wieder einzurichten, scheue ich mich davor.
Würde mir mehrere leicht zu findende und zu benutzende Foren für Patienten wünschen - evtl auch die Möglichkeit, niederschwellig Experten zu fragen - auch über sekundäre oder Folgekrankhriten!
Zugang zu digitalen Angeboten sollte leicht, unbürokratisch und unkompliziert sein<br><br>digitale Angebote wie die ePatientenakte bringen nichts, wenn sie zu kompliziert aufgebaut sind und wenn Arztpraxen/Apotheken, etc sie nicht nutzen können oder nicht nutzen und wenn Informationen nur von den Ärzten/Apotheken, etc. eingetragen werden können<br><br>ePatientenakte nur am Handy finde ich nicht gut
Zur Informationsbeschaffung und zum Austausch mit gleichgesinnten, insbesondere bei selteneren erkrankungen ist das Internet ein Segen, da man diese Menschen sonst nicht begegnet und auch sonst komplett von der Kompetenz und Informatonswillens des Arztes abhängig wäre.
