# Supplementary material for: Social factors associated with the development of e-health literacy: A mixed-methods study among patient organization members and representatives
Source: PLOS Digit Health. 2026 Aug 13;5(8):e0001569. doi: 10.1371/journal.pdig.0001569 (PMC13472428; doi:10.1371/journal.pdig.0001569)
Supplement: Supplementary file 5 [file pdig.0001569.s005.pdf]

Online Survey (German original)

PANDORA: Befragung der Mitglieder von Selbsthilfe- und Patientenorganisationen

Stand: 08.08.2023

Rubrik ND: Nutzung digitaler Medien und Angebote

Das Gesundheitswesen befindet sich im digitalen Wandel. Digitale Medien und Angebote eröffnen neue und immer vielfältigere Möglichkeiten u. a., um sich zu informieren, Gesundheitsleistungen wahrzunehmen, sich zu vernetzen oder die eigenen Gesundheitsdaten zu speichern. Daher interessiert uns zunächst ganz allgemein, ob bzw. welche digitale Medien und Angebote Sie für Ihre gesundheitsbezogenen Anliegen und Fragen nutzen. Einige digitale Möglichkeiten haben wir im Folgenden aufgelistet.

[ND02] Mehrfachauswahl

"Welche digitalen Medien und/oder Angebote nutzen Sie in Ihrem Alltag für Ihre gesundheitsbezogenen Anliegen und Fragen?"

ND02_01 Internet (z. B. Websites, Suchmaschinen)
ND02_02 E-Mails
ND02_03 Messengerdienste (z. B. WhatsApp oder Signal)
ND02_04 Dienste für Videokonferenzen (z. B. Zoom oder Skype)
ND02_05 soziale Medien (z. B. Facebook, Twitter, Instagram, TikTok)
ND02_06 Video- und/oder Musik-Streamingdienste (z. B. YouTube, Netflix, Spotify)
ND02_07 Podcast-Streamingdienste (z. B. Spotify, Apple Podcasts)
ND02_08 Videosprechstunden
ND02_09 Telemonitoring (z. B. per App oder tragbarem Messgerät)
ND02_10 Online-Apotheken
ND02_11 Gesundheits-/Fitness-Apps (z. B. DiGAs)
ND02_12 elektronische Patientenakte (ePA)
ND02_13 elektronischer Medikationsplan (eMP)
ND02_14 elektronischer Impfpass (E-Impfpass)
ND02_15 elektronischer Mutterpass (E-Mutterpass)
ND02_16 elektronisches Rezept (eRezept)
ND02_17 Sonstige digitale Medien/Angebote

1 = nicht gewählt
2 = ausgewählt

ND02_17a Sonstige digitale Medien/Angebote

Offene Texteingabe

Ausweichoption

-1 = Ich nutze keine digitalen Medien/Angebote für meine gesundheitsbezogenen Anliegen/Fragen.

[ND03] Mehrfachauswahl | **Filterfrage: ND03 wird nur angezeigt, wenn unter ND02 ausgewählt wurde, dass ‚keine digitalen Medien/Angebote' genutzt werden.**

"Warum nutzen Sie keine digitalen Medien/Angebote für Ihre gesundheitsbezogenen Anliegen/Fragen?"

ND03_01 ...nicht weiß, welche digitalen Angebote/Medien für mich geeignet sind.
ND03_02 ...Bedenken bezüglich der Sicherheit meiner Daten habe.
ND03_03 ...mich lieber persönlich mit Gesundheitsfachleuten austausche.
ND03_04 Sonstige Gründe

1 = nicht gewählt
2 = ausgewählt

ND03_04a Sonstige Gründe

Offene Texteingabe

[ND04] Auswahl

"Wie schätzen Sie generell Ihre persönlichen Fähigkeiten ein, digitale Medien und/oder Angebote zu bedienen und damit umzugehen?"

1 = sehr gut
2 = eher gut
3 = eher schlecht
4 = sehr schlecht
-1 = keine Angabe
-9 = nicht beantwortet

[ND05] Skala

"Uns interessiert im Folgenden, ob bestimmte Umstände die Nutzung von digitalen Medien und/oder Angeboten grundsätzlich für Sie erschweren."

Ich habe Schwierigkeiten bei der Nutzung von digitalen Medien/Angeboten, wenn...

ND05_01 ...sie für mich nicht barrierefrei sind (z. B. fehlende Optionen für Untertitel, Textvergrößerungen oder Sprachsteuerung).

ND05_02 ...sie kompliziert aufgebaut sind, weil ich dann nicht direkt finden kann, was ich suche.

ND05_03 ...sie sehr viele Informationen enthalten, weil ich mich dann schnell überfordert fühle.

ND05_04 ...ich nicht genau weiß, was mit meinen persönlichen Daten passiert.

ND05_05 ...Texte und Informationen in Fachsprache formuliert sind.

ND05_06 ...ich dafür einen schnellen Internetanschluss benötige.

ND05_07 ...ich dafür ein Smartphone brauche und mich gut mit dessen Funktionen auskennen muss.

ND05_08 ...ich mich dafür gut mit Computern auskennen muss.

1 = trifft gar nicht zu
2 = trifft eher nicht zu
3 = trifft eher zu
4 = trifft völlig zu
-1 = keine Angabe
-9 = nicht beantwortet

[ND06] Texteingabe offen

Sonstiges (bzgl. ND05)

ND06_01 Sonstige Schwierigkeiten, und zwar

Offene Texteingabe

[ND07] Texteingabe offen

"Gibt es noch etwas, das Sie uns zum Thema „Nutzung digitaler Medien und Anwendungen" mitteilen möchten?"

Offene Texteingabe

Rubrik MI: Fragen zu Ihrer Mitgliedschaft in Ihrer Organisation

Im Folgenden möchten wir nun etwas über Ihre Mitgliedschaft in Ihrer Patienten- bzw. Selbsthilfeorganisation erfahren.

[MI02] Auswahl

"Sind Sie Mitglied in einer Patienten- bzw. Selbsthilfeorganisation?"

1 = ja
2 = nein
-9 = nicht beantwortet

[MI03] Auswahl
"Was beschreibt den Hintergrund Ihrer Mitgliedschaft am ehesten?"

1 = Betroffene/r (erkrankt/behindert)
2 = Angehörige/r einer betroffenen Person
3 = Sonstiges, und zwar:
-1 = keine Angabe
-9 = nicht beantwortet

MI03_03 Sonstiges, und zwar
Offene Texteingabe

[MI04] Auswahl
"Wie lange sind Sie bereits Mitglied in Ihrer Organisation?"

1 = kürzer als 1 Jahr
2 = 1 bis 2 Jahre
3 = 3 bis 5 Jahre
4 = 6 bis 10 Jahre
5 = länger als 10 Jahre
-9 = nicht beantwortet

[MI05] Auswahl
"Engagieren Sie sich neben Ihrer Mitgliedschaft aktuell auch ehrenamtlich in Ihrer Organisation?"

1 = ja
2 = nein
-1 = keine Angabe
-9 = nicht beantwortet

[MI06] Mehrfachauswahl | **Filterfrage: MI06 wird nur angezeigt, wenn MI05 mit ‚ja' beantwortet wurde.**
"Bitte kreuzen Sie alle ehrenamtlichen Aufgaben an, die Sie derzeit in Ihrer Organisation übernehmen."

MI06_01 Leiter/in einer Selbsthilfegruppe bzw. Gruppensprecher/-in
MI06_02 Mitglied in regionalen Arbeitsgruppen, Gremien oder Foren
MI06_03 Mitglied in nationalen Arbeitsgruppen, Gremien oder Foren
MI06_04 Berater/in für andere Mitglieder / Betroffene
MI06_05 Forschungspartner/in (z. B. Mitarbeit an Studien)
MI06_06 Lotse/Lotsin
MI06_07 Berater/in
MI06_08 Sprecher/in
MI06_09 Schatzmeister/in
MI06_10 Andere Aufgaben, und zwar
1 = nicht gewählt
2 = ausgewählt

MI06_10a Andere Aufgaben, und zwar
Offene Texteingabe

Rubrik DO: Digitalisierung in Ihrer Patienten- bzw. Selbsthilfeorganisation

Im Folgenden möchten wir Ihnen einige Fragen zur Digitalisierung in Ihrer Organisation stellen.

[DO02] Skala
"Gibt es in Ihrer Organisation die folgenden digitalen Angebote?"

DO02_01 Internetauftritt / Website
DO02_02 Kommunikation über E-Mails
DO02_03 Digitaler Newsletter
DO02_04 Online-Beratung (z.B. über Zoom, Big Blue Button)
DO02_05 Online-Selbsthilfegruppen
DO02_06 Online-Schulung / Webinar über Internet (z.B. über Zoom, Big Blue Button)
DO02_07 Online-Diskussionsforum
DO02_08 Angebote in sozialen Medien (z.B. Facebook, Instagram, Twitter)
DO02_09 digitales Patientenregister
DO02_10 Austauschmöglichkeit über Messengerdienste (z. B. WhatsApp oder Signal)
DO02_11 Online-Umfragen oder -Abstimmungen (z. B. über Doodle)
DO02_12 eigene App
DO02_13 YouTube-Kanal
DO02_14 Video-Angebote (z. B. Videotutorials oder Aufzeichnungen von Veranstaltungen)
DO02_15 Podcast / Audio-Angebote

1 = ja
2 = nein
-1 = weiß nicht
-9 = nicht beantwortet

[DO03] Auswahl + Texteingabe offen
Sonstiges (bzgl. DO02)

1 = Sonstiges, und zwar:
+ Offene Texteingabe
-9 = nicht beantwortet

[DO04] Skala | **Filterfrage: Es werden nur die digitalen Angebote/Medien angezeigt, die zuvor unter DO02 ausgewählt wurden.**
"Wie häufig nutzen Sie diese digitalen Angebote Ihrer Organisation?"

DO04_01 Internetauftritt / Website
DO04_02 Kommunikation über E-Mails
DO04_03 Digitaler Newsletter
DO04_04 Online-Beratung (z.B. über Zoom, Big Blue Button)
DO04_05 Online-Selbsthilfegruppen
DO04_06 Online-Schulung / Webinar über Internet (z.B. über Zoom, Big Blue Button)
DO04_07 Online-Diskussionsforum
DO04_08 Angebote in sozialen Medien (z.B. Facebook, Instagram, Twitter)
DO04_09 digitales Patientenregister
DO04_10 Austauschmöglichkeit über Messengerdienste (z. B. WhatsApp oder Signal)
DO04_11 Online-Umfragen oder -Abstimmungen (z. B. über Doodle)
DO04_12 eigene App
DO04_13 YouTube-Kanal
DO04_15 Video-Angebote (z. B. Videotutorials oder Aufzeichnungen von Veranstaltungen)
DO04_14 Podcast / Hör-Angebote

1 = gar nicht
2 = seltener als einmal pro Monat
3 = einmal pro Monat
4 = etwa alle zwei Wochen
5 = einmal pro Woche
6 = mehrmals pro Woche

7 = täglich
-9 = nicht beantwortet

[DO05] Texteingabe offen
„Wie häufig nutzen Sie das digitale Angebot, das Sie unter Sonstiges angegeben haben?" (bzgl. DO04)

Offene Texteingabe

[DO06] Auswahl + Texteingabe offen
"Wie zufrieden sind Sie mit der Bandbreite der digitalen Angebote Ihrer Organisation?"

1 = Sehr zufrieden, weil:
+ Offene Texteingabe

2 = Eher zufrieden, weil:
+ Offene Texteingabe

3 = Eher unzufrieden, weil:
+ Offene Texteingabe

4 = Sehr unzufrieden, weil:
+ Offene Texteingabe

-1 = keine Angabe
-9 = nicht beantwortet

[DO07] Texteingabe offen
"Welche digitalen Angebote wünschen Sie sich (zusätzlich) von Ihrer Organisation?"

Offene Texteingabe

Rubrik PA: Beteiligung von Mitgliedern an der Planung und Gestaltung von digitalen Angeboten

Stellen Sie sich bitte folgende Situation vor:

Ihre Organisation möchte die Webseite umgestalten oder eine App für ihre Mitglieder entwickeln. Mit diesen neuen Angeboten könnten Sie zum Beispiel an Umfragen teilnehmen, aktuelle Informationen Ihrer Organisation lesen, sich über Erkrankungen bzw. Behinderungen informieren, eigene Daten einstellen und mit anderen Mitgliedern einfacher als zuvor kommunizieren. Um diese Anwendungen zu gestalten (z. B. welche Funktionen sie enthalten sollen, wie die Inhalte dargestellt werden sollen usw.), bittet Ihre Organisation Sie und andere Mitglieder, sich in den Entwicklungsprozess einzubringen.

Beantworten Sie zu dieser Situation bitte nun die folgenden Fragen.

[PA02] Auswahl
"Wären Sie grundsätzlich daran interessiert, sich an der Planung und Gestaltung eines digitalen Angebots Ihrer Organisation zu beteiligen?"

1 = ja
2 = nein
-1 = keine Angabe
-9 = nicht beantwortet

[PA03] Skala | **Filterfrage: PA03, PA04 und PA05 werden nur angezeigt, wenn Frage PA02 mit ‚ja' beantwortet wurde.**
"Im Folgenden geht es um verschiedene Möglichkeiten der Beteiligung an der Planung und Gestaltung eines digitalen Angebots."

Ich könnte mir gut vorstellen, dass ich…

PA03_01 ...eine Veranstaltung meiner Organisation besuche, auf der die Mitglieder über die Entwicklung des digitalen Angebots informiert werden.

PA03_02 ...an einer Umfrage teilnehme, damit meine Bedürfnisse und Wünsche erfasst werden.

PA03_03 ...die verantwortlichen Personen berate (z. B. in Bezug auf Funktionen oder Inhalte, die ich sinnvoll oder wichtig finde).

PA03_04 ...eine erste Version (Prototyp) des digitalen Angebots ausprobiere und Feedback gebe.

PA03_05 ...einem Mitgliederbeirat beitrete, der sich regelmäßig mit den verantwortlichen Personen austauscht und ein Mitspracherecht hat.

PA03_06 ...gemeinsam mit den verantwortlichen Personen alle Entscheidungen im Entwicklungsprozess treffe.

1 = trifft gar nicht zu
2 = trifft eher nicht zu
3 = trifft eher zu
4 = trifft völlig zu
-1 = keine Angabe
-9 = nicht beantwortet

[PA04] Skala

" Im Folgenden geht es darum, wie die Rahmenbedingungen gestaltet sein müssten, damit Sie sich in die Planung und Gestaltung eines digitalen Angebots Ihrer Organisation einbringen würden.“

Ich würde mich an solch einem Entwicklungsprojekt beteiligen,…

PA04_01 ...wenn ich mich kompetent genug fühle, um bei den anstehenden Aufgaben unterstützen zu können.

PA04_02 ...wenn die Zusammenarbeit zwischen allen Beteiligten gleichberechtigt und respektvoll abläuft.

PA04_03 ...auch wenn meine Rolle oder meine Aufgaben im Entwicklungsprozess nicht klar definiert sind.

PA04_04 ...auch wenn ich den Prozess nicht wirklich beeinflussen kann (z. B. durch ein Mitbestimmungsrecht).

PA04_05 ...wenn ich keine Verantwortung übernehmen muss.

PA04_06 ...wenn es sich um ein Projekt handelt, das ich als relevant bzw. nützlich für mich und die anderen Mitglieder einschätze.

PA04_07 ...wenn der zeitliche Aufwand so gering wie möglich ist.

PA04_08 ...wenn ich angemessen darauf vorbereitet werde (z. B. durch eine Schulung).

PA04_09 ...auch wenn ich dafür keine finanzielle Kompensation erhalte.

1 = trifft gar nicht zu
2 = trifft eher nicht zu
3 = trifft eher zu
4 = trifft völlig zu
-1 = keine Angabe
-9 = nicht beantwortet

[PA05] Texteingabe offen

Sonstiges (bzgl. PA04)

Offene Texteingabe

[PA06] Mehrfachauswahl | **Filterfrage: PA06 wird nur angeteigt, wenn PA02 mit ‚nein‘ beantwortet wurde.**

"Warum wären Sie nicht daran interessiert, sich aktiv an der Planung und Gestaltung digitaler Angebote in Ihrer Organisation zu beteiligen?"

Ich wäre nicht daran interessiert, weil ich...

PA06_01 ...dafür keine Zeit habe.

PA06_02 ...keinen Mehrwert für mich in einer Beteiligung sehe.

PA06_03 ...unsicher bin, ob meine (technischen) Kenntnisse dafür ausreichen.

PA06_04 ...negative Erfahrungen mit einem ähnlichen Projekt gemacht habe.

PA06_05 ...mich grundsätzlich nicht für digitale Angebote interessiere.

PA06_06 Sonstige Gründe

1 = nicht gewählt
2 = ausgewählt

PA06_06a Sonstige Gründe

Offene Texteingabe

[PA07] Auswahl

"Hat Ihre Organisation Sie schon einmal gefragt, ob Sie an der Planung und Gestaltung digitaler Angebote mitwirken würden?"

1 = ja
2 = nein
-1 = keine Angabe
-9 = nicht beantwortet

[PA08] Auswahl | **Filterfrage: PA08 wird nur angezeigt, wenn PA07 mit ‚ja' beantwortet wurde.**

"Haben Sie dieser Anfrage Ihrer Organisation zugestimmt?"

1 = ja
2 = nein
-1 = keine Angabe
-9 = nicht beantwortet

[PA09] Mehrfachauswahl | **Filterfrage: PA09, PA10 und PA11 werden nur angezeigt, wenn PA08 mit ‚ja' beantwortet wurde.**

"Welches digitale Angebot wurde dabei entwickelt?"

PA09_01 Internetauftritt / Website

PA09_02 App

PA09_03 digitales Patientenregister

PA09_04 digitaler Newsletter

PA09_05 Sonstiges, und zwar

1 = nicht gewählt
2 = ausgewählt

PA09_05a Sonstiges, und zwar

Offene Texteingabe

[PA10] Mehrfachauswahl

"An welchen der folgenden Aktivitäten waren Sie dabei beteiligt?"

PA10 Aktivitäten im Entwicklungsprozess: Ausweichoption (negativ) oder Anzahl ausgewählter Optionen

Ganze Zahl

PA10_01 Äußerung von Wünschen per Umfrage, Interview, Gruppendiskussion o. Ä.

PA10_02 Konkrete Planung des digitalen Angebots (z. B. in einer Arbeitsgruppe).

PA10_03 Entwicklung von Inhalten (z. B. Texte, Videos o. Ä.) für das digitale Angebot.

PA10_04 Gestaltung eines Prototyps.

PA10_05 Ausprobieren und Bewertung des Prototyps.

PA10_06 Weiterempfehlung des digitalen Angebots.

PA10_07 Schulung anderer Mitglieder im Umgang mit dem digitalen Angebot.

PA10_08 Sonstige Aktivitäten, und zwar

1 = nicht gewählt
2 = ausgewählt

PA10_08a Sonstige Aktivitäten, und zwar

Offene Texteingabe

[PA11] Auswahl + Texteingabe offen
"Wie würden Sie Ihre Erfahrungen beim Mitwirken an den genannten Aktivitäten insgesamt bewerten?"

1 = Sehr positiv, weil:
+ Offene Texteingabe

2 = Eher positiv, weil:
+ Offene Texteingabe

3 = Eher negativ, weil:
+ Offene Texteingabe

4 = Sehr negativ, weil:
+ Offene Texteingabe

-1 = keine Angabe
-9 = nicht beantwortet

[PA12] Mehrfachauswahl | **Filterfrage: PA12 werden nur angezeigt, wenn PA08 mit ‚nein' beantwortet wurde.**
"Warum haben Sie diese Anfrage Ihrer Organisation abgelehnt?"

Ich habe diese Anfrage abgelehnt, weil ich...
PA12_01 ...keine Zeit dafür hatte.
PA12_02 ...in dem digitalen Angebot keinen Mehrwert für mich gesehen habe.
PA12_03 ...unsicher war, ob meine (technischen) Kenntnisse dafür ausreichen.
PA12_04 ...nicht ausreichend informiert war.
PA12_05 ...negative Erfahrung mit einem ähnlichen Projekt gemacht habe.
PA12_06 Sonstige Gründe
1 = nicht gewählt
2 = ausgewählt
PA12_06a Sonstige Gründe
Offene Texteingabe

[PA13] Texteingabe offen
"Möchten Sie uns noch etwas zum Thema „Beteiligung von Mitgliedern an der Planung und Gestaltung von digitalen Angeboten" mitteilen?"

Offene Texteingabe

Rubrik KO: Zusammenarbeit mit Kooperationspartnern

Stellen Sie sich bitte folgende Situation vor:

Gemeinsam mit einigen technisch bewanderten Mitgliedern hat Ihre Organisation ein digitales Angebot entwickelt. Damit können Mitglieder u. a. Krankheitssymptome und Nebenwirkungen von Medikamenten eigenständig dokumentieren. Dabei haben sie die alleinige Kontrolle über ihre Daten. Viele Mitglieder nutzen dieses Angebot und finden es hilfreich. Das digitale Angebot ist aber noch nicht vollständig ausgereift. Der Vorstand schlägt daher vor, es professionell weiterzuentwickeln. Ihre Organisation möchte dabei mit einem Kooperationspartner zusammenarbeiten, weil notwendige Mittel fehlen (z. B. finanzielle Mittel oder technische Kenntnisse). Das digitale Angebot soll im Anschluss auch Nutzern und Nutzerinnen außerhalb Ihrer Organisation zur Verfügung gestellt werden.

Beantworten Sie zu dieser Situation bitte nun die folgenden Fragen.

[KO02] Skala

"Für die (Weiter-)Entwicklung eines digitalen Angebots können verschiedene Kooperationspartner infrage kommen. Wie würden Sie eine Zusammenarbeit Ihrer Organisation mit den Folgenden bewerten?"

Zusammenarbeit mit...

KO02_01 ...einer anderen Patienten- / Selbsthilfeorganisation.
KO02_02 ...einem Technologie-Unternehmen / Softwareentwickler.
KO02_03 ...einer Behörde (z. B. Gesundheitsamt).
KO02_04 ...einer Forschungseinrichtung (z. B. Universität).
KO02_05 ...einer religiösen Einrichtung.
KO02_06 ...einem Krankenhauskonzern / Klinikverbund.
KO02_07 ...einer Krankenkasse.
KO02_08 ...einem Medizintechnikhersteller.
KO02_09 ...einem Pharmaunternehmen.

1 = sehr negativ
2 = eher negativ
3 = eher positiv
4 = sehr positiv
-1 = keine Angabe
-9 = nicht beantwortet

[KO03] Skala

"Was könnte dazu führen, dass Sie eine Zusammenarbeit zwischen Ihrer Organisation und einem Kooperationspartner ablehnen?"

Ich würde eine Zusammenarbeit ablehnen, wenn…

KO03_01 ...ich die Absichten und Interessen des Kooperationspartners nicht transparent nachvollziehen kann.
KO03_02 ...ich Negatives über den Kooperationspartner gehört habe (z. B aus den Medien oder aus meinem sozialen Umfeld).
KO03_03 ...der Kooperationspartner nicht meine persönlichen Werte vertritt.
KO03_04 ...meine Organisation keine finanziellen Leistungen von dem Kooperationspartner erhält.
KO03_05 ...meine Organisation nicht die Hauptverantwortung für den (Weiter-)Entwicklungsprozess des digitalen Angebots trägt.
KO03_06 ...es für mich oder andere Mitglieder keine Möglichkeit gibt, sich an dem (Weiter-)Entwicklungsprozess zu beteiligen.
KO03_07 ...dadurch der Kooperationspartner die Rechte an dem digitalen Angebot erhält (z. B. Eigentums- und Nutzungsrechte).

1 = trifft gar nicht zu
2 = trifft eher nicht zu
3 = trifft eher zu
4 = trifft völlig zu
-1 = keine Angabe
-9 = nicht beantwortet

[KO04] Texteingabe offen

Sonstiges (bzgl. KO03)

Offene Texteingabe

[KO05] Skala

"Wie würden Sie grundsätzlich eine Weiterentwicklung von digitalen Angeboten Ihrer Organisation bewerten?"

Ich würde es gut finden, wenn meine Organisation…

KO05_01 ...ihre digitalen Angebote weiterentwickelt, um z. B. neue Funktionen hinzuzufügen.

KO05_02 ...Kooperationspartner in solche Prozesse einbezieht.

KO05_03 ...die weiterentwickelten Angebote ausschließlich den eigenen Mitgliedern zugänglich macht.

1 = trifft gar nicht zu
2 = trifft eher nicht zu
3 = trifft eher zu
4 = trifft völlig zu
-1 = keine Angabe
-9 = nicht beantwortet

[KO06] Texteingabe offen

"Möchten Sie uns noch etwas zum Thema „Zusammenarbeit mit Kooperationspartnern" mitteilen?"

Offene Texteingabe

Rubrik DD: Digitale Datenspende

Stellen Sie sich bitte folgende Situation vor:

Um Forschung zu ihrem Themengebiet (Erkrankung/Behinderung) proaktiv voranzutreiben, möchte Ihre Organisation die Gesundheitsdaten aller Mitglieder digital sammeln (z. B. in einem eigenen digitalen Patientenregister). Bei diesen Daten kann es sich z. B. um Informationen über den Krankheitsverlauf sowie die medizinische oder pflegerische Versorgung handeln. Die Daten sollen anonymisiert von Ihrer Organisation gespeichert und anschließend der Forschung zugänglich gemacht werden. Ihre Organisation bittet Sie, sich an dieser freiwilligen „Datenspende" zu beteiligen, damit auch Ihre Daten für Forschungsvorhaben verfügbar sind.

Beantworten Sie zu dieser Situation bitte nun die folgenden Fragen.

[DD02] Skala

"Inwiefern wären Sie bereit, Ihre Gesundheitsdaten Ihrer Organisation digital zur Verfügung zu stellen?"

Ich wäre dazu bereit,…

DD02_01 ...weil sich dadurch die Behandlungs- bzw. Versorgungsmöglichkeiten meiner Erkrankung bzw. Behinderung verbessern können.

DD02_02 ...weil ich dadurch dazu beitragen kann, die Behandlung bzw. Versorgung anderer Betroffener zu verbessern.

DD02_03 ...weil die anderen Mitglieder und die verantwortlichen Personen meiner Organisation das von mir erwarten.

DD02_04 ...auch wenn ich nicht genau weiß, wie meine Daten geschützt sind.

DD02_05 ...auch wenn ich meine Daten dafür per Messengerdienst (z. B. WhatsApp) verschicken muss.

DD02_06 ...wenn ich dafür eine finanzielle Gegenleistung erhalte.

DD02_07 ...wenn auch Forschungsideen von Mitgliedern der Organisation berücksichtigt werden.

DD02_08 ...auch wenn meine Daten von kommerziellen Akteuren (z. B. Pharmaunternehmen) für Forschungszwecke genutzt werden können.

DD02_09 ...wenn ich jedes Mal entscheiden kann, für welche Forschungsprojekte meine Daten verwendet werden.

DD02_10 ...um dadurch den Einfluss meiner Organisation auf die Forschung zu stärken.

1 = trifft gar nicht zu
2 = trifft eher nicht zu
3 = trifft eher zu
4 = trifft völlig zu

-1 = keine Angabe
-9 = nicht beantwortet

[DD03] Texteingabe offen
"Möchten Sie uns noch etwas zum Thema „Digitale Datenspende“ mitteilen?"

Offene Texteingabe

Rubrik ZE: Zusammenfassende Einschätzungen zur Bedeutung der Digitalisierung in Patienten- bzw. Selbsthilfeorganisationen

Digitalisierung in Patienten- und Selbsthilfeorganisationen findet auf unterschiedlichen Ebenen statt und Sie haben uns schon viele Hinweise in Bezug auf Ihre Einschätzung zur *Umsetzung*, *Beteiligung* und *Kooperation* gegeben.

[ZE02] Skala
"Abschließend möchten wir Sie daher fragen, wie Sie die folgenden Aspekte der Digitalisierung in Ihrer Organisation und deren zukünftige Weiterentwicklung beurteilen."

Wie wichtig finden Sie es, dass Ihre Organisation…

ZE02_01 ...sich digital weiterentwickelt und ihre digitalen Angebote ausweitet?
ZE02_02 ...die Mitglieder in die Gestaltung neuer digitaler Angebote einbezieht?
ZE02_03 ...sicherstellt, dass alle Mitglieder, unabhängig von ihren Fähigkeiten, die digitalen Angebote nutzen können?
ZE02_04 ...die Mitglieder dabei unterstützt, digitale Angebote nutzen zu können (z. B. durch eine Schulung/Beratung)?
ZE02_05 ...neben den digitalen Angeboten auch „analoge“ Alternativen (z. B. in Papierform oder persönlich) beibehält?
ZE02_06 ...nicht aus den Augen verliert, dass persönliche Kontakte für die emotionale Verbindung zwischen den Mitgliedern wichtig sind?
ZE02_07 ...Forschungsvorhaben vorantreibt, indem sie digitale Gesundheitsdaten der Mitglieder sammelt (z. B. per Patientenregister)?
ZE02_08 ...sich für mehr Digitalisierung im Gesundheitswesen einsetzt (z. B. durch politische Interessenvertretung)?
ZE02_09 ...sich aktiv an der Entwicklung von neuen digitalen Angeboten im Gesundheitswesen beteiligt (z. B. als Kooperationspartner)?

1 = gar nicht wichtig
2 = eher unwichtig
3 = eher wichtig
4 = sehr wichtig
-1 = keine Angabe
-9 = nicht beantwortet

[ZE03] Texteingabe offen
"Haben Sie zum Abschluss noch weitere Anmerkungen oder Kommentare zur Digitalisierung in Ihrer Patienten- bzw. Selbsthilfeorganisation?"

Offene Texteingabe

Rubrik SD: Angaben zu Ihrer Person

Bitte geben Sie uns zum Abschluss einige Informationen zu Ihrer Person.

[SD02] Auswahl
"Was ist Ihr Geschlecht?"

SD02 Geschlecht

1 = weiblich
2 = männlich
3 = divers
-1 = Möchte ich nicht angeben.
-9 = nicht beantwortet

[SD03] Auswahl + Texteingabe offen
"Wie alt sind Sie?"

SD03 Alter

1 = ich bin
+ Offene Texteingabe

-1 = Möchte ich nicht angeben.
-9 = nicht beantwortet

[SD04] Auswahl
"Was ist Ihr höchster allgemeiner Schulabschluss?"

SD04 Schulabschluss

1 = Fachhochschulabschluss / Hochschulabschluss
2 = Abitur bzw. Erweiterte Oberschule mit Abschluss der 12. Klasse
3 = Fachhochschulreife (Abschluss einer Fachoberschule etc.)
4 = Mittlere Reife, Realschulabschluss bzw. Polytechnische Oberschule mit Abschluss 10. Klasse
5 = Volks-/Hauptschulabschluss bzw. Polytechnische Oberschule mit Abschluss 8. oder 9. Klasse
6 = Von der Schule abgegangen ohne Schulabschluss
7 = (noch) ohne Schulabschluss
8 = anderer Abschluss:
+ Offene Texteingabe

-1 = Möchte ich nicht angeben.
-9 = nicht beantwortet

[SD05] Mehrfachauswahl + Texteingabe offen | **Filterfrage: SD05 wird nur angezeigt, wenn MI03 mit 1 (= Betroffene/r (erkrankt/behindert)) beantwortet wurde.**
"Welche (chronische) Erkrankung/en und/oder Behinderung haben Sie?"

SD05_01 Erkrankung des Bewegungsapparats
SD05_02 Psychische Erkrankung
SD05_03 Neurologische Erkrankung
SD05_04 Herz-Kreislauf-Erkrankung
SD05_05 Lungenerkrankung
SD05_06 Erkrankung des Verdauungsapparats
SD05_07 Diabetes mellitus Typ 2
SD05_08 Rheumatische Erkrankung
SD05_09 Autoimmunerkrankung
SD05_10 Krebs
SD05_11 Andere Erkrankung
SD05_12 Verlust oder Teilverlust von Gliedmaßen
SD05_13 Funktionseinschränkung von Gliedmaßen
SD05_14 Funktionseinschränkung der Wirbelsäule und des Rumpfes, Deformierung des Brustkorbes
SD05_15 Blindheit oder Sehbehinderung
SD05_16 Sprach- oder Sprechstörungen, Taubheit, Schwerhörigkeit, Gleichgewichtsstörungen
SD05_17 Verlust einer Brust oder beider Brüste, Entstellungen u.a.

SD05_18 Beeinträchtigung der Funktion von inneren Organen bzw. Organsystemen

SD05_19 Querschnittlähmung, Zerebrale Störungen, Geistig-Seelische Behinderungen, Suchtkrankheiten

SD05_20 Andere Behinderung

1 = nicht gewählt
2 = ausgewählt

SD05_11a Andere Erkrankung

SD05_20a Andere Behinderung

Offene Texteingabe

Ausweichoptionen

-1 = Ich habe weder eine (chronische) Erkrankung noch eine Behinderung.
-2 = Möchte ich nicht angeben.

[SD06] Auswahl + Texteingabe offen | **Filterfrage: SD05 wird nur angezeigt, wenn MI03 mit 2 (= Angehörige/r einer betroffenen Person) beantwortet wurde.**

"Sie haben zuvor angegeben, dass Sie Angehörige/r einer erkrankten/behinderten Person sind. Daher möchten wir nun gerne wissen, in welcher Beziehung Sie zu der betroffenen Person stehen."

Ich bin:

1 = Mutter / Vater
2 = Schwester / Bruder
3 = Ehepartner/-in / Lebenspartner/-in
4 = Tochter / Sohn
5 = Großmutter / Großvater
6 = Enkeltochter / Enkelsohn
7 = Tante / Onkel
8 = Cousine / Cousin
9 = Schwägerin / Schwager
10 = Nichte / Neffe
11 = Schwiegermutter / Schwiegervater
12 = Stiefmutter / Stiefvater
13 = Stiefschwester / Stiefbruder
14 = Sonstiges:

+ Offene Texteingabe

-1 = Möchte ich nicht angeben.
-9 = nicht beantwortet

[SD07] Auswahl + Texteingabe offen

"Zum Abschluss haben Sie hier die Möglichkeit anzugeben, in welcher Organisation Sie Mitglied sind."

1 = Ich bin Mitglied in der/dem:

+ Offene Texteingabe

-1 = Möchte ich nicht angeben.
-9 = nicht beantwortet
