# Supplementary material for: Social factors associated with the development of e-health literacy: A mixed-methods study among patient organization members and representatives
Source: PLOS Digit Health. 2026 Aug 13;5(8):e0001569. doi: 10.1371/journal.pdig.0001569 (PMC13472428; doi:10.1371/journal.pdig.0001569)
Supplement: Supplementary file 6 [file pdig.0001569.s006.pdf]

Table 1. Distribution of self-rated digital skills across participant characteristics.

	Self-rated digital skills				
	**Very good, n (%)**	**Rather good, n (%)**	**Rather poor, n (%)**	**Very poor, n (%)**	**p-Value**
**Age (n = 1,242)**					**<0.001[a]***
18–29 years (n = 44)	28 (63.6)	16 (36.4)	0 (0.0)	0 (0.0)	
30–39 years (n = 136)	75 (55.1)	55 (40.4)	6 (4.4)	0 (0.0)	
40–49 years (n = 197)	88 (44.7)	103 (52.3)	5 (2.5)	1 (0.5)	
50–64 years (n = 551)	166 (30.1)	326 (59.2)	53 (9.6)	6 (1.1)	
≥ 65 years (n = 314)	61 (19.4)	189 (60.2)	58 (18.5)	6 (1.9)	
**Gender (n = 1,294)**					**0.001[b]***
Female (n = 887)	273 (30.8)	506 (57.0)	98 (11.0)	10 (1.1)	
Male (n = 405)	168 (41.5)	203 (50.1)	30 (7.4)	4 (1.0)	
Non-binary or gender diverse (n = 2)	0 (0.0)	1 (50.0)	1 (50.0)	0 (0.0)	
**Educational attainment (n = 1,282)**					**<0.001[a]***
Basic secondary education certificate *(ISCED-2, Hauptschulabschluss)* (n = 62)	13 (21.0)	39 (62.9)	6 (9.7)	4 (6.5)	
Intermediate secondary education certificate *(ISCED-2, Realschulabschluss)* (n = 274)	66 (24.1)	171 (62.4)	34 (12.4)	3 (1.1)	
Entrance qualification for universities of applied sciences *(Fachhochschulreife)* (n = 121)	36 (29.8)	65 (53.7)	20 (16.5)	0 (0.0)	
General university entrance qualification *(Abitur)* (n = 181)	74 (40.9)	94 (51.9)	12 (6.6)	1 (0.6)	
Higher education degree (n = 612)	242 (39.5)	318 (52.0)	46 (7.5)	6 (1.0)	
No formal qualification (n = 3)	0 (0.0)	2 (66.7)	1 (33.3)	0 (0.0)	
Other qualification (n = 29)	8 (27.6)	12 (41.4)	8 (27.6)	1 (3.4)	
**Number of indicated diseases and/or disabilities (n = 959)[c]**					**0.023[d]***
1 (n = 200)	78 (39.0)	101 (50.5)	20 (10.0)	1 (0.5)	
2–3 (n = 440)	158 (35.9)	229 (52.0)	48 (10.9)	5 (1.1)	
4–5 (n = 207)	64 (30.9)	123 (59.4)	19 (9.2)	1 (0.5)	
≥6 (n = 112)	29 (25.9)	59 (52.7)	20 (17.9)	4 (3.6)	
**Membership Background (n = 1,315)**					0.143[b]
Affected individual (by disease or disability) (n = 994)	341 (34.3)	529 (53.2)	112 (11.3)	12 (1.2)	

	Self-rated digital skills				
	Very good, n (%)	Rather good, n (%)	Rather poor, n (%)	Very poor, n (%)	p-Value
Relative of an affected individual (n = 244)	85 (34.8)	142 (58.2)	14 (5.7)	3 (1.2)	
Other (n = 77)	22 (28.6)	45 (58.4)	10 (13.0)	0 (0.0)	
**Duration of PO Membership (n = 1,319)**					**0.029[a]***
Less than 1 year (n = 120)	44 (36.7)	71 (59.2)	5 (4.2)	0 (0.0)	
1–2 years (n = 155)	61 (39.4)	78 (50.3)	15 (9.7)	1 (0.6)	
3–5 years (n = 304)	121 (39.8)	148 (48.7)	32 (10.5)	3 (1.0)	
6–10 years (n = 257)	86 (33.5)	136 (52.9)	31 (12.1)	4 (1.6)	
More than 10 years (n = 483)	137 (28.4)	286 (59.2)	53 (11.0)	7 (1.4)	
**Voluntary work in PO (n = 1,308)**					0.820[d]
Yes (n = 484)	166 (34.3)	267 (55.2)	45 (9.3)	6 (1.2)	
No (n = 824)	280 (34.0)	445 (54.0)	90 (10.9)	9 (1.1)	

[a] p-values were computed using the Fisher-Freeman-Halton exact test because more than 20% of the expected cell frequencies were below 5 or the minimum expected frequency was below 1, making the Chi-square test inappropriate. Due to memory limitations in SPSS, the Fisher-Freeman-Halton exact test was calculated using the Monte Carlo method.

[b] p-values were computed using the Fisher-Freeman-Halton exact test (see footnote a for justification)

[c] Details regarding this variable can be found in the footnote to Table 1 in the manuscript.

[d] p-values were computed using the Chi-squared test.

* $p < 0.05$ was considered statistically significant.

Note: Percentages are based on valid responses and may not sum to 100% due to rounding.
