# Supplementary material for: Social factors associated with the development of e-health literacy: A mixed-methods study among patient organization members and representatives
Source: PLOS Digit Health. 2026 Aug 13;5(8):e0001569. doi: 10.1371/journal.pdig.0001569 (PMC13472428; doi:10.1371/journal.pdig.0001569)
Supplement: Supplementary file 7 [file pdig.0001569.s007.pdf]

CASE	MI03	ND04	ND05_01	ND05_02	ND05_03	ND05_04	ND05_05	ND05_06	ND05_07	ND05_08	PA06_03	SD02	SD03_01_	SD04	SD05_num_conditions_cat
5760	3	-1	2	3	1	3	1	1	1	2	.	1	.	1	.
3661	3	2	4	3	3	4	3	4	-1	2	.	1	4	-1	.
4245	2	3	3	3	3	3	3	3	2	3	.	1	5	4	.
6286	1	2	1	1	1	-1	1	1	1	1	1	1	.	1	1
6333	1	1	1	1	1	3	1	2	1	1	.	2	5	1	1
3804	1	2	1	3	-1	4	1	-1	3	-1	1	1	.	4	2
6368	1	1	2	3	3	3	3	1	1	1	.	1	2	5	2
6581	1	2	1	3	2	3	3	1	1	1	.	2	3	-1	3
2113	1	2	1	2	1	1	2	1	1	1	2	1	5	1	3
3470	1	1	1	-1	1	2	1	1	1	1	.	1	3	1	4
4996	1	2	2	2	2	2	2	3	1	1	.	2	4	3	4
4493	1	2	1	2	1	2	2	1	2	2	2	1	4	4	4
1682	3	2	1	3	4	-1	3	1	1	2	2	2	5	4	.
2111	3	3	3	3	3	3	3	2	-1	3	.	1	4	4	.
5697	2	2	1	1	1	2	2	1	1	1	1	1	4	1	.
5939	3	1	1	4	1	4	3	3	4	1	.	1	.	1	.
741	2	1	1	2	3	3	3	1	1	1	1	2	4	2	.
933	2	2	1	1	1	4	1	2	3	4	.	1	.	1	.
2077	2	2	-1	2	3	4	3	3	3	3	1	-1	.	-1	.
2601	2	2	1	3	2	3	3	2	1	2	1	1	4	2	.
3125	3	3	1	2	1	3	2	1	1	3	1	1	5	1	.
5311	2	1	1	1	1	3	2	1	1	1	.	2	4	1	.
5345	2	2	1	1	2	3	4	3	1	1	.	1	3	3	.
5360	3	1	1	2	1	3	1	1	2	1	1	2	5	1	.
6117	2	1	1	3	2	3	4	2	1	1	.	1	4	4	.
6881	2	2	1	2	1	3	1	1	1	1	.	1	3	1	.
1715	3	2	2	3	2	2	3	1	1	1	1	1	5	4	.
2613	2	2	1	1	1	3	1	1	1	1	.	1	3	2	.
2722	3	1	1	2	2	2	1	2	1	1	1	1	3	3	.
2746	2	2	1	2	1	3	1	1	1	1	.	1	3	2	.
4026	2	3	3	3	2	1	2	1	4	4	.	1	4	3	.
4092	3	1	1	1	-1	2	4	1	1	1	.	1	4	4	.
5351	2	1	1	1	1	3	2	1	1	1	.	1	.	2	.

5942	3	2	1	3	2	2	2	1	1	1	.	2	5	5	.
5966	3	1	4	3	3	4	4	1	1	1	.	1	4	2	.
6193	2	2	1	2	1	3	3	1	1	1	.	2	5	2	.
6788	3	2	2	2	3	3	4	1	4	1	.	2	5	1	.
6799	2	4	1	4	4	1	3	1	3	4	2	1	3	1	.
6879	3	1	3	3	2	3	2	3	2	2	.	2	4	4	.
3260	3	2	2	2	2	3	2	3	2	3	2	2	4	1	.
4933	3	2	3	2	2	2	3	1	4	2	2	2	5	8	.
5023	2	2	1	3	2	4	2	4	3	2	.	1	2	1	.
5317	2	2	2	2	4	4	2	3	1	1	.	1	3	4	.
5460	3	2	1	4	3	4	1	1	1	1	.	2	4	1	.
6039	3	3	1	4	4	3	3	2	2	3	2	1	5	1	.
6046	2	2	1	3	3	1	3	1	1	1	.	1	4	1	.
6595	2	1	4	3	3	2	4	3	1	1	.	1	3	3	.
6778	2	2	1	3	1	4	2	1	1	1	1	1	4	1	.
6843	2	2	1	3	2	-1	3	1	1	1	2	1	4	4	.
748	2	1	1	3	1	2	2	1	1	1	2	1	3	1	.
752	2	2	1	1	1	2	2	1	3	2	1	1	5	1	.
888	3	2	1	2	2	3	2	2	3	2	1	1	5	8	.
2909	3	2	1	2	1	3	1	1	2	2	1	1	5	1	.
3200	2	2	2	2	2	2	2	1	2	2	2	1	4	3	.
3507	2	2	1	3	2	4	3	2	3	2	.	2	4	1	.
3676	2	2	1	2	2	3	1	2	1	1	.	1	2	2	.
3929	2	2	2	3	3	4	3	-1	3	3	.	2	5	1	.
3972	2	1	1	1	1	3	1	1	1	1	.	1	2	1	.
4215	2	3	1	3	3	3	2	2	2	2	.	1	4	2	.
4651	2	2	3	3	3	3	2	3	1	3	.	1	3	1	.
4944	3	1	1	2	2	2	3	1	1	1	1	2	4	4	.
5299	2	2	2	3	2	1	2	2	2	2	.	2	5	1	.
5307	2	2	1	1	1	3	3	1	1	1	1	1	3	4	.
5364	2	3	1	4	2	3	2	1	2	2	2	1	4	1	.
5474	2	2	1	3	3	3	4	2	2	3	2	1	5	2	.
5652	2	2	1	2	2	4	4	1	1	3	2	1	4	-1	.
6140	3	-9	1	4	2	4	4	4	2	3	.	1	5	5	.
6195	2	3	1	3	3	3	2	2	2	2	2	1	5	1	.
6300	2	2	2	3	2	2	3	1	1	1	2	2	5	5	.

6315	2	2	2	3	2	3	2	2	2	3	2	1	5	4	.
1547	2	1	1	3	1	2	2	1	1	1	.	1	4	1	.
2497	2	2	1	1	1	3	2	1	1	1	.	1	4	4	.
2823	2	2	1	2	1	2	2	1	1	1	.	1	2	1	.
3160	-1	2	1	2	1	3	3	2	2	2	.	1	5	1	.
4288	3	2	1	3	2	3	2	3	3	3	1	2	4	1	.
5152	3	2	1	-1	3	4	3	2	2	2	.	1	4	1	.
5277	2	1	1	3	3	1	2	1	1	1	1	1	3	1	.
5321	2	2	1	2	2	2	2	2	2	3	2	1	2	4	.
5563	2	1	1	1	1	2	3	1	1	1	.	1	3	1	.
6763	3	1	1	1	1	1	1	1	1	1	1	2	5	1	.
2143	2	2	2	3	1	4	3	1	1	1	.	2	4	1	.
2536	2	1	1	2	3	2	4	1	1	1	.	1	2	1	.
2677	2	2	1	3	2	2	2	1	1	2	.	1	4	2	.
4830	2	2	2	2	2	2	3	1	2	2	1	1	4	1	.
5738	2	2	2	2	2	3	2	2	2	2	2	2	4	1	.
746	2	1	4	4	2	2	2	2	2	2	2	1	.	1	.
5713	2	2	2	1	1	3	2	2	1	1	2	1	3	2	.
6113	2	2	1	3	2	4	1	1	1	3	2	1	2	1	.
6737	2	2	2	2	2	3	2	2	1	2	.	1	4	2	.
2221	2	2	1	3	1	4	2	1	1	2	.	1	2	3	.
6066	2	2	1	3	3	2	3	3	1	2	.	2	3	4	.
6739	2	2	1	3	2	2	2	2	2	2	.	1	5	1	.
6903	2	1	1	2	3	3	2	1	3	1	.	1	3	3	.
1060	2	1	1	1	1	1	1	1	1	1	.	1	4	1	.
4688	2	1	3	2	2	1	1	1	1	1	.	2	5	1	.
3575	2	1	2	2	2	2	2	1	1	2	.	2	5	1	.
5541	2	2	1	1	1	1	1	1	1	1	2	-1	.	-1	.
1326	2	2	1	3	3	3	2	3	1	2	.	1	3	1	.
6232	3	2	4	3	3	3	3	3	3	3	.	1	5	5	.
1206	3	2	2	3	3	2	3	4	1	3	.	1	4	8	.
5981	3	2	3	4	3	4	3	2	1	2	.	1	.	-1	.
930	1	-1	-9	-9	-9	-9	-9	-9	-9	-9	.	-9	.	-1	-2
4150	1	1	2	3	3	4	3	2	2	2	.	1	.	-1	-2
4384	1	2	1	2	3	3	2	2	2	2	1	1	4	3	-2
4583	1	1	1	1	1	4	2	1	1	1	.	-1	.	-1	-2

6724	1	2	2	3	3	3	3	3	3	3	.	1	.	4	-2
4316	1	2	2	2	2	2	2	2	2	2	1	2	4	4	-2
2312	1	2	1	2	1	4	2	1	3	3	1	1	4	2	-2
6755	1	2	1	3	3	2	3	2	2	2	2	2	5	-1	-2
2331	1	1	1	2	1	2	1	1	1	1	1	1	1	1	1
3618	1	2	1	1	1	2	1	1	1	1	1	1	4	1	1
4111	1	3	3	3	3	2	4	2	4	3	2	2	5	3	1
2445	1	2	2	1	1	2	2	1	1	1	.	2	4	1	1
3841	1	3	1	4	4	1	3	3	2	2	1	1	.	-1	1
4432	1	1	3	2	3	3	3	4	3	4	.	1	4	3	1
4871	1	1	4	2	1	3	1	2	2	2	2	1	4	1	1
6705	1	3	1	4	3	4	4	2	2	2	.	-1	5	8	1
6793	1	2	1	3	3	3	4	-1	4	3	2	1	.	4	1
1156	1	2	1	1	1	1	1	1	1	1	2	2	2	1	1
2739	1	1	1	2	2	1	1	1	1	1	.	1	2	1	1
3798	1	2	1	3	2	3	3	1	1	1	.	1	4	1	1
3932	1	1	3	2	2	4	1	1	1	2	.	1	4	2	1
6773	1	2	1	3	1	4	3	2	1	1	2	1	4	2	1
1027	1	2	2	3	3	2	3	1	1	2	2	1	4	4	1
1426	1	2	2	3	2	4	2	3	4	2	1	2	5	1	1
1595	1	2	1	2	2	3	3	2	2	2	1	1	3	4	1
2212	1	2	3	2	2	4	2	3	4	2	.	2	5	1	1
3834	1	2	1	2	2	3	2	2	1	2	.	1	3	5	1
4466	1	1	1	3	1	2	3	1	1	2	.	2	3	1	1
4625	1	2	2	2	1	4	2	1	1	1	1	1	4	4	1
4883	1	2	1	2	2	3	3	2	1	2	.	1	4	1	1
5980	1	1	1	1	1	1	1	1	1	1	1	2	4	1	1
6297	1	3	2	3	3	2	4	3	4	3	2	1	3	1	1
6854	1	3	-1	3	3	-1	-1	1	2	-1	.	2	5	1	1
1639	1	1	1	4	1	4	3	1	1	1	.	2	5	1	1
2687	1	1	1	2	1	3	2	1	1	1	.	1	.	1	1
3096	1	1	1	1	1	2	1	1	1	1	.	2	4	1	1
3199	1	2	1	1	1	3	1	1	1	1	.	1	4	1	1
3751	1	2	1	1	1	2	1	1	1	1	1	1	4	1	1
4735	1	4	-1	3	-1	3	3	-1	3	3	2	1	5	1	1
6047	1	1	1	2	1	1	1	1	1	1	.	2	5	1	1

6831	1	1	2	2	2	2	2	2	2	2	.	1	3	1	1
776	1	1	1	3	2	2	2	2	2	2	1	1	4	1	1
1518	1	1	1	3	3	4	4	1	1	1	.	2	3	1	1
3493	1	1	1	1	1	1	2	1	1	1	.	1	5	1	1
3982	1	2	1	2	2	1	1	2	1	1	2	1	4	1	1
4469	1	2	1	3	3	2	3	1	1	1	2	2	4	4	1
6308	1	3	3	4	3	2	4	2	2	3	.	2	5	1	1
1597	1	2	1	2	3	2	3	1	1	1	.	1	3	1	1
1695	1	2	1	3	3	3	2	2	1	2	.	2	5	1	1
4820	1	1	4	2	2	2	2	1	1	1	1	1	4	2	1
6442	1	2	1	1	2	1	3	1	1	1	1	1	3	4	1
2694	1	1	2	2	2	2	2	2	2	2	.	1	3	1	1
4718	1	-1	-9	-9	-9	-9	-9	-9	-9	-9	1	2	5	3	1
6185	1	2	1	1	1	4	1	1	4	1	.	1	5	1	1
1634	1	2	3	4	3	4	4	4	4	4	2	2	5	-1	2
1667	1	2	2	2	2	3	2	1	1	1	.	2	.	1	2
4072	1	3	2	2	2	3	1	4	4	2	.	2	5	1	2
4409	1	3	-1	4	3	3	3	2	3	3	1	1	5	1	2
6032	1	2	2	2	3	4	3	3	3	3	.	2	5	3	2
6666	1	3	3	4	3	3	2	2	3	3	2	1	5	4	2
6922	1	2	1	3	2	3	2	1	1	1	.	1	5	1	2
976	1	2	1	3	3	2	4	2	2	2	2	2	.	-1	2
1756	1	2	1	1	1	1	1	1	1	1	2	1	4	2	2
1930	1	1	1	2	2	3	2	2	1	1	.	2	3	1	2
4323	1	2	1	3	1	4	3	4	1	1	1	1	4	4	2
4959	1	1	3	3	2	1	2	1	2	3	.	2	4	1	2
5207	1	1	1	1	2	3	2	1	1	1	.	1	3	1	2
6171	1	2	3	3	3	4	3	4	4	4	2	2	4	4	2
6655	1	2	1	2	1	1	1	3	1	1	.	2	5	1	2
3818	1	2	1	3	3	2	2	1	1	1	.	2	4	1	2
4077	1	2	1	3	3	2	2	3	2	3	2	1	4	-1	2
4230	1	2	1	2	3	3	3	2	3	2	2	1	4	1	2
4677	1	1	-1	2	1	3	1	1	1	1	1	1	2	1	2
5953	1	2	2	2	2	3	2	2	2	2	.	2	5	1	2
6057	1	2	1	2	3	2	2	1	1	1	.	1	1	1	2
6816	1	2	3	3	2	2	3	2	3	3	1	2	5	1	2

1560	1	2	2	3	2	3	3	1	2	2	.	2	5	3	2
1567	1	2	1	2	1	1	1	1	2	1	1	1	4	2	2
1779	1	1	1	2	1	2	2	1	1	1	1	1	4	1	2
2755	1	2	2	2	2	3	2	2	1	1	2	1	3	4	2
3683	1	2	1	2	2	2	3	1	4	4	.	1	4	1	2
3749	1	2	1	2	2	1	2	1	1	2	1	2	5	4	2
4904	1	2	1	4	4	4	4	2	2	1	.	1	4	5	2
5501	1	3	1	2	2	3	1	1	3	3	2	1	2	1	2
5906	1	1	1	2	1	3	2	2	1	1	1	2	4	1	2
6200	1	1	1	1	1	3	1	1	1	1	1	2	4	1	2
6468	1	2	1	2	2	2	2	1	1	2	.	1	4	4	2
1185	1	1	1	2	2	3	3	2	1	1	.	2	4	1	2
2805	1	2	1	2	1	3	3	1	1	1	.	2	4	1	2
3752	1	2	1	2	2	1	2	1	1	2	1	1	5	4	2
3791	1	2	1	3	3	4	3	2	2	2	.	2	5	1	2
5813	1	1	2	3	1	3	1	1	1	1	.	2	4	1	2
5855	1	1	1	2	1	3	1	1	1	1	1	1	4	2	2
5930	1	3	-1	3	4	3	2	1	-1	3	.	2	5	1	2
6172	1	1	4	3	3	3	3	2	1	1	1	1	5	1	2
6216	1	1	1	1	1	2	1	1	1	1	.	1	4	1	2
6466	1	1	1	2	1	3	1	1	1	1	.	1	4	4	2
1050	1	2	2	3	3	2	3	3	2	3	.	2	5	3	2
3784	1	3	2	4	3	4	3	3	3	4	2	1	4	1	2
3830	1	2	1	3	3	2	2	2	2	2	.	1	2	1	2
5153	1	2	1	3	3	3	3	2	2	2	.	1	4	2	2
5268	1	1	1	2	1	3	3	2	1	1	.	2	5	1	2
5992	1	3	3	3	3	3	4	2	1	2	.	2	5	1	2
3014	1	2	1	2	2	1	2	1	1	1	.	2	4	1	2
3448	1	2	3	3	2	3	2	1	1	1	2	1	5	5	2
4963	1	3	1	3	3	2	4	3	2	3	2	1	5	2	2
6277	1	1	2	4	3	2	3	1	1	1	1	1	4	4	2
824	1	2	2	3	3	4	4	1	1	1	.	1	4	4	2
4485	1	2	1	2	3	1	3	2	1	1	.	1	3	1	2
5062	1	2	2	3	3	3	4	1	1	1	.	1	4	1	2
5516	1	1	1	1	1	2	2	1	1	1	.	2	.	1	2
5993	1	1	1	1	1	1	1	1	1	1	.	1	5	3	2

3432	1	4	2	4	4	1	2	3	4	4	2	1	5	1	2
6907	1	2	3	2	2	1	3	1	1	1	1	2	5	2	2
6909	1	2	1	2	2	3	3	3	2	2	1	1	5	4	2
1790	1	2	1	3	1	4	1	1	1	3	1	1	5	2	2
2063	1	1	1	1	1	3	1	1	1	1	.	1	4	1	2
6317	1	2	1	4	4	3	4	1	1	3	.	1	.	4	2
6729	1	-1	2	3	2	2	2	2	2	2	1	1	5	4	2
1815	1	1	1	3	2	3	-1	1	1	1	.	2	4	1	2
3809	1	3	3	3	3	4	4	4	3	3	.	1	.	-1	2
3899	1	4	4	4	4	4	4	3	4	4	1	1	4	1	2
4685	1	3	-1	2	-1	-1	4	-1	4	4	.	1	4	1	2
6529	1	2	2	3	4	3	1	1	1	2	1	1	2	1	2
3627	1	2	1	2	2	2	2	1	2	2	.	2	5	1	2
3867	1	2	1	3	2	3	1	1	1	2	.	1	3	1	2
3968	1	2	1	2	2	3	3	4	1	1	.	2	5	1	2
5611	1	1	-1	2	2	4	1	1	1	1	.	1	4	1	2
3812	1	2	1	3	2	3	2	1	2	2	.	1	4	4	2
4835	1	1	1	1	1	3	1	1	1	1	.	2	4	1	2
5056	1	3	-1	3	2	4	3	4	4	4	.	1	4	1	2
6293	1	1	1	4	4	4	2	2	4	1	1	1	4	1	2
1466	1	2	1	2	1	3	1	1	1	1	2	1	2	2	2
1754	1	2	2	3	3	4	4	2	2	2	.	2	5	3	2
2158	1	2	1	3	3	4	4	1	1	3	.	-1	.	-1	2
2422	1	3	1	2	3	4	2	3	4	4	2	1	.	2	2
3482	1	1	2	2	2	2	3	-1	2	2	.	2	4	4	2
5641	1	2	1	2	2	2	3	1	1	1	.	2	4	1	2
6177	1	2	2	3	2	3	3	2	1	2	1	2	5	4	2
6786	1	2	2	3	4	4	2	2	2	2	2	1	5	4	2
2507	1	2	1	2	1	4	3	3	1	4	.	2	5	-1	2
4315	1	1	1	3	3	4	1	4	3	1	2	1	4	1	2
4750	1	2	4	3	2	2	2	1	1	3	.	1	4	2	2
6396	1	1	1	1	1	1	1	1	1	1	.	1	3	2	2
6930	1	2	-1	1	1	3	2	1	1	1	2	1	4	3	2
1075	1	2	1	3	2	3	2	2	2	2	.	2	4	4	2
5212	1	3	1	3	2	4	2	1	3	2	1	1	5	2	2
5917	1	1	1	1	1	2	2	1	1	1	1	2	4	1	2

1387	1	2	2	2	3	2	2	3	2	2	.	1	4	5	2
1532	1	2	2	2	2	2	1	1	1	1	.	1	4	4	2
3612	1	1	2	4	3	2	2	2	1	1	.	2	5	1	2
4962	1	2	1	3	2	4	2	1	1	1	.	1	4	4	2
5717	1	2	3	3	3	4	3	4	2	3	.	1	4	4	2
6715	1	2	2	3	1	3	2	1	1	2	1	2	4	1	2
1601	1	2	2	1	2	-1	2	3	1	2	1	1	5	5	2
3915	1	1	1	1	1	2	2	2	1	1	.	1	4	2	2
1127	1	2	-1	-1	-1	4	4	-1	-1	-1	.	2	4	4	2
4066	1	2	3	4	3	4	4	2	2	3	2	2	5	3	2
2786	1	1	1	1	1	3	2	1	1	1	1	1	5	1	3
4961	1	2	1	3	3	4	3	2	2	2	.	2	4	4	3
6094	1	2	3	2	2	4	3	3	3	2	.	1	.	1	3
6650	1	1	1	1	1	1	1	1	1	1	.	2	5	1	3
1632	1	2	1	2	2	2	2	1	1	1	.	2	5	1	3
5718	1	2	2	2	1	2	2	1	1	1	.	1	5	4	3
6067	1	1	1	3	2	1	3	1	1	1	1	1	4	2	3
6775	1	2	1	3	1	1	3	3	1	1	.	2	5	5	3
6826	1	2	3	3	2	2	3	1	2	2	.	2	4	4	3
5035	1	2	4	1	1	-1	1	1	1	1	.	1	4	3	3
6107	1	2	2	3	2	2	4	3	1	2	2	1	4	1	3
1024	1	2	1	2	2	1	3	1	1	1	.	2	5	4	3
1055	1	2	3	3	1	3	2	1	1	1	.	1	5	3	3
1080	1	1	1	3	3	2	3	1	1	1	.	2	5	1	3
2792	1	1	2	4	3	4	2	2	1	1	.	2	5	1	3
4227	1	2	-1	3	3	3	2	2	-1	3	.	1	5	1	3
4454	1	3	2	3	3	4	3	2	1	2	.	1	5	1	3
6082	1	1	1	2	2	1	2	2	1	2	.	2	5	2	3
5240	1	1	1	3	2	2	2	1	1	1	.	1	3	4	3
6180	1	2	3	3	2	2	3	2	2	2	2	1	5	4	3
1764	1	2	1	3	2	2	3	2	1	2	.	1	5	4	3
3831	1	1	1	2	2	2	2	1	1	1	.	2	5	1	3
787	1	3	4	4	3	3	4	4	3	3	2	1	5	3	3
4473	1	2	1	1	1	2	1	1	1	1	.	1	5	4	3
1803	1	2	3	4	3	2	2	1	3	2	1	1	5	1	3
3511	1	2	2	2	2	3	3	2	2	2	.	1	5	1	3

1557	1	1	1	1	1	4	4	1	1	1	.	1	.	3	3
3443	1	2	-1	4	4	4	4	3	4	4	2	1	5	8	3
3918	1	1	1	1	1	1	1	1	1	1	.	2	4	1	3
2666	1	3	1	4	4	1	2	1	1	3	2	1	2	1	3
4361	1	2	-1	3	3	4	2	3	2	2	.	1	5	4	3
4694	1	2	1	4	3	2	3	1	3	3	2	1	4	4	3
6530	1	1	1	1	2	1	2	1	1	1	.	1	4	2	3
6583	1	3	1	2	3	3	2	3	2	3	1	1	3	3	3
1223	1	2	3	3	2	4	2	1	3	1	1	2	4	1	3
1427	1	1	4	4	3	4	4	2	1	2	1	2	3	4	3
1561	1	2	1	3	2	4	3	2	2	2	.	2	5	4	3
3594	1	1	1	2	2	1	1	1	1	1	.	1	4	2	3
4194	1	2	1	3	3	4	4	1	1	2	2	1	5	3	3
6662	1	2	2	3	4	2	2	2	3	2	1	1	4	1	3
2257	1	1	1	2	1	3	1	1	1	1	.	1	1	1	3
6721	1	1	1	3	3	3	2	1	1	1	.	2	4	1	3
3799	1	1	1	2	2	3	1	3	1	1	1	1	4	4	3
6448	1	1	1	1	1	1	1	1	1	1	.	1	3	1	3
1575	1	1	1	1	1	2	3	1	1	1	.	2	.	1	3
3090	1	1	1	3	4	4	3	2	1	1	1	1	1	3	3
4281	1	1	3	3	2	1	3	3	1	1	.	2	3	4	3
6722	1	2	1	3	3	3	3	2	2	2	.	1	4	1	3
3813	1	2	1	2	1	2	1	1	1	1	.	2	5	4	3
4038	1	2	2	3	2	3	3	1	-1	-1	.	1	3	2	3
1696	1	-1	-1	4	4	4	-1	-1	-1	-1	1	2	5	4	3
1443	1	1	1	1	1	3	3	1	1	1	1	1	4	-1	3
3738	1	3	2	3	1	3	1	3	3	3	1	1	4	3	3
4305	1	2	1	3	3	3	3	3	2	2	.	1	4	2	4
1306	1	2	1	2	1	1	3	1	2	2	2	1	5	4	4
3848	1	3	3	3	3	4	4	3	4	3	2	2	5	3	4
6009	1	2	1	3	3	3	3	1	2	2	1	1	4	1	4
6170	1	2	2	4	4	3	1	2	4	3	2	1	5	1	4
6463	1	2	1	2	1	3	3	2	2	1	2	2	4	1	4
2024	1	1	1	2	2	3	3	1	1	1	1	1	5	4	4
3427	1	2	1	2	2	3	1	1	1	1	1	1	4	3	4
3678	1	3	1	4	3	3	3	2	2	3	.	2	.	1	4

5192	1	2	1	3	2	4	1	1	1	1	.	2	4	1	4
6116	1	2	2	2	2	4	1	3	3	3	.	1	4	2	4
5686	1	2	1	2	2	3	3	1	2	1	.	1	4	4	4
1505	1	3	2	3	2	3	2	3	2	3	.	1	4	3	4
4502	1	2	2	3	3	2	3	2	2	2	1	2	5	4	4
3195	1	2	2	3	2	1	2	4	1	2	.	1	3	3	4
6900	1	1	2	3	3	4	3	3	1	1	.	1	5	2	4
6023	1	1	1	1	1	1	1	1	1	1	1	2	5	1	4
3833	1	4	1	3	3	3	2	2	3	3	2	2	4	4	4
6550	1	3	3	3	3	2	3	3	4	3	2	2	4	4	4
2809	1	1	1	2	1	1	3	2	1	1	.	1	4	1	4
4733	1	3	3	4	4	4	4	3	2	3	.	1	4	1	4
1699	1	1	1	4	2	1	1	1	1	1	2	1	4	2	4
3978	1	1	1	1	1	3	1	1	1	1	.	1	4	2	4
5132	1	3	3	2	3	4	1	4	4	2	.	1	4	4	4
4211	1	1	1	1	1	4	1	1	1	1	1	-1	.	1	4
2355	1	-1	4	4	4	4	3	4	4	4	1	1	5	1	4
6915	1	2	3	3	3	4	4	3	2	2	2	1	5	5	4
4307	1	2	3	3	2	4	2	1	-1	2	.	1	5	1	4
4478	1	2	2	2	3	3	2	2	2	2	.	1	5	4	4
874	1	2	2	3	3	2	1	1	2	1	.	2	2	2	4
1724	2	2	1	3	3	2	4	1	1	2	.	2	5	1	.
2866	3	3	1	2	1	4	4	3	1	1	.	-1	.	-1	.
4028	2	1	1	1	1	1	1	1	1	1	1	1	2	1	.
4096	2	2	1	2	2	3	3	1	2	2	1	1	3	4	.
4403	2	2	1	3	1	3	4	1	1	3	.	1	.	2	.
6186	3	2	1	3	3	2	3	2	1	1	.	2	3	1	.
6330	2	2	3	4	4	2	4	2	1	2	2	1	2	5	.
2247	2	2	2	2	2	2	2	2	1	1	1	1	4	4	.
3819	2	1	1	1	2	3	2	1	1	1	1	2	5	2	.
3943	2	2	1	2	2	3	2	2	2	1	.	1	5	1	.
3966	3	1	1	1	1	1	2	1	1	1	1	1	2	4	.
3976	2	2	1	3	3	4	2	1	1	1	.	1	1	2	.
3987	2	2	1	3	1	3	2	1	1	1	1	1	.	4	.
4080	2	2	1	2	2	3	1	2	2	3	.	1	3	1	.
4082	2	2	1	2	2	1	1	1	1	1	1	1	4	1	.

4374	2	2	1	3	2	3	2	1	1	1	2	1	4	1	.
4455	2	2	1	4	3	2	1	3	1	1	1	1	3	1	.
5248	2	1	1	4	3	1	4	1	1	1	2	1	2	3	.
5270	2	1	1	3	1	2	2	1	1	1	.	2	2	3	.
5276	2	2	1	3	3	4	3	2	2	2	2	1	4	1	.
5572	2	2	1	2	2	2	3	3	1	1	.	1	4	4	.
5650	2	2	1	3	2	4	2	1	3	1	2	1	4	1	.
5699	2	2	1	2	2	2	2	1	2	2	1	1	4	2	.
5750	2	2	1	3	2	2	1	2	1	1	1	1	4	1	.
5774	2	2	2	2	3	2	2	1	1	1	1	1	3	1	.
6608	2	2	2	4	2	1	1	1	1	1	1	2	4	4	.
6732	2	4	4	4	4	4	4	4	4	4	2	1	5	5	.
1668	3	1	1	2	1	2	1	1	1	1	1	2	5	1	.
2187	2	1	1	1	1	1	2	1	1	1	.	1	2	3	.
2193	2	1	1	2	1	2	1	3	1	1	1	2	3	1	.
2213	2	1	1	1	1	1	1	1	1	1	1	1	4	1	.
2216	2	2	1	3	2	3	3	2	2	2	2	1	3	2	.
2246	2	1	1	2	1	4	1	1	1	1	.	2	2	1	.
2354	2	1	1	3	2	4	3	1	1	1	2	1	.	1	.
2438	2	1	1	4	3	1	3	1	1	1	2	1	2	4	.
2558	2	1	1	2	1	2	2	1	1	1	1	1	3	4	.
2575	2	1	1	1	1	2	2	1	1	1	.	1	4	4	.
2745	2	2	1	3	3	2	2	1	2	1	1	2	3	4	.
2853	3	2	2	2	3	3	3	1	1	1	2	1	4	1	.
3698	2	1	1	3	2	2	3	1	1	1	1	1	1	3	.
3911	2	1	1	2	1	2	2	1	1	1	1	2	4	1	.
3971	2	2	1	3	2	1	3	1	3	3	.	1	2	1	.
3980	2	2	1	2	2	3	1	1	1	1	.	1	2	3	.
4458	2	2	-1	3	3	4	3	1	2	1	1	2	4	1	.
5150	2	1	3	1	1	1	2	1	1	1	.	1	2	4	.
5184	2	2	1	3	1	4	1	1	1	1	1	2	4	1	.
5293	2	1	2	2	2	2	3	2	2	2	2	2	3	4	.
5328	2	1	3	3	-1	3	-1	1	1	1	1	2	4	4	.
5344	2	2	3	3	4	3	3	1	1	3	.	1	3	2	.
5372	2	2	1	4	3	4	4	4	2	2	1	1	4	5	.
5400	2	2	1	2	2	3	2	1	1	2	1	2	4	2	.

5457	2	3	-1	3	2	3	3	1	1	3	2	1	5	5	.
5532	2	1	1	2	2	3	2	1	1	1	1	1	2	1	.
5599	2	2	1	2	1	2	2	1	1	1	1	1	4	4	.
5602	2	2	1	3	2	2	2	1	1	1	.	1	2	1	.
5612	2	2	3	2	1	3	2	1	2	3	1	1	4	2	.
5614	2	3	-1	4	4	3	3	4	2	3	2	-1	4	4	.
5769	2	-1	1	2	1	3	2	1	1	1	2	-1	.	-1	.
5905	2	2	2	2	2	2	2	2	2	2	1	1	4	4	.
6008	2	1	2	4	2	3	2	2	1	1	.	-1	2	1	.
6291	2	2	1	2	2	2	2	2	2	2	2	1	5	4	.
6359	2	3	2	3	2	3	3	2	2	3	2	1	5	4	.
6745	2	2	1	3	3	2	2	4	1	1	1	1	3	2	.
893	2	2	1	3	2	4	2	1	1	1	.	1	2	1	.
1528	3	3	2	3	1	4	1	1	1	3	.	1	4	-1	.
1531	3	3	2	3	2	4	2	1	1	3	2	1	4	-1	.
1776	2	1	1	2	1	2	2	1	1	1	.	1	4	4	.
2260	2	1	1	3	3	2	3	1	1	2	.	1	2	1	.
2540	2	2	1	3	2	3	3	2	2	4	2	1	3	1	.
2906	3	2	1	4	3	2	3	1	3	3	2	1	3	1	.
3119	2	1	1	1	1	1	1	1	1	1	1	1	4	1	.
3967	2	1	1	2	3	1	3	3	1	1	2	1	2	1	.
3970	2	2	1	3	3	4	2	3	2	2	2	1	4	1	.
3990	2	1	1	2	1	2	3	2	2	2	.	1	3	1	.
4103	2	2	1	3	2	4	3	4	3	2	.	1	3	4	.
4856	-1	2	2	3	3	2	3	2	2	2	2	2	5	4	.
5234	2	3	2	3	3	3	4	1	1	3	1	1	5	1	.
5257	2	2	2	2	2	3	2	1	1	1	.	2	5	2	.
5349	2	2	2	2	2	2	2	2	2	2	.	1	4	1	.
5562	2	2	1	2	2	2	2	3	2	2	1	1	2	4	.
5676	2	2	1	1	1	1	1	1	1	1	2	1	4	1	.
5693	3	2	2	2	3	4	1	1	1	1	1	1	4	1	.
5741	2	2	1	2	2	3	2	1	1	1	2	1	2	3	.
6735	2	1	1	2	1	4	2	1	1	1	.	1	4	1	.
1424	2	2	3	3	3	4	2	2	1	2	2	1	5	1	.
1631	2	3	1	3	2	4	1	3	2	4	2	1	4	1	.
1902	2	2	2	3	2	3	3	2	1	2	.	1	2	1	.

1909	2	2	1	3	2	3	3	-1	2	3	2	1	3	1	.
2096	2	2	1	1	1	3	1	1	3	1	.	2	.	1	.
2155	2	1	1	1	3	3	2	1	1	1	.	2	3	1	.
2185	2	2	1	2	1	3	1	1	1	1	.	1	2	3	.
2194	2	1	1	1	1	1	1	1	1	1	.	1	2	3	.
2397	2	1	1	1	1	4	1	1	1	1	1	1	3	1	.
2468	2	2	1	3	2	2	2	3	1	3	.	1	2	4	.
2869	3	1	1	3	1	4	2	1	1	1	.	1	4	1	.
3028	2	2	1	2	2	2	2	3	1	1	.	1	4	1	.
3958	2	1	1	3	2	3	2	1	1	1	1	1	1	4	.
4004	2	1	1	2	1	1	3	1	1	1	.	2	2	1	.
4031	2	1	1	3	2	3	1	1	1	2	.	1	3	1	.
4044	2	2	1	2	1	2	2	1	1	1	.	2	2	2	.
4522	2	2	1	3	2	4	2	1	1	2	1	2	4	1	.
4809	2	2	3	3	1	2	1	2	1	1	1	1	1	2	.
5233	2	3	1	3	3	4	2	3	2	2	1	1	4	1	.
5287	2	2	1	2	1	3	3	1	1	1	1	1	4	1	.
5370	2	2	1	2	3	3	2	1	2	2	2	1	4	2	.
5405	2	2	1	3	2	3	3	1	1	1	1	1	3	1	.
5473	2	2	1	2	2	3	3	2	1	3	.	1	2	1	.
5869	2	2	2	3	3	3	3	2	1	1	.	1	3	1	.
6501	2	2	1	1	1	1	1	1	1	1	.	2	1	5	.
6777	2	2	2	2	2	3	2	1	3	3	.	2	5	5	.
6935	2	1	1	3	3	4	3	2	1	1	1	1	4	1	.
1321	3	2	1	2	3	4	2	1	1	1	1	2	3	1	.
1761	3	2	2	2	2	4	3	2	2	2	.	2	5	1	.
2198	2	2	1	2	2	4	2	2	1	2	1	1	3	1	.
2211	2	2	1	2	1	1	1	1	1	1	.	1	3	4	.
2371	2	1	1	3	2	3	3	3	1	1	2	1	3	4	.
2744	2	2	2	3	2	2	2	1	1	1	.	1	2	1	.
4017	2	2	1	3	2	2	3	2	2	2	1	1	4	2	.
4146	2	1	1	1	1	3	1	1	1	1	.	1	.	8	.
4151	2	2	1	2	1	4	2	1	1	1	.	1	3	1	.
4258	3	2	1	3	2	2	3	1	1	2	.	1	4	1	.
4783	2	1	1	1	1	1	1	1	1	1	.	1	2	2	.
5452	2	2	1	2	2	2	2	1	1	1	.	1	3	2	.

5643	2	1	2	3	3	2	2	1	1	1	1	1	2	1	.
5823	2	1	2	3	3	4	3	3	2	2	1	1	2	-1	.
6726	2	2	1	1	3	4	2	1	1	2	.	1	.	1	.
2087	2	1	1	3	2	2	3	2	1	1	1	1	3	1	.
2110	2	1	2	4	4	2	3	1	4	1	.	1	4	1	.
2192	2	1	1	2	2	3	3	2	2	3	.	1	2	2	.
2605	3	2	1	2	2	3	2	2	2	3	2	1	4	4	.
3956	2	1	4	4	4	4	3	3	1	1	2	1	3	1	.
3985	2	1	1	2	2	1	1	1	1	1	1	2	3	8	.
4033	2	2	1	2	1	3	1	1	1	1	.	1	4	2	.
4093	2	1	1	2	1	-9	3	1	1	1	.	1	.	2	.
4124	2	-1	2	3	3	3	3	2	2	2	2	1	3	1	.
4147	3	2	1	1	1	3	1	1	1	1	.	1	3	1	.
5238	2	2	1	3	3	2	2	1	1	1	1	1	3	1	.
5279	3	1	1	2	1	2	1	1	1	1	.	2	4	4	.
5524	3	3	1	2	3	3	2	4	4	4	.	1	4	5	.
5561	2	1	1	3	2	4	1	3	1	1	1	1	3	1	.
791	2	2	1	2	3	1	3	2	1	1	.	1	5	3	.
913	2	2	1	1	1	1	4	1	1	1	.	1	3	4	.
2210	2	1	1	3	2	2	2	2	1	1	1	1	3	2	.
3058	2	1	1	4	2	2	3	1	1	1	.	1	4	1	.
4013	2	3	2	3	3	3	4	3	2	2	.	1	2	4	.
4656	2	1	2	2	1	1	1	1	1	1	.	2	5	1	.
5416	-1	2	1	2	2	3	2	2	2	1	1	-1	4	1	.
5603	2	2	1	2	2	3	3	2	1	2	.	1	1	2	.
5702	3	1	1	2	1	1	1	1	1	1	.	1	4	2	.
6337	2	2	1	2	3	4	1	1	1	1	1	1	2	1	.
6741	2	1	1	1	2	4	2	1	1	1	1	1	3	1	.
749	2	1	1	2	2	3	1	1	1	1	1	1	2	1	.
4701	2	1	3	4	4	4	4	2	1	1	.	1	3	1	.
5250	2	1	2	2	3	4	2	2	1	2	.	1	3	1	.
5314	2	1	2	3	3	3	4	1	1	1	.	1	3	1	.
5510	2	2	1	2	2	3	1	2	1	1	.	1	3	3	.
5790	2	2	1	2	2	1	2	1	1	1	.	1	5	4	.
2157	3	2	3	3	2	4	2	4	2	3	.	1	.	3	.
2409	-1	1	1	1	1	1	2	3	1	1	.	2	3	1	.

5048	3	2	2	3	1	2	2	3	4	2	.	1	4	1	.
3684	2	1	1	3	2	1	3	1	1	1	2	1	2	1	.
5338	2	2	3	3	1	1	1	2	1	1	1	1	4	1	.
1121	2	1	1	4	2	2	3	1	1	2	1	1	.	-1	.
4009	2	1	1	2	1	2	1	1	1	1	1	1	3	1	.
4022	2	2	1	3	2	4	2	1	3	2	2	1	2	3	.
5208	2	4	1	-1	4	-1	4	-1	-1	-1	.	1	.	4	.
5343	2	-1	1	3	3	4	3	4	3	3	.	1	4	3	.
5610	2	2	1	3	4	4	4	2	1	3	2	1	5	4	.
5624	2	2	2	3	3	4	4	4	2	2	1	1	4	4	.
5654	3	2	-1	-1	-1	-1	-1	-1	-1	-1	1	1	4	4	.
4551	3	2	3	3	4	4	4	2	3	4	.	3	5	2	.
5336	2	2	2	3	3	2	4	3	1	3	.	2	4	1	.
6212	3	2	1	3	3	4	3	1	1	1	2	1	.	-1	.
3955	2	2	1	3	3	4	2	1	1	1	1	1	3	1	.
5597	2	2	1	3	3	2	3	2	1	1	1	2	4	1	.
928	1	2	1	1	1	2	2	2	1	2	1	2	4	3	-2
2796	1	4	-1	2	1	4	1	1	4	1	1	1	4	1	-2
6860	1	3	-1	3	3	3	3	3	-1	3	1	1	4	1	-2
2588	1	2	1	4	3	1	1	1	1	1	1	2	5	1	-2
2656	1	1	1	2	2	4	2	2	1	1	.	-1	.	2	-2
3925	1	1	1	3	3	3	2	1	1	1	1	1	4	1	-2
4114	1	3	4	4	2	3	2	2	4	3	.	-1	.	-1	-2
3461	1	1	2	3	2	3	2	1	1	1	1	1	.	-1	-2
3731	1	2	1	2	2	3	2	1	1	1	1	1	4	1	-2
1409	1	1	1	2	2	4	3	2	2	2	.	-1	.	-1	-2
6451	1	2	1	1	1	3	2	-1	1	1	1	1	5	1	-2
2501	1	1	1	1	1	3	2	3	1	1	1	2	1	4	-2
5331	1	2	1	2	2	4	2	1	2	1	.	1	3	4	-2
5596	1	2	4	4	2	4	4	1	1	1	2	1	4	-1	-2
5728	1	2	-1	2	3	3	2	1	1	1	1	1	4	1	-2
3897	1	1	1	1	1	-1	1	1	1	1	.	1	.	1	-2
2117	1	3	2	2	2	3	2	4	3	3	1	2	5	4	-2
4260	1	2	1	-1	3	4	2	1	1	1	2	1	4	2	-2
1462	1	2	1	1	1	4	1	1	1	1	.	2	4	4	-2
2065	1	3	1	3	2	2	2	3	1	4	1	1	2	6	-2

6834	1	3	2	3	2	3	3	2	4	2	1	2	5	3	-2
1676	1	1	1	1	1	1	3	1	1	1	1	2	5	5	-2
2976	1	2	1	4	3	2	2	1	2	2	1	1	1	1	-2
3777	1	1	1	3	3	2	3	2	2	2	.	1	2	1	-2
5178	1	1	3	3	3	1	4	1	2	3	.	2	1	1	-2
3656	1	2	2	2	2	3	2	1	1	1	1	1	4	1	-2
1854	1	2	1	3	2	3	3	2	3	2	.	1	3	1	1
3153	1	1	1	1	1	1	1	1	1	1	.	2	4	4	1
4351	1	2	-1	2	1	3	3	1	1	2	1	1	5	4	1
6327	1	3	1	4	3	4	4	3	2	4	1	1	5	1	1
6405	1	2	-1	3	3	4	4	2	2	2	1	1	4	4	1
1679	1	1	1	1	1	1	2	1	1	1	.	2	4	2	1
2843	1	2	1	2	1	2	2	1	1	1	1	2	4	2	1
3495	1	2	2	2	2	2	2	2	2	2	.	1	4	1	1
3686	1	1	1	3	2	4	3	1	-1	1	1	2	3	1	1
4035	1	2	1	1	1	3	1	1	1	1	1	1	3	1	1
4222	1	1	2	2	2	3	2	2	2	1	.	2	4	1	1
4367	1	2	1	3	2	3	3	2	3	3	.	2	3	3	1
4425	1	2	-1	2	2	3	3	1	1	1	.	1	4	-1	1
5851	1	1	1	1	1	3	1	1	1	1	.	1	5	4	1
6609	1	3	-1	3	2	3	3	3	4	3	1	1	5	8	1
6704	1	1	2	1	2	2	2	-1	1	1	.	-1	1	2	1
2220	1	2	1	3	2	2	2	3	1	2	1	1	4	1	1
2498	1	1	1	3	2	3	3	2	2	2	1	2	4	1	1
2750	1	2	1	3	2	2	1	1	1	1	.	2	2	1	1
2802	1	1	1	2	2	1	2	1	1	1	.	1	4	4	1
3571	1	2	1	4	3	4	3	2	2	2	1	1	4	1	1
3624	1	2	1	4	3	3	3	1	3	3	.	1	4	4	1
3696	1	1	1	3	2	2	2	1	1	1	.	2	3	1	1
3728	1	1	1	1	1	1	1	1	1	1	.	2	3	4	1
3756	1	2	2	3	3	1	3	1	2	1	1	1	3	4	1
3890	1	2	1	2	2	-1	3	2	4	4	.	1	4	1	1
3894	1	1	1	4	1	4	2	1	1	1	1	1	2	1	1
3949	1	1	1	3	1	2	2	1	1	1	1	1	1	1	1
4134	1	2	1	3	1	2	2	1	2	2	2	1	4	2	1
4322	1	2	2	3	3	4	3	2	2	2	.	1	4	8	1

4411	1	2	1	3	2	3	3	2	1	3	.	1	4	3	1
4595	1	2	3	3	2	4	3	2	2	3	.	1	4	5	1
4700	1	1	3	3	1	4	2	1	1	1	1	1	3	1	1
5171	1	2	-1	-1	-1	-1	-1	-1	-1	-1	1	1	5	3	1
5191	1	2	1	2	2	2	2	1	1	1	2	1	4	2	1
5228	1	3	-1	3	3	2	3	1	3	3	1	1	4	4	1
5301	1	2	1	2	2	3	2	1	1	1	2	1	4	1	1
6302	1	2	1	2	2	2	2	2	2	2	1	1	4	1	1
6304	1	1	1	3	2	4	4	1	2	1	.	2	4	1	1
6518	1	3	3	1	1	4	2	2	1	2	.	1	5	1	1
6660	1	2	1	3	2	2	1	1	1	2	1	1	4	1	1
1475	1	1	1	1	1	1	1	1	4	1	1	1	4	2	1
1541	1	2	1	2	2	3	4	1	1	2	1	1	4	4	1
1799	1	2	2	3	3	4	4	1	1	1	1	1	4	1	1
2150	1	3	2	3	3	4	2	3	2	2	.	1	4	4	1
2169	1	3	1	3	2	2	2	3	3	4	2	1	4	1	1
2608	1	1	1	2	2	1	3	1	1	1	.	2	2	1	1
3651	1	3	1	2	2	4	3	1	4	2	2	2	4	4	1
3717	1	1	1	3	3	2	3	1	1	1	.	2	3	1	1
3895	1	1	1	1	1	1	1	1	1	1	1	2	4	1	1
4037	1	2	1	2	1	2	2	1	1	2	1	1	3	1	1
4247	1	1	1	1	1	1	2	1	1	1	1	2	2	1	1
4341	1	2	1	3	1	2	1	2	2	2	1	1	3	1	1
4751	1	2	4	4	4	2	2	1	1	1	.	1	2	1	1
5060	1	1	1	3	2	3	4	2	1	2	.	1	3	4	1
5136	1	2	1	3	3	4	3	2	1	2	1	1	3	1	1
5815	1	2	1	2	3	3	3	1	1	1	2	1	2	4	1
6449	1	1	1	2	2	3	3	1	2	2	.	2	4	3	1
6551	1	3	2	2	3	1	3	2	2	3	2	1	5	1	1
6685	1	1	1	2	1	1	3	1	3	3	1	1	1	4	1
801	1	1	1	1	2	3	2	2	1	1	2	1	4	4	1
802	1	2	1	2	2	3	2	2	1	2	.	1	2	1	1
812	1	2	2	3	3	2	4	2	2	2	1	2	5	1	1
1525	1	2	1	2	1	2	3	1	1	1	.	2	4	1	1
1598	1	2	1	2	2	2	3	1	2	1	1	1	4	4	1
2044	1	2	1	3	2	3	2	1	3	2	.	1	4	1	1

2178	1	2	1	1	1	3	3	1	3	1	.	1	5	1	1
2219	1	1	1	2	2	2	3	1	1	1	1	1	4	3	1
3802	1	1	1	4	4	3	3	2	2	2	.	1	3	1	1
4208	1	1	1	3	4	3	2	2	1	1	.	1	1	1	1
4257	1	2	1	1	1	2	1	1	1	1	1	1	.	1	1
4308	1	2	1	2	1	1	1	1	1	1	.	1	4	1	1
4413	1	2	-1	3	2	1	2	3	1	2	2	1	1	1	1
4521	1	2	1	3	2	3	2	1	1	1	1	1	4	-1	1
4616	1	3	3	4	4	4	4	1	3	3	2	1	.	4	1
4711	1	1	1	2	2	1	3	1	1	1	2	1	4	4	1
5034	1	2	2	2	2	3	3	1	1	3	2	2	5	1	1
6271	1	1	1	2	2	2	2	1	2	2	.	2	4	1	1
6394	1	2	2	2	2	3	3	1	2	1	.	1	3	1	1
6821	1	2	2	4	1	1	1	1	1	1	.	2	4	1	1
1647	1	2	1	2	2	3	3	1	4	2	2	2	5	1	1
1750	1	1	2	3	3	1	3	1	1	1	.	1	2	1	1
2590	1	3	2	3	1	3	1	1	2	3	2	1	4	2	1
2729	1	2	1	3	3	4	2	2	2	2	2	1	2	1	1
3622	1	1	1	1	1	4	2	1	1	1	.	2	5	1	1
3840	1	1	2	1	1	3	2	1	1	1	.	1	4	2	1
4097	1	2	1	1	1	3	2	1	1	1	1	2	5	1	1
5334	1	1	1	3	2	1	2	1	1	1	.	2	1	1	1
6760	1	2	1	3	3	3	4	1	1	1	1	2	1	4	1
1085	1	2	1	3	3	2	3	1	1	2	2	1	4	4	1
1456	1	2	1	3	3	3	3	2	2	1	.	1	4	3	1
3936	1	1	3	3	3	4	3	1	1	1	.	2	4	1	1
4423	1	2	1	4	4	2	3	1	1	1	1	2	4	2	1
4562	1	2	3	3	3	4	3	1	1	2	.	-1	.	1	1
4621	1	2	2	3	3	3	3	1	2	2	2	1	5	4	1
4909	1	2	3	3	2	2	2	1	1	1	.	1	.	2	1
5104	1	2	4	2	2	1	3	1	1	2	2	1	5	4	1
5537	1	2	1	2	2	2	3	2	2	2	1	1	5	1	1
6156	1	1	3	3	3	2	2	2	1	1	.	1	2	1	1
6182	1	2	1	2	1	3	2	1	1	1	.	2	2	1	1
6539	1	2	3	3	3	4	2	3	1	2	.	1	4	1	1
2112	1	1	1	2	1	3	4	1	1	1	1	2	5	1	1

2385	1	1	1	1	1	3	2	1	1	1	.	1	4	2	1
5458	1	1	1	1	1	1	2	2	1	1	.	1	1	2	1
3343	1	1	1	3	1	4	2	4	2	1	1	1	2	5	1
2793	1	1	1	3	1	3	1	1	1	1	.	2	2	1	1
2462	1	1	1	1	1	1	3	1	1	1	.	1	2	5	1
786	1	1	1	2	2	2	1	1	1	1	.	1	3	1	1
849	1	1	1	3	2	3	4	3	1	1	.	1	3	4	1
1490	1	-1	1	3	3	3	3	1	4	4	1	1	4	4	1
2182	1	2	1	2	2	2	2	2	1	2	1	1	4	2	1
2735	1	2	1	3	2	1	2	1	2	1	.	1	3	1	1
3617	1	2	1	2	1	3	3	2	2	2	1	1	4	4	1
3877	1	1	1	3	3	1	3	2	1	1	1	1	5	1	1
3986	1	2	2	2	3	3	3	2	2	3	2	1	5	5	1
5557	1	1	1	2	2	3	3	2	1	1	1	1	4	3	1
5678	1	2	-1	-1	-1	-1	-1	-1	-1	-1	.	2	4	4	1
6740	1	2	1	3	3	4	4	2	1	2	.	1	5	4	1
6754	1	3	3	3	-9	-9	3	-9	-9	-9	2	2	5	1	1
1226	1	3	1	3	3	2	3	1	3	2	2	2	4	4	1
4444	1	1	1	1	1	4	3	1	1	1	.	1	.	2	1
6254	1	2	1	1	1	1	1	1	1	1	1	2	5	3	1
6544	1	3	-1	4	3	-1	-1	-1	-1	3	2	1	5	4	1
2145	1	2	1	2	1	4	4	1	1	1	2	1	4	4	2
5267	1	1	1	1	1	3	2	1	1	1	1	2	2	1	2
5283	1	1	1	1	1	3	1	1	1	1	.	2	2	4	2
5304	1	1	1	2	2	2	3	2	2	1	1	2	2	1	2
5505	1	2	1	1	1	1	1	1	1	1	.	2	3	1	2
5514	1	1	1	2	2	2	1	1	1	1	.	1	2	1	2
6552	1	2	1	1	1	1	1	1	1	1	2	1	5	1	2
819	1	2	1	2	2	3	3	2	2	2	2	1	4	4	2
965	1	2	-1	2	2	-1	3	3	3	3	.	1	.	5	2
1430	1	2	4	2	2	2	2	3	3	2	1	2	4	2	2
1666	1	2	1	3	3	2	4	3	3	2	2	2	4	1	2
2092	1	2	1	3	3	4	1	4	1	3	2	1	2	4	2
2098	1	1	1	2	1	1	1	1	1	1	1	2	4	-1	2
2389	1	1	1	3	2	4	3	1	1	1	1	2	5	3	2
2581	1	2	1	2	2	3	3	2	2	2	1	2	4	5	2

3964	1	1	1	1	1	2	2	1	1	1	1	1	2	1	2
4047	1	3	2	3	3	2	3	2	3	3	.	2	5	4	2
4228	1	2	1	2	1	3	2	1	1	2	1	1	3	1	2
4704	1	2	1	2	1	1	3	1	1	1	.	2	4	1	2
5626	1	2	-1	3	3	-1	3	2	3	3	2	2	5	1	2
6199	1	1	3	1	1	4	2	3	2	2	2	1	5	1	2
6554	1	2	2	3	3	2	2	2	2	2	1	1	4	3	2
6576	1	2	4	3	3	3	4	1	1	3	2	1	4	2	2
6580	1	3	1	3	3	4	2	1	2	3	2	1	5	4	2
6727	1	2	1	2	3	4	2	3	1	2	1	1	3	3	2
805	1	2	1	2	2	2	2	2	2	2	2	1	4	1	2
883	1	2	2	1	1	4	1	1	1	1	1	1	.	1	2
1458	1	4	2	4	4	4	4	-1	3	4	1	1	4	5	2
1574	1	1	1	3	2	2	2	-1	1	1	1	1	2	3	2
1577	1	3	1	3	1	3	3	3	1	3	.	1	4	4	2
1719	1	2	2	2	3	2	3	1	1	1	2	2	5	8	2
2094	1	2	1	3	3	3	4	1	1	3	1	1	3	4	2
2256	1	2	1	1	1	1	1	1	3	1	2	1	4	1	2
2518	1	2	1	3	3	4	3	3	1	2	.	1	1	2	2
2704	1	1	1	1	1	1	1	1	1	1	1	2	3	2	2
2810	1	2	1	2	2	2	2	1	-1	-1	.	2	2	3	2
3219	1	2	1	1	1	1	1	1	1	1	.	1	5	1	2
3489	1	1	-1	2	2	1	1	-1	3	4	.	2	5	1	2
3788	1	3	1	3	3	2	3	1	1	4	2	1	4	4	2
3790	1	2	2	2	2	3	3	3	1	1	2	1	4	2	2
3896	1	3	3	3	3	1	4	1	3	4	.	1	5	3	2
4107	1	2	1	2	2	3	2	3	1	1	2	1	5	2	2
4244	1	2	1	1	1	2	1	2	1	1	1	1	.	1	2
4683	1	2	3	3	1	4	4	4	3	1	1	1	4	1	2
4845	1	2	1	2	1	1	2	1	1	2	2	1	4	4	2
4903	1	2	1	3	2	4	3	2	1	2	2	2	4	4	2
5135	1	2	1	2	2	4	3	2	3	1	.	2	4	4	2
5315	1	2	1	1	1	3	1	1	2	2	1	1	3	4	2
5320	1	1	1	1	1	1	1	1	1	1	2	1	2	4	2
5357	1	2	2	2	1	3	1	2	2	2	2	1	1	2	2
6275	1	2	2	3	3	3	4	1	1	3	1	1	4	4	2

6443	1	2	2	-1	2	3	3	2	1	1	1	2	4	1	2
6505	1	2	1	1	1	1	1	1	1	1	2	1	4	1	2
6797	1	1	1	2	2	4	2	1	1	1	.	2	3	4	2
6939	1	3	1	3	2	3	2	2	2	2	2	2	5	-1	2
1233	1	3	3	3	2	2	2	2	1	3	.	1	5	1	2
1440	1	2	2	3	2	2	3	2	3	2	.	1	5	4	2
1579	1	1	1	2	2	3	1	1	1	1	.	1	1	3	2
1705	1	1	1	2	2	1	2	1	1	1	1	2	5	1	2
2602	1	1	1	3	3	2	2	1	1	1	.	2	1	4	2
2719	1	3	1	3	3	3	4	3	3	4	2	1	3	4	2
3247	1	1	1	2	2	1	3	1	1	1	.	1	1	4	2
3303	1	2	2	3	3	2	4	2	2	2	.	1	3	1	2
3410	1	3	2	2	2	2	2	2	2	2	2	1	5	3	2
3600	1	1	1	2	2	4	2	1	1	1	1	2	4	3	2
3626	1	1	1	2	2	4	2	1	1	1	1	2	3	1	2
3681	1	1	1	1	1	1	1	1	1	1	2	1	4	1	2
3888	1	3	2	3	2	1	3	-1	2	4	.	1	5	4	2
3989	1	1	1	3	3	1	3	2	1	1	.	2	1	2	2
4242	1	1	1	2	2	2	2	1	1	1	1	1	4	5	2
4461	1	2	-1	3	3	2	3	2	2	2	1	1	4	4	2
4790	1	2	3	3	3	2	2	1	3	2	2	1	4	2	2
4924	1	2	1	3	3	3	3	3	3	2	.	1	4	8	2
5174	1	2	2	2	2	2	2	1	2	2	1	1	4	2	2
5303	1	2	1	2	1	4	1	2	1	2	.	1	3	1	2
5735	1	2	1	2	2	2	2	1	2	2	.	1	4	1	2
5880	1	2	1	2	1	4	2	1	2	1	.	1	3	1	2
6204	1	2	1	3	2	2	2	2	1	1	1	2	5	1	2
6276	1	2	1	2	1	2	1	1	2	1	2	2	5	1	2
6611	1	1	1	2	2	3	3	2	1	1	1	1	5	4	2
6702	1	2	1	3	2	2	2	1	1	1	.	1	4	2	2
6728	1	2	1	2	2	1	3	1	1	1	.	2	4	3	2
6758	1	2	2	2	3	3	2	2	2	1	.	2	5	1	2
6842	1	2	1	2	2	3	3	1	2	1	2	1	5	1	2
1434	1	1	3	3	3	4	4	1	1	1	.	2	2	4	2
1602	1	2	1	2	2	3	2	2	2	1	2	1	3	1	2
2504	1	2	1	1	1	1	2	1	1	1	2	1	4	3	2

2606	1	1	2	2	1	3	2	1	1	2	.	1	4	2	2
2607	1	1	1	1	1	1	1	1	1	1	1	2	4	1	2
2812	1	2	2	3	2	4	3	1	1	1	1	2	5	1	2
3463	1	2	2	3	3	1	1	1	2	2	1	1	4	3	2
3490	1	2	1	2	1	3	1	1	2	2	.	1	2	1	2
3593	1	3	1	3	2	2	4	2	2	2	2	1	4	3	2
4048	1	2	1	2	2	4	2	1	1	2	1	1	5	1	2
4149	1	1	2	2	1	4	3	2	4	3	1	2	3	4	2
4362	1	1	1	1	1	1	1	1	1	1	1	1	5	1	2
4447	1	2	1	2	2	3	2	1	1	1	1	1	4	4	2
4538	1	3	1	3	2	1	2	1	1	3	.	2	5	3	2
4539	1	2	2	2	2	3	2	2	2	2	.	1	5	1	2
4766	1	1	3	4	3	2	4	2	1	1	2	1	3	5	2
4953	1	2	1	2	1	2	2	1	1	2	.	1	4	8	2
5156	1	2	3	2	2	3	3	1	2	2	.	1	5	4	2
5558	1	2	2	2	2	3	3	2	2	2	.	1	4	4	2
5565	1	1	1	1	2	3	1	1	1	1	.	1	1	2	2
6207	1	1	2	1	2	2	2	1	1	1	.	1	5	2	2
1506	1	1	1	3	1	1	2	1	1	1	1	1	4	4	2
1669	1	2	2	2	2	1	4	1	2	2	2	2	5	5	2
2387	1	1	1	2	2	1	2	1	1	1	1	2	4	1	2
3019	1	2	2	3	3	3	3	2	1	1	.	1	5	1	2
3907	1	2	2	4	3	2	4	2	4	3	.	1	4	4	2
4042	1	2	1	2	2	3	4	2	1	2	1	1	4	4	2
4210	1	2	2	2	2	3	4	2	2	2	.	1	4	4	2
4262	1	1	1	2	2	4	2	1	1	1	.	2	4	1	2
4563	1	1	1	2	2	4	2	1	1	1	.	1	3	1	2
4608	1	2	4	3	3	2	2	-9	-9	-9	.	2	4	4	2
4636	1	1	3	3	2	4	4	2	1	1	.	1	4	1	2
4681	1	2	2	2	2	2	3	2	1	1	.	1	5	4	2
4712	1	1	3	3	1	3	1	2	1	1	1	1	2	1	2
4869	1	1	2	2	1	1	1	1	1	1	1	1	5	4	2
4893	1	2	1	2	1	3	3	1	1	1	.	1	4	1	2
5063	1	1	1	1	1	1	2	1	1	1	.	2	3	1	2
5249	1	1	1	2	3	4	3	1	2	2	.	2	4	2	2
5278	1	1	1	1	1	2	1	1	1	1	1	1	4	1	2

5575	1	1	1	1	1	1	2	1	1	1	.	1	2	2	2
6278	1	1	1	1	1	1	2	1	1	1	1	2	4	2	2
6342	1	1	1	3	2	2	1	1	1	1	.	1	2	1	2
6403	1	1	1	2	1	2	2	2	1	1	.	1	3	1	2
6832	1	2	1	2	2	3	2	3	2	2	.	1	3	1	2
1502	1	2	1	4	2	4	2	2	1	2	.	1	4	1	2
3864	1	3	1	3	3	1	1	1	3	3	.	1	3	4	2
3988	1	1	2	3	3	2	3	2	2	2	1	1	3	4	2
4486	1	1	1	1	1	3	3	1	1	1	.	1	4	1	2
5142	1	2	2	3	2	2	1	1	1	2	2	1	4	3	2
5734	1	1	1	2	1	1	1	1	1	1	1	1	2	3	2
6269	1	2	1	3	2	2	1	1	1	2	.	1	4	2	2
6307	1	1	2	3	2	3	2	2	1	1	.	1	2	1	2
6489	1	1	1	2	2	2	4	1	1	1	.	1	4	4	2
1178	1	2	1	2	3	2	2	2	2	2	2	1	5	-1	2
2515	1	1	1	2	3	2	3	1	1	1	.	1	1	2	2
4053	1	1	1	2	2	2	3	3	1	3	2	1	3	4	2
4131	1	1	1	2	1	2	1	4	1	1	1	1	4	1	2
4453	1	1	2	2	2	2	2	2	2	2	.	1	4	1	2
4770	1	2	1	2	2	4	4	1	1	1	2	1	5	1	2
4852	1	1	2	2	1	2	1	1	1	1	.	2	2	1	2
5011	1	2	3	3	3	3	2	3	2	2	.	1	4	4	2
5054	1	2	2	4	4	4	3	3	2	2	.	1	4	1	2
1275	1	1	2	2	3	1	1	2	1	1	.	2	4	1	2
2146	1	2	1	2	2	2	3	1	1	2	.	2	5	2	2
2726	1	1	1	1	1	2	1	1	1	1	.	2	4	1	2
3620	1	3	2	3	3	3	3	3	1	3	.	1	5	2	2
3959	1	2	1	2	3	3	2	1	1	2	.	1	1	4	2
4118	1	1	1	4	3	2	4	3	3	2	.	1	4	1	2
4806	1	2	3	4	3	2	3	1	1	3	.	1	1	3	2
5482	1	2	1	2	1	3	3	1	3	2	2	1	5	2	2
6534	1	1	1	2	2	3	2	1	1	1	.	1	4	2	2
1554	1	1	1	3	3	3	3	1	1	1	.	1	.	1	2
2573	1	1	2	2	1	2	2	1	1	1	.	1	.	1	2
4279	1	1	3	1	1	1	1	1	1	1	.	2	4	1	2
6711	1	2	2	2	2	3	2	2	2	2	.	2	4	1	2

6378	1	2	1	1	1	2	3	2	1	1	.	2	4	1	2
4843	1	-1	1	1	1	1	1	1	1	1	.	1	.	-1	2
5173	1	3	1	1	2	1	2	2	1	3	1	2	5	1	2
2131	1	3	3	3	3	1	3	2	2	3	.	1	4	8	2
3900	1	2	1	3	3	3	3	2	2	2	1	1	4	4	2
3912	1	2	2	3	2	3	3	2	2	3	2	2	4	1	2
5036	1	3	1	3	3	2	2	2	2	3	2	1	5	3	2
6347	1	1	1	2	1	2	1	1	1	1	.	1	4	4	2
6427	1	2	2	2	3	3	3	3	2	2	1	1	4	5	2
1031	1	2	3	3	2	2	1	2	2	3	.	1	4	4	2
1222	1	2	1	1	1	2	1	1	1	1	.	1	5	1	2
3917	1	1	1	1	1	1	1	1	1	1	1	1	5	1	2
4702	1	1	4	3	2	4	4	1	1	1	1	2	4	5	2
5346	1	1	1	2	3	1	-1	1	1	1	.	2	5	1	2
6532	1	3	3	3	2	1	2	2	1	3	2	1	5	4	2
1217	1	2	1	2	3	3	3	2	1	1	2	1	5	5	2
2964	1	2	2	3	3	3	3	2	2	2	1	1	3	3	2
4158	1	2	1	2	3	2	2	2	1	1	1	1	4	4	2
6074	1	4	2	3	3	4	4	3	4	3	2	-1	.	1	2
6217	1	3	4	3	4	1	2	1	1	3	2	1	5	5	2
6783	1	3	3	4	4	3	3	2	2	3	.	1	5	4	2
1431	1	2	1	2	2	2	1	1	2	2	.	1	4	4	2
1707	1	2	1	1	1	1	1	1	1	1	.	2	5	3	2
1992	1	3	1	3	3	1	3	1	1	2	2	1	4	3	2
3328	1	1	1	3	2	4	2	2	1	1	.	1	1	1	2
4241	1	3	-1	3	3	2	2	-1	4	3	2	1	5	4	2
4379	1	2	1	1	1	2	2	1	2	1	2	1	4	4	2
4637	1	2	4	3	3	3	4	2	2	2	2	1	2	4	2
4707	1	2	2	2	2	3	3	3	3	2	2	1	5	1	2
4860	1	3	2	2	1	2	2	-1	-1	2	2	1	5	3	2
5340	1	2	1	3	2	2	3	1	1	1	.	1	3	5	2
5363	1	1	1	2	1	4	2	1	4	1	.	2	4	1	2
6419	1	2	2	3	2	2	3	1	1	1	.	2	3	1	2
6420	1	2	1	3	3	3	2	1	1	1	1	1	3	2	2
6429	1	2	1	1	1	1	1	1	1	1	.	1	4	3	2
6647	1	1	1	2	1	4	2	1	1	1	2	2	.	2	2

6885	1	3	1	2	2	3	2	1	1	1	2	1	5	1	2
1627	1	2	1	1	1	3	1	1	1	1	.	2	5	1	2
2138	1	2	1	3	2	1	1	1	1	1	.	1	4	2	2
2139	1	2	2	2	2	2	3	3	2	3	1	1	5	5	2
2664	1	1	2	2	2	2	2	2	2	2	1	1	3	1	2
2754	1	1	1	2	2	2	2	2	1	2	1	1	2	1	2
3321	1	2	1	2	3	1	4	4	1	4	.	1	4	4	2
3811	1	1	1	2	2	2	1	1	1	2	2	1	1	1	2
4226	1	2	1	3	3	4	2	1	1	1	.	1	4	4	2
4232	1	2	1	1	1	2	2	1	3	3	2	2	5	1	2
4881	1	3	4	4	4	1	2	1	4	4	1	1	5	2	2
5201	1	1	1	2	2	2	2	1	1	1	.	1	2	2	2
5371	1	2	1	3	3	3	3	2	2	2	1	2	2	1	2
5743	1	1	1	3	2	3	4	4	1	1	.	1	3	2	2
6093	1	2	1	3	2	4	2	3	2	2	2	1	2	5	2
6334	1	2	3	3	3	2	3	2	1	3	2	1	3	1	2
6815	1	3	1	3	1	3	2	2	3	3	2	1	4	8	2
1621	1	2	1	3	2	2	1	1	1	1	.	1	4	2	2
2030	1	2	1	2	1	1	1	1	1	3	.	1	3	1	2
3576	1	1	1	1	1	1	2	1	1	1	.	2	3	3	2
3657	1	2	1	3	2	3	3	2	2	2	1	1	3	-1	2
4041	1	2	1	3	3	1	2	1	1	1	.	1	4	1	2
4346	1	2	1	2	3	2	3	2	2	2	2	1	4	4	2
4693	1	3	2	3	3	2	2	-1	3	3	2	1	4	4	2
5306	1	3	1	2	2	4	2	2	3	2	.	3	2	1	2
5889	1	2	2	3	2	2	4	2	2	2	2	1	3	1	2
6318	1	1	1	1	1	1	2	1	1	1	2	2	5	5	2
6369	1	3	2	3	2	4	3	2	-1	3	1	2	5	1	2
6497	1	2	1	3	3	1	3	3	1	2	.	1	4	3	2
6606	1	1	3	2	2	3	2	2	1	1	.	-1	.	1	2
6736	1	1	1	1	2	3	2	3	1	1	.	2	3	5	2
880	1	2	1	2	3	4	3	1	1	2	.	1	4	1	2
1738	1	2	1	2	2	1	1	3	3	3	2	2	4	1	2
2493	1	2	3	3	2	3	2	2	2	2	.	1	5	3	2
3697	1	2	1	2	2	2	2	1	1	1	.	1	4	1	2
3903	1	2	3	3	2	3	2	2	4	1	.	1	4	5	2

4040	1	2	4	4	3	3	4	1	4	1	2	1	4	4	2
4098	1	1	1	3	2	4	3	2	1	1	2	1	4	1	2
4120	1	2	1	1	1	2	3	2	2	2	2	1	5	4	2
4339	1	2	1	2	3	3	4	2	1	1	1	1	4	4	2
4412	1	2	2	3	3	4	4	4	1	3	2	1	4	4	2
4549	1	1	2	2	2	2	3	1	2	1	1	2	4	4	2
5715	1	1	1	1	1	3	2	1	1	1	.	1	3	4	2
5821	1	2	2	2	3	2	3	1	1	1	1	2	4	1	2
6859	1	2	1	2	2	2	1	1	1	1	2	1	4	1	2
2508	1	2	1	2	2	4	3	1	2	2	2	1	4	5	2
2877	1	1	3	2	2	4	2	3	3	2	.	2	4	2	2
3596	1	2	1	3	3	1	3	2	1	2	.	1	4	1	2
3863	1	2	1	2	2	4	2	2	1	1	2	2	5	1	2
3997	1	2	1	4	3	1	2	1	1	1	.	2	3	1	2
4073	1	2	1	1	2	2	2	3	1	2	2	1	5	1	2
5573	1	1	1	2	4	3	3	2	1	1	1	2	2	2	2
5757	1	1	1	3	1	2	3	3	1	1	.	1	3	3	2
6626	1	2	1	2	2	2	2	1	1	2	2	1	.	2	2
6679	1	2	1	2	1	1	2	1	1	1	1	2	4	1	2
2101	1	2	2	4	3	2	3	1	4	2	.	1	4	4	2
3607	1	1	1	3	2	3	2	2	1	1	.	2	2	1	2
3646	1	2	3	3	3	3	3	3	3	3	.	1	3	1	2
4109	1	2	1	3	2	2	2	2	2	3	2	1	3	1	2
4189	1	2	1	1	2	1	1	1	1	1	.	2	4	1	2
5571	1	3	-1	3	3	3	4	2	3	3	.	1	.	-1	2
6514	1	1	2	3	4	4	3	4	1	2	.	1	4	2	2
1429	1	2	2	2	2	4	1	2	1	1	.	1	5	1	2
2727	1	1	1	1	1	3	2	1	1	1	.	1	3	2	2
2736	1	2	2	2	2	3	3	2	2	2	.	1	4	4	2
5661	1	1	1	1	1	1	1	1	1	1	.	1	2	3	2
6103	1	1	1	2	2	3	3	3	1	1	.	1	4	4	2
4427	1	2	1	2	3	1	3	1	1	1	.	1	2	3	2
4544	1	1	1	3	3	2	3	1	1	1	1	1	3	2	2
5820	1	1	3	1	1	1	3	-1	1	1	.	1	5	1	2
6285	1	1	1	1	1	1	1	1	1	1	2	2	5	1	2
5019	1	1	1	1	1	1	1	1	1	1	2	1	3	3	2

3729	1	-1	3	4	3	3	3	2	1	1	1	1	4	4	2
2661	1	3	1	4	2	3	3	3	2	3	1	1	4	3	2
4108	1	1	1	1	1	4	1	1	1	1	1	2	4	4	2
6029	1	1	1	2	2	4	3	1	1	1	.	2	4	4	2
6335	1	2	1	3	1	2	2	2	1	1	2	1	5	2	2
1594	1	3	2	3	2	3	3	3	3	3	2	1	4	2	2
1691	1	3	4	3	3	3	3	2	3	3	2	2	5	5	2
1593	1	2	1	1	1	2	2	1	1	1	1	1	4	2	3
4081	1	2	1	2	2	3	3	3	1	1	.	1	3	3	3
4387	1	3	1	4	4	3	4	2	2	4	2	1	4	3	3
5689	1	2	1	2	2	3	3	3	2	2	.	2	4	5	3
6272	1	2	1	1	2	2	1	1	1	1	1	1	4	1	3
6292	1	3	2	3	3	3	4	3	2	2	1	1	4	4	3
6358	1	2	1	3	4	3	4	3	2	3	1	1	4	4	3
6771	1	3	2	3	2	3	3	1	2	2	.	2	.	1	3
818	1	1	1	1	1	4	2	1	1	1	.	1	4	1	3
2396	1	3	2	4	4	3	4	2	3	3	.	2	4	-1	3
2472	1	1	1	1	1	2	1	2	1	1	.	1	.	2	3
2737	1	2	1	3	2	2	2	1	1	1	.	1	1	4	3
3355	1	2	1	2	2	3	3	1	1	1	.	2	5	3	3
4086	1	2	1	2	2	2	2	3	2	2	.	2	3	3	3
4392	1	2	2	2	3	3	2	2	2	2	1	1	3	1	3
5133	1	1	1	4	1	1	2	4	1	1	.	1	3	1	3
5618	1	3	1	2	3	3	3	1	2	2	2	1	4	3	3
6215	1	2	1	3	2	2	3	3	1	1	2	1	1	2	3
6477	1	2	3	4	4	3	2	2	2	3	.	1	2	4	3
6703	1	2	1	2	2	2	3	2	2	3	1	1	4	4	3
822	1	1	1	3	1	4	1	1	1	1	.	1	4	1	3
1553	1	2	1	4	2	1	2	1	1	1	.	1	4	2	3
1721	1	2	1	3	2	2	3	1	1	1	2	1	3	4	3
2432	1	2	2	3	3	4	3	2	3	3	2	2	2	2	3
2690	1	1	1	1	1	1	1	1	1	1	.	1	2	1	3
2695	1	2	1	2	2	3	1	1	1	1	.	1	2	1	3
3137	1	2	1	3	3	2	4	1	1	1	2	1	4	4	3
3428	1	3	1	1	3	3	3	4	3	3	2	1	4	8	3
3570	1	2	1	3	1	2	1	1	1	1	1	1	4	4	3

4036	1	2	1	2	3	1	3	1	1	1	1	1	4	2	3
4235	1	2	1	3	3	2	4	1	3	2	.	1	4	3	3
4811	1	2	2	3	3	3	3	2	2	2	.	1	4	2	3
6175	1	2	2	4	4	4	3	2	3	3	.	1	3	1	3
997	1	4	2	4	4	2	3	2	4	4	2	2	4	5	3
1605	1	2	1	4	3	1	1	1	1	1	1	1	3	1	3
1619	1	1	1	2	2	2	3	1	2	1	2	1	4	4	3
1781	1	1	3	1	1	-1	1	3	1	1	2	1	.	2	3
2134	1	1	1	1	1	1	2	1	1	1	.	2	5	1	3
2349	1	2	1	1	2	1	3	1	1	1	2	2	5	4	3
2807	1	1	1	3	3	3	4	1	1	1	1	2	3	4	3
2854	1	2	1	1	1	3	3	1	2	2	.	1	2	3	3
3174	1	2	1	2	2	1	1	1	1	1	.	1	3	1	3
3578	1	2	1	3	3	4	3	2	2	2	2	1	4	1	3
3871	1	2	2	2	1	2	3	2	2	2	1	1	5	5	3
4011	1	1	1	1	2	4	2	1	1	1	.	2	4	1	3
4477	1	2	1	4	3	2	2	3	2	2	1	1	2	4	3
4479	1	2	1	3	2	4	2	3	1	1	.	2	5	1	3
5213	1	2	-1	3	2	1	3	1	2	1	.	2	5	4	3
6408	1	2	1	2	2	2	3	1	1	1	.	2	4	5	3
1985	1	1	2	3	2	1	2	1	1	1	.	2	3	1	3
2235	1	2	1	3	2	2	2	2	2	2	1	2	5	1	3
2437	1	1	1	1	1	1	1	3	1	1	1	1	1	1	3
3619	1	2	3	3	2	4	2	2	2	2	.	2	4	1	3
3631	1	2	1	2	3	3	2	1	2	2	1	1	4	4	3
4347	1	1	-1	3	3	2	2	2	2	1	.	2	3	1	3
4876	1	2	4	4	4	3	3	2	2	3	2	1	4	2	3
5266	1	2	1	3	2	2	4	3	1	1	1	2	3	2	3
5681	1	1	1	1	1	2	2	1	1	1	.	1	4	3	3
5940	1	2	1	3	3	4	3	1	1	1	.	1	4	4	3
6150	1	3	2	4	2	3	2	3	2	3	2	1	5	1	3
6316	1	1	1	1	1	3	1	1	1	1	.	2	3	1	3
6639	1	1	1	2	2	3	1	1	1	1	.	1	4	1	3
3088	1	3	1	4	4	4	4	1	3	4	2	1	4	5	3
3445	1	1	1	1	1	2	2	1	1	1	1	2	5	1	3
3865	1	1	1	2	1	2	1	1	1	1	1	1	3	1	3

3882	1	2	1	1	1	1	2	1	1	1	.	1	4	4	3
4083	1	1	1	2	1	1	1	1	1	1	.	1	4	2	3
4135	1	2	3	3	1	2	2	1	1	1	2	1	4	4	3
4205	1	2	2	2	3	3	3	2	2	2	.	1	2	1	3
4398	1	1	1	2	2	3	2	1	1	1	1	1	2	2	3
5046	1	1	1	1	1	3	1	2	1	1	2	1	5	1	3
6303	1	2	2	3	3	3	1	1	1	1	.	1	5	1	3
6470	1	2	1	4	4	3	4	1	3	3	1	1	4	4	3
2347	1	1	1	1	1	4	1	3	1	1	.	2	4	2	3
2614	1	1	1	1	2	3	2	1	1	1	.	2	3	1	3
3138	1	2	1	3	1	2	1	3	2	3	.	1	4	1	3
4428	1	2	1	3	3	2	2	3	2	2	.	2	4	4	3
6048	1	1	2	2	2	4	4	4	2	2	.	1	3	8	3
6122	1	2	3	3	1	3	3	4	1	1	.	2	2	4	3
4055	1	2	1	3	3	3	3	2	2	2	.	1	4	4	3
4141	1	2	2	2	2	2	3	1	1	1	.	1	5	1	3
5244	1	3	2	3	2	3	3	2	2	2	.	2	.	4	3
6270	1	2	-1	2	1	-1	2	1	-1	2	1	2	5	1	3
6556	1	2	2	2	2	3	2	3	1	1	1	1	4	2	3
1452	1	2	1	2	2	2	4	2	1	1	.	1	4	4	3
1463	1	2	2	4	3	2	4	2	4	2	1	1	5	4	3
2586	1	2	1	3	4	3	3	4	1	2	.	1	1	5	3
5339	1	2	1	2	3	3	3	2	2	2	.	1	2	1	3
5493	1	2	-1	3	3	2	4	2	1	3	.	1	3	7	3
3023	1	2	1	3	3	2	4	3	2	3	.	1	4	4	3
4008	1	2	1	1	1	1	2	1	1	1	.	1	2	1	3
4095	1	2	1	3	1	3	2	1	1	2	2	1	4	4	3
5130	1	2	1	3	3	3	3	1	1	1	.	1	4	1	3
5994	1	3	1	4	1	4	3	1	1	1	.	-1	4	8	3
6654	1	1	2	3	2	3	4	2	2	4	2	1	5	5	3
1689	1	2	-1	3	-1	3	3	1	1	1	1	2	5	2	3
2526	1	2	1	2	2	4	2	2	2	2	2	-1	.	1	3
3737	1	2	-1	3	2	-1	2	1	1	1	2	2	5	1	3
4391	1	2	2	3	2	4	4	1	3	1	.	-1	4	1	3
5941	1	2	1	2	-1	1	2	1	1	1	.	2	4	1	3
6343	1	1	1	1	1	1	1	1	1	1	1	2	3	1	3

1459	1	2	2	3	2	2	3	1	1	1	.	1	5	5	3
1678	1	2	2	2	1	3	3	1	1	1	.	2	5	5	3
1714	1	1	1	3	1	2	2	1	1	1	.	1	4	-1	3
2560	1	2	3	3	3	2	3	3	2	2	1	1	3	4	3
2594	1	2	1	2	2	1	3	2	1	2	1	1	4	4	3
4045	1	2	1	2	2	2	3	1	1	1	.	1	4	4	3
4174	1	1	1	2	1	2	1	2	1	1	.	1	4	8	3
4804	1	2	3	3	3	2	3	1	2	3	.	1	4	1	3
1993	1	1	3	3	1	1	2	1	2	1	1	2	4	1	3
2832	1	1	1	4	3	2	1	1	1	1	.	2	2	1	3
3984	1	1	1	2	2	3	2	1	1	1	1	1	3	4	3
4443	1	2	3	3	3	2	3	2	2	2	1	1	5	3	3
3581	1	1	1	2	2	3	2	1	1	2	1	1	1	4	3
3613	1	2	2	3	2	3	2	1	1	1	.	1	3	1	3
3623	1	2	1	4	2	4	2	1	1	1	.	1	5	3	3
4481	1	2	2	3	1	4	2	2	1	2	1	1	5	1	3
4785	1	2	2	2	1	4	2	1	1	1	.	1	4	3	3
5064	1	2	1	2	2	3	3	1	1	1	.	2	4	5	3
6884	1	2	2	3	3	4	3	2	2	2	.	1	4	1	3
1556	1	2	1	2	1	3	3	1	3	4	.	1	5	5	3
4204	1	1	1	2	2	3	1	3	1	1	1	2	4	1	3
5951	1	2	1	2	2	3	3	1	1	1	.	1	3	1	3
3629	1	1	1	3	3	4	4	1	1	1	.	2	4	3	3
2222	1	1	-1	4	4	3	4	2	-1	-1	.	2	2	4	3
4062	1	3	2	4	4	2	4	2	2	4	2	1	5	5	3
5984	1	2	2	2	2	2	2	2	2	2	2	2	5	3	3
4802	1	3	3	4	2	4	2	2	3	4	2	1	5	1	3
5639	1	3	2	3	3	3	3	2	2	2	.	1	.	4	3
1467	1	3	2	3	3	3	3	2	3	3	.	2	4	4	4
5724	1	2	1	3	1	3	1	1	1	1	1	2	.	1	4
4424	1	2	1	2	2	3	2	1	3	3	.	1	5	1	4
5737	1	1	2	2	3	4	2	1	1	1	.	1	4	3	4
6296	1	2	2	2	2	3	2	1	1	2	1	2	3	2	4
1734	1	1	1	1	2	3	3	1	1	1	1	2	5	5	4
2541	1	2	-9	3	2	2	2	-9	2	2	1	1	5	1	4
3914	1	1	1	3	3	2	4	2	1	1	2	2	4	5	4

6274	1	3	1	2	2	4	1	3	3	3	2	1	4	1	4
2413	1	2	1	2	2	2	3	1	2	2	2	1	5	1	4
4237	1	2	4	3	2	4	3	2	2	4	2	1	5	1	4
4558	1	2	1	-1	2	1	1	1	1	3	2	1	4	2	4
4901	1	3	2	3	3	4	2	2	2	2	2	1	5	4	4
2097	1	1	2	1	1	3	1	1	1	1	.	1	4	4	4
2699	1	2	2	2	3	3	3	1	1	1	1	1	4	4	4
3660	1	3	2	4	2	3	1	2	3	3	.	1	4	4	4
4952	1	2	2	3	1	3	1	2	1	1	.	1	3	2	4
2102	1	1	1	1	3	1	1	1	1	1	.	1	4	4	4
6531	1	3	1	2	1	4	1	1	1	2	.	1	.	2	4
4087	1	3	-1	3	2	3	2	1	3	3	.	-1	4	2	4
4112	1	4	1	3	2	2	4	3	2	4	.	2	4	5	4
2856	1	-1	-1	3	4	-1	4	2	3	4	2	2	4	5	4
4154	1	3	3	3	3	3	3	3	3	3	2	2	4	4	4
5380	1	2	3	4	4	4	3	2	4	2	2	2	4	4	4
2124	1	2	1	2	2	4	2	3	1	1	2	1	4	4	4
2215	1	2	2	3	-1	3	4	1	2	4	2	2	5	4	4
3588	1	2	2	3	2	4	3	3	4	3	.	2	5	5	4
3703	1	2	1	3	2	2	4	2	3	2	.	2	3	3	4
4217	1	2	1	2	2	3	2	1	1	1	.	1	3	1	4
4720	1	2	1	1	1	1	1	1	-1	1	.	1	5	1	4
3904	1	2	2	1	2	3	1	1	2	1	.	1	4	4	4
4402	1	1	1	1	1	4	4	1	1	1	.	1	4	8	4
6242	1	1	2	4	3	4	3	4	1	1	.	1	4	2	4
2721	1	2	2	2	2	2	2	2	2	2	.	1	3	6	4
1546	1	1	1	2	1	2	3	1	2	4	2	2	5	5	4
4920	1	2	1	2	2	2	3	3	2	2	.	1	4	2	4
2910	1	2	3	2	1	4	2	3	2	2	1	1	5	1	4
4187	1	4	-1	-1	3	2	3	-1	1	1	2	1	5	4	4
4271	1	1	3	3	4	3	4	3	1	1	.	1	2	1	4
6350	1	2	1	3	2	3	4	2	2	3	.	2	5	4	4
3954	1	2	2	2	2	3	1	1	1	2	.	2	4	1	4
6190	1	1	1	1	1	2	1	1	1	1	.	1	5	1	4
6363	1	4	3	4	3	3	2	2	2	3	2	1	5	2	4
1067	1	1	1	1	2	4	1	1	2	2	.	1	4	2	4

4203	1	3	2	3	3	4	2	2	4	1	1	-1	.	1	4
3692	1	2	2	3	3	3	2	1	1	1	1	2	4	8	4
4966	1	1	1	1	1	3	4	1	1	1	.	1	5	4	4
6792	1	2	2	3	2	3	3	2	2	3	1	2	4	4	4
5005	1	2	2	3	4	4	4	3	2	2	2	1	4	1	4
4007	1	3	1	3	3	1	2	4	3	3	1	1	4	2	4
4140	1	2	1	2	2	4	2	3	2	2	2	1	4	1	4
6658	1	2	3	3	3	4	2	3	2	3	.	1	4	1	4
2701	1	2	2	3	4	2	3	2	1	3	.	2	5	3	4
4001	1	2	1	2	2	3	1	1	1	1	1	1	2	1	4
4768	1	2	1	3	4	3	4	1	1	2	.	1	4	4	4
5721	1	1	1	2	2	3	1	2	1	1	1	1	1	2	4
4039	1	2	2	3	1	2	3	2	2	2	1	2	4	1	4
5390	1	3	3	3	3	1	2	3	3	3	1	1	4	4	4
6263	1	2	2	2	2	2	2	2	2	2	2	2	4	4	4
6418	1	2	2	4	1	4	1	1	1	1	1	1	5	2	4
782	1	1	1	2	1	1	1	1	1	1	1	1	2	2	4
4061	1	1	1	2	2	1	3	1	1	1	2	2	2	3	4
4003	1	3	3	4	4	3	4	4	4	4	2	1	3	1	4
3638	1	1	1	1	1	1	1	1	1	1	1	1	5	4	4
4470	1	2	3	4	3	3	3	1	1	2	2	1	5	5	4
4460	1	3	3	3	3	3	4	1	2	2	1	1	5	8	4
6469	1	2	3	3	3	3	2	3	2	2	2	1	4	4	4
3996	1	3	1	1	1	1	1	1	1	1	1	1	2	1	4
4988	1	2	1	3	3	3	3	2	1	3	.	1	4	3	4
4102	2	1	1	2	1	4	3	1	1	1	.	2	4	1	.
2784	3	2	1	3	2	-1	2	1	1	1	.	1	5	1	.
2711	3	3	1	3	3	4	2	2	4	3	1	1	.	1	.
6005	2	3	2	2	3	3	3	2	3	2	.	2	5	1	.
6227	3	2	1	3	3	4	4	1	1	2	.	1	4	3	.
5247	2	2	2	2	3	3	2	1	1	1	1	1	3	2	.
4345	1	2	3	2	2	4	1	2	2	3	.	1	4	1	-2
6864	1	1	1	1	1	3	2	1	1	1	.	2	1	1	1
1417	1	2	4	2	2	3	2	1	1	1	.	2	4	1	1
2236	1	1	1	1	1	3	1	1	1	1	.	2	.	1	1
6613	1	2	2	2	2	2	2	1	2	2	.	2	4	5	1

839	1	1	1	2	2	2	2	1	1	1	1	1	2	1	1
5006	1	1	1	2	3	1	1	1	1	1	.	1	2	1	1
4518	1	1	3	4	3	2	2	1	1	1	.	1	4	2	2
4202	1	1	1	1	1	1	1	1	1	1	.	2	3	1	2
4051	1	2	2	2	1	2	2	1	2	1	.	1	.	1	2
1294	1	3	3	3	3	4	3	1	2	1	.	2	5	3	2
6814	1	2	2	2	3	4	4	3	2	2	.	2	4	3	2
1198	1	1	4	2	2	4	1	1	1	1	.	1	3	2	2
5999	1	1	1	3	3	2	1	1	2	1	.	2	5	1	2
3347	1	1	1	1	1	3	3	3	1	1	1	-1	.	2	3
3995	1	2	2	3	3	4	3	2	2	2	.	1	5	1	3
3745	1	2	1	3	2	3	3	1	2	2	.	1	3	4	3
3409	1	2	2	-1	2	-1	1	3	1	-1	.	-1	4	5	4
5305	2	2	1	2	2	1	3	1	1	1	.	2	3	2	.
6856	1	2	1	3	3	2	3	1	1	2	2	1	4	4	1
2615	1	1	1	1	1	3	1	1	1	1	.	1	3	2	1
6770	1	1	1	2	2	3	2	2	1	1	1	1	1	4	1
5295	1	2	1	2	1	3	2	4	1	2	.	1	2	1	2
5891	1	2	3	3	2	2	3	-1	2	2	.	1	4	1	2
6494	1	2	3	2	2	3	2	3	2	2	.	1	4	3	2
1514	1	1	1	1	1	4	2	1	1	1	.	2	5	2	2
4224	1	1	1	1	1	1	1	1	1	1	1	1	5	1	2
6003	1	2	2	2	2	1	2	2	1	2	.	2	5	1	2
1612	1	2	2	2	2	3	2	2	2	2	.	1	4	4	2
2811	1	2	1	3	3	3	3	1	1	3	.	2	5	1	3
1513	1	1	1	3	1	3	3	3	2	2	.	1	4	1	3
1880	1	1	1	2	1	3	2	1	1	1	.	1	2	8	4
1808	2	2	3	3	4	1	4	2	2	2	.	1	4	1	.
4221	2	1	1	1	1	4	1	1	1	1	.	1	4	3	.
5916	2	2	2	2	2	2	2	2	1	1	.	2	4	4	.
5778	2	2	1	2	3	3	3	1	2	2	.	1	4	1	.
5849	2	1	3	3	3	2	2	2	2	2	.	1	4	1	.
5511	2	2	1	3	3	1	3	1	1	1	1	1	4	2	.
1623	1	2	1	2	1	1	2	1	1	1	.	1	4	2	1
1216	1	3	2	3	3	1	3	3	1	1	.	2	4	2	1
5730	1	1	1	2	1	3	1	1	2	1	.	2	4	1	1

6643	1	2	1	3	3	1	1	1	1	1	1	2	5	1	1
1396	1	1	1	1	1	1	1	1	2	1	.	2	4	1	1
3156	1	2	1	3	3	3	2	1	1	2	.	2	5	1	1
2306	1	1	1	1	1	1	1	1	1	1	.	1	4	1	1
3807	1	1	2	3	2	2	4	2	1	1	.	1	1	8	2
4233	1	2	2	2	2	3	3	1	2	1	.	2	5	4	2
1900	1	2	2	3	3	1	2	2	1	2	.	2	4	4	2
5792	1	2	1	2	2	2	3	2	2	2	.	2	5	4	2
1277	1	2	1	1	1	1	1	1	1	1	.	2	4	1	2
1259	1	2	1	3	2	2	2	2	2	2	2	1	4	2	2
3583	1	1	1	2	2	4	2	1	1	1	.	2	4	2	2
4441	1	1	1	1	1	1	1	1	1	1	.	2	4	3	2
5780	1	2	2	2	2	2	2	1	1	2	.	1	5	1	2
4320	1	1	2	3	2	2	3	1	1	1	.	1	5	1	2
1609	1	1	1	2	3	2	2	1	1	1	.	2	4	1	2
2791	1	2	1	3	2	3	3	1	4	1	2	1	4	1	3
2290	1	2	1	3	3	2	3	2	1	1	.	1	3	8	3
5997	1	1	1	2	2	4	1	1	1	1	.	2	4	3	4
961	3	2	1	2	2	2	3	2	1	1	.	1	2	1	.
2126	2	3	1	3	3	1	2	4	4	4	2	1	4	8	.
3772	2	1	2	2	2	2	3	2	2	2	2	1	4	4	.
5619	1	1	1	1	1	2	1	1	1	1	.	2	3	1	1
2148	1	1	1	2	2	2	3	1	1	1	.	2	5	2	2
2288	1	1	1	2	2	3	1	1	1	1	.	1	3	2	2
3510	1	1	1	2	2	4	2	1	1	1	.	2	.	1	2
5991	1	1	1	3	1	2	2	2	1	1	.	2	4	2	2
2609	1	1	1	1	1	1	1	1	1	1	.	1	2	1	3
2144	1	3	3	4	3	3	2	1	4	3	.	1	4	1	4
4238	3	3	2	3	3	3	3	2	2	2	.	1	4	3	.
1817	2	1	1	1	2	3	2	1	2	2	.	1	4	3	.
2506	2	2	1	3	2	2	1	1	1	1	.	2	5	3	.
3781	3	1	1	3	3	3	4	1	2	1	.	2	5	3	.
5332	2	2	-1	-1	-1	3	-1	-1	-1	-1	2	1	5	4	.
5976	2	2	1	2	1	1	3	1	1	1	.	1	4	1	.
3561	2	2	1	2	2	2	3	1	2	1	.	2	5	1	.
6496	3	1	3	1	1	3	2	2	1	2	.	1	2	1	.

5415	2	2	1	2	2	3	2	4	1	1	.	1	3	4	.
973	3	1	1	1	1	2	3	2	1	1	.	1	3	1	.
1169	3	2	1	3	3	3	3	3	3	3	.	1	4	1	.
6931	3	3	2	3	2	2	3	4	3	2	.	2	5	1	.
6153	1	1	3	1	1	3	2	4	1	1	.	2	3	1	1
3810	1	2	1	3	2	4	3	1	1	1	.	2	5	1	1
5909	1	1	1	2	2	4	2	1	1	1	.	1	4	2	1
6184	1	1	1	2	3	2	3	1	1	1	.	1	2	1	1
2553	1	2	1	2	2	3	1	1	1	1	1	1	4	1	1
3782	1	2	1	4	3	4	2	1	2	1	.	2	2	2	2
4426	1	2	1	2	1	2	1	1	1	1	.	1	4	2	2
1767	1	1	1	2	3	4	2	1	1	1	.	1	2	1	2
3044	1	2	1	1	1	3	2	1	1	1	.	1	2	1	2
3601	1	1	1	3	1	4	1	1	1	1	.	1	4	1	2
5952	1	1	1	2	1	1	2	1	1	1	.	2	4	1	2
6033	1	1	1	1	1	1	1	1	1	1	.	2	4	1	2
6201	1	2	2	2	3	3	2	2	2	2	.	1	4	1	2
1563	1	3	1	2	1	3	3	1	1	1	.	2	5	1	2
3763	1	2	1	1	2	4	2	3	1	2	.	1	4	3	3
1614	1	2	2	2	2	2	2	1	1	1	.	1	5	1	3
4488	1	3	1	3	2	4	4	4	2	3	.	1	4	1	3
2294	3	2	1	2	2	2	2	1	1	3	.	1	4	1	.
5706	1	2	2	3	2	2	3	1	1	2	.	1	1	2	1
1755	1	1	4	4	2	1	1	1	1	1	.	2	4	2	1
5865	1	1	1	3	1	3	3	1	1	1	.	2	3	1	2
4696	1	2	3	3	1	3	2	1	2	3	.	1	5	8	4
6370	3	2	-1	4	3	3	3	-1	-1	-1	.	1	5	1	.
6582	2	1	1	2	1	2	1	1	1	1	.	1	3	1	.
3771	3	1	1	3	1	1	1	3	1	1	.	2	5	3	.
3157	1	2	1	3	2	3	2	2	2	2	.	1	4	1	1
3429	1	2	1	4	2	3	2	1	1	1	.	2	5	2	1
1394	1	1	1	2	1	2	1	1	1	1	.	1	4	1	1
1629	1	1	1	2	2	4	2	3	3	3	.	1	4	2	2
1630	1	2	4	4	3	3	2	3	1	2	.	1	5	1	2
1982	1	1	1	1	1	3	2	2	1	1	.	1	4	1	2
2402	1	2	3	3	3	4	3	2	2	2	.	1	4	1	2

4025	1	1	4	2	3	3	2	2	2	2	.	1	2	1	2
6942	1	2	1	2	1	1	1	1	1	1	.	1	2	1	2
4371	1	1	1	3	4	3	4	1	1	1	.	1	4	1	2
1112	1	1	1	3	2	4	2	3	1	1	.	2	4	1	2
4491	1	2	1	3	1	2	2	1	1	1	1	2	5	2	2
1385	1	2	2	3	2	4	2	1	4	2	.	1	4	1	2
5758	1	2	1	2	2	1	2	1	1	1	.	2	4	4	2
5829	1	1	1	2	1	2	2	1	1	1	.	1	4	1	2
4471	1	2	2	3	2	2	1	1	2	2	.	1	4	1	3
5714	1	2	3	2	3	3	3	2	2	4	.	1	3	4	3
1498	1	2	2	3	3	4	4	4	3	2	.	2	5	1	3
2183	1	2	2	2	3	2	2	1	1	3	1	2	5	5	4
1785	3	1	1	3	1	1	1	1	1	1	.	1	2	1	.
5776	3	1	1	2	2	3	2	1	1	1	.	2	2	1	.
4056	2	2	1	3	1	2	1	1	1	1	.	1	4	1	.
5742	2	1	1	1	1	4	2	1	1	1	.	2	3	8	.
6095	3	2	1	2	1	1	2	1	3	1	.	2	5	1	.
2168	3	1	1	1	1	2	1	1	2	1	.	1	5	3	.
3742	3	1	1	2	2	1	1	1	3	1	.	1	2	1	.
780	2	2	4	3	2	2	2	2	4	3	.	2	5	1	.
3084	2	2	1	2	1	3	1	1	1	2	.	1	2	1	.
3700	2	1	1	3	2	3	4	2	1	3	.	2	4	4	.
887	3	2	1	3	1	3	2	1	1	2	.	1	4	2	.
1338	1	2	1	3	3	4	3	2	2	2	.	1	4	2	1
2453	1	2	1	1	2	2	1	1	1	1	.	2	3	1	1
1318	1	2	1	3	2	3	3	2	3	1	.	1	5	1	1
1354	1	2	1	3	2	4	2	1	1	1	.	2	4	1	2
3481	1	1	1	2	1	3	1	1	1	1	.	1	4	1	2
790	1	1	1	3	2	4	1	1	1	1	.	1	5	1	3
4945	1	2	1	1	1	2	2	3	4	1	.	2	5	1	3
1515	1	2	1	2	1	3	2	1	1	1	.	2	4	2	3
1529	1	1	1	3	2	4	2	1	1	1	.	1	3	1	4
3746	1	2	3	3	3	3	2	1	2	2	.	2	4	1	4
6835	1	2	2	3	2	2	2	1	1	1	.	1	5	3	4
5634	2	1	1	3	3	2	3	2	1	1	.	1	2	4	.
3919	1	2	2	4	3	1	1	1	3	3	.	2	4	8	4

1569	2	2	2	3	3	3	3	2	2	2	.	1	4	3	.
1193	2	2	2	3	2	2	2	2	2	2	.	2	3	1	.
5517	2	1	1	1	2	3	1	1	1	1	.	1	4	2	.
890	3	2	3	4	3	3	3	3	1	2	.	1	.	2	.
1339	3	2	1	2	1	1	1	1	1	1	.	2	5	1	.
3487	2	2	1	2	2	3	1	1	2	2	.	2	5	1	.
2919	2	1	1	3	1	1	2	1	1	1	.	1	2	1	.
1389	3	2	-1	2	2	2	1	1	1	1	.	2	4	1	.
3112	3	2	1	3	3	4	3	1	2	1	.	-1	3	1	.
3148	2	1	1	1	1	1	1	1	1	1	.	2	4	1	.
6252	3	1	2	3	2	3	2	1	1	1	.	2	4	1	.
5766	1	1	4	3	1	4	1	2	1	1	.	1	.	-1	-2
3926	1	2	-1	2	1	4	3	2	2	1	.	2	3	1	1
1165	1	1	1	3	2	2	2	1	1	1	.	2	4	1	2
6667	1	1	1	3	2	2	2	1	1	1	.	1	4	1	2
3685	1	1	1	1	1	3	1	1	1	1	.	1	4	2	2
3641	1	1	1	1	2	4	3	1	2	2	.	1	.	4	2
4302	1	-1	4	4	2	4	-1	3	-1	-1	.	2	5	1	2
6105	1	2	2	2	2	2	2	2	2	2	.	1	4	3	3
3574	1	1	1	3	2	2	1	2	1	1	.	1	1	2	3
1782	1	1	-1	1	1	3	1	4	1	1	.	2	2	2	3
3963	1	1	1	2	4	3	3	1	1	1	.	2	3	1	3
3846	3	2	1	1	1	2	1	1	1	1	.	2	4	1	.
2926	2	2	1	3	2	3	3	1	2	2	.	1	4	1	.
1252	2	1	1	3	2	3	2	1	2	1	.	2	.	1	.
1521	2	1	1	1	1	3	1	1	1	1	.	1	3	3	.
1633	2	2	1	2	1	2	3	1	1	1	.	1	4	4	.
2471	3	2	2	2	3	2	2	3	3	2	.	1	4	2	.
5690	3	2	2	4	3	2	1	1	3	2	.	2	5	3	.
1478	3	2	1	1	1	1	1	1	1	1	.	2	4	1	.
5831	1	2	1	3	3	3	3	2	2	2	.	2	4	2	2
5995	1	4	-1	4	4	3	3	4	3	3	.	2	5	8	2
3675	1	1	1	1	1	2	2	1	-1	-1	.	2	5	1	2
921	1	1	2	2	1	3	2	1	1	1	.	1	4	1	2
1276	1	1	2	2	2	2	2	1	1	1	.	1	4	2	2
1793	1	2	1	3	3	3	3	1	1	3	.	1	4	4	2

3735	1	2	3	3	1	3	1	1	1	1	.	1	4	1	3
2728	1	2	1	3	3	3	2	1	1	1	.	1	4	3	3
4206	1	1	1	2	2	1	1	1	1	1	.	1	4	2	4
3606	1	1	1	1	1	1	2	1	1	1	.	2	4	1	4
5985	1	1	1	2	1	3	1	1	1	1	.	1	2	1	3
